# A dual role of *lola* in *Drosophila* ovary development: regulating stem cell niche establishment and repressing apoptosis

**DOI:** 10.1038/s41419-022-05195-9

**Published:** 2022-09-02

**Authors:** Ting Zhao, Yanhong Xiao, Bo Huang, Mao-Jiu Ran, Xin Duan, Yu-Feng Wang, Yuzhen Lu, Xiao-Qiang Yu

**Affiliations:** 1 School of Life Sciences, Hubei Key Laboratory of Genetic Regulation and Integrative Biology, Central China Normal University, Wuhan, PR China; 2 Guangdong Provincial Key Laboratory of Insect Developmental Biology and Applied Technology, Guangzhou Key Laboratory of Insect Development Regulation and Application Research, Institute of Insect Science and Technology, South China Normal University, Guangzhou, PR China

**Keywords:** Apoptosis, Stem-cell niche

## Abstract

In *Drosophila* ovary, niche is composed of somatic cells, including terminal filament cells (TFCs), cap cells (CCs) and escort cells (ECs), which provide extrinsic signals to maintain stem cell renewal or initiate cell differentiation. Niche establishment begins in larval stages when terminal filaments (TFs) are formed, but the underlying mechanism for the development of TFs remains largely unknown. Here we report that transcription factor longitudinals lacking (Lola) is essential for ovary morphogenesis. We showed that Lola protein was expressed abundantly in TFCs and CCs, although also in other cells, and *lola* was required for the establishment of niche during larval stage. Importantly, we found that knockdown expression of *lola* induced apoptosis in adult ovary, and that *lola* affected adult ovary morphogenesis by suppressing expression of *Regulator of cullins 1b* (*Roc1b*), an apoptosis-related gene that regulates caspase activation during spermatogenesis. These findings significantly expand our understanding of the mechanisms controlling niche establishment and adult oogenesis in *Drosophila*.

## Introduction

*Longitudinals lacking* (*lola*) is a complex gene in *Drosophila melanogaster*, encodes at least 20 protein isoforms (Lola A – Lola T) [1–3]. All the Lola isoforms share an N-terminal Broad-Complex, Tramtrack and Bric-à-brac (BTB) domain that is implicated in protein–protein interaction, and 17 isoforms contain unique C-terminal zinc finger (ZF) motifs involved in binding with specific DNA [4–6]. Lola has been shown to play a role in regulating adult midgut homeostasis [7], axon growth and guidance [3, 8, 9], male germline and neuron stem cell maintenance and differentiation [10, 11], embryonic gonad formation and programmed cell death during oogenesis [12, 13]. According to the modENCODE Tissue Expression Data, *lola* is abundantly expressed in *Drosophila* ovary (http://flybase.bio.indiana.edu). However, its role in ovarian development has received little attention.

**Fig. 1:**
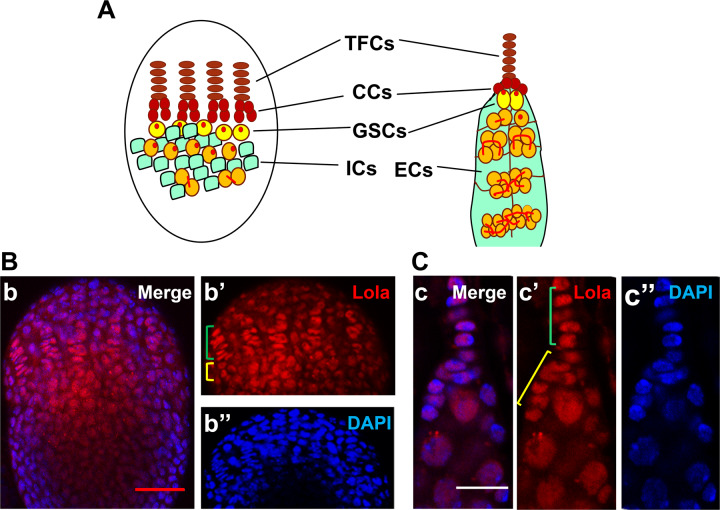
Lola protein is expressed in terminal filament cells and cap cells of WPP and adult ovaries. **A** Schematic diagram of a *Drosophila* ovary in WPP (left) and adults (right) with cell types labeled. TFCs, Terminal Filament Cells; CCs, Cap Cells; GSCs, Germline Stem Cells; ICs, Intermingled Cells; ECs, Escort Cells. **B, C** Localization of Lola protein in the WPP ovary **B** and adult ovary **C**. Lola protein was detected by anti-Lola antibody (**b**’ and **c**’) and nuclei were stained by DAPI (**b**” and **c**”). Lola protein accumulates in the nuclei of TFCs and CCs (green and yellow square brackets, respectively, in **b**’ and **c**’). Scale bars: 50 µm in **B** and 5 µm in **C**.

The *Drosophila* ovary is a powerful model for studying genetics and mechanisms that program maintenance of stem cell niche and development of adult ovary. *Drosophila* female has a pair of ovaries. Each ovary is composed of 16–20 ovarioles, and an ovariole contains several egg chambers in different developmental stages [14]. Germline stem cells (GSCs) have resided in the tip of germarium, a structure situated at the apical end of an ovariole. GSCs and their niches constitute functional units to produce eggs to maintain female reproductive capacity. Ovarian GSC niche is composed of several types of somatic cells: terminal filament cells (TFCs), cap cells (CCs) and anterior escort cells (ECs) (Fig. 1A) [15–17]. These cells provide extrinsic signals to GSCs to maintain stem cell identity [18–20]. Terminal filament (TF) formation occurs during the larval stage, and is the beginning point of GSC niche establishment [21]. By the late third instar larval (LL3) and white pre-pupal (WPP) stages, TFCs complete flattening, sorting, intercalation and stacking, and form well-organized TF stacks (Fig. 1A) [22, 23]. The number of GSC niches or ovarioles in adult flies is equal to the number of TFs that form in the larval ovary [24]. At present, only a few genes, including *bric-à-brac* (*bab1*/*bab2*), *Lmx1a* and *engrailed*/*invected* (*en*/*inv*), have been reported to be expressed in TFCs and CCs and are involved in the formation of TFs [18, 25, 26]. The genetic events coordinating TF formation and function are still largely unknown.

In adults, oogenesis process is divided into 14 stages according to specific morphological characteristics [14]. It has been reported that programmed cell death (PCD) occurs during early, middle and late stages of oogenesis [27, 28]. In response to starvation, germline cyst cells may undergo PCD within the germarium, or egg chambers may be degenerated at stage eight [27]. In the late stage of oogenesis, nurse cells ‘dump’ their cytoplasmic contents into the oocyte and undergo PCD [28].

In the present study, we determined the localization of Lola protein in TFCs and CCs of the ovary. We showed that knockdown expression (RNAi) of *lola* impaired female fertility and ovary morphogenesis. Most ovaries from the *lola* RNAi lines were significantly smaller in size and did not contain mature eggs. All the abnormal smaller ovaries had fewer ovarioles or lacked distinguishable ovarioles because RNAi of *lola* impeded TFs formation in the larval stage. RNAi of *lola* also led to ovarian apoptosis in adults, which was not starvation-induced PCD nor late stage nurse cell death. Moreover, we showed that *lola* plays an essential role in *Drosophila* ovary development by suppressing expression of an apoptosis-related gene, *Regulator of cullins 1b* (*Roc1b)*. Taken together, our findings reveal that *lola* is a novel effector in *Drosophila* to regulate niche establishment in the larval stage and ovarian apoptosis in the adult stage.

## Results

### Lola protein is expressed in the developing and adult ovaries

Specific localization of Lola protein in ovary has not been previously reported, although Western blot analysis showed that Lola was present in the adult ovary [12]. Using a Lola antibody to the common BTB domain, we showed that Lola protein was present in larval ovary and adult ovarian germarium (Fig. 1B, C). In both the larval and adult ovaries, Lola protein was abundant in the terminal filament cells (TFCs) and cap cells (CCs) that constitute the GSC niche, and it was also detected in other cells (Fig. 1B (b’), C (c’)), implying that *lola* may play a role in the formation of GSC niche.

### *Lola* is required for female fertility

**Fig. 2:**
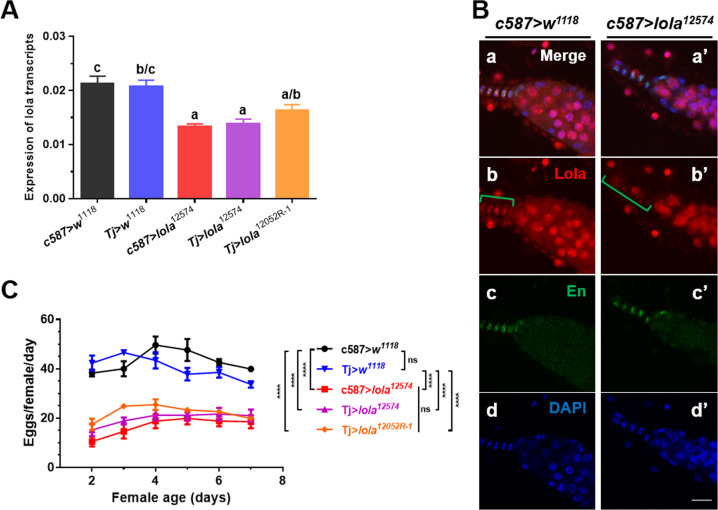
*Lola* is required for female fertility. **A** Expression of *lola* transcripts in the ovaries of the *c587* > *lola*^*12574*^, *Tj* > *lola*^*12574*^, *Tj* > *lola*^*12052R-1*^, *c587* > *w*^*1118*^ and *Tj* > *w*^*1118*^ fly lines. For each graph, different letters above bars indicate groups significantly differed from one another (*p* < 0.05, one-way ANOVA followed by a post hoc Tukey’s honestly significant difference [HSD] test using GraphPad). **B** Adult ovaries from the control *c587* > *w*^*1118*^ line (**a-d**) and *c587* > *lola*^*12574*^ RNAi line (**a**’**-d**’) were immunostained with Lola and En antibodies, and nuclei were stained with DAPI. In the *c587* > *lola*^*12574*^ RNAi line, reduced Lola protein level was observed in TFCs compared to the *c587* > *w*^*1118*^ line (green brackets in **b** and **b**’). **C** Female fertility tests were performed in the above fly lines. Significant difference was determined by one-way ANOVA followed by a post hoc Tukey’s HSD test and indicated by asterisks: **** *p* < 0.0001; ns, non-significant. Scale bar: 10 µm in **B**.

Given that Lola protein is abundant in somatic cells such as TFCs and CCs, we used a somatic driver *c587-Gal4* line mated with a *lola* RNAi line (VDRC 12574) to express inducible *lola* RNAi in somatic cells. qRT-PCR analysis and immunostaining assay revealed that expression of *lola* at both the transcriptional and protein levels was significantly decreased in the ovary of *c587* > *lola*^*12574*^ than in the control *c587* > *w*^*1118*^ (Fig. 2A, B). Importantly, knockdown expression of *lola* driven by *c587-Gal4* in the ovary severely impaired female fecundity (Fig. 2C). To validate *c587* > *lola*^*12574*^ RNAi results, we used another *lola* RNAi line NIG 12052R-1. Since *c587* > *lola*^*12052R-1*^ RNAi was lethal in the pupal stage (data not shown), we then used another somatic driver *Tj-Gal4* to mate with both *lola*^*12574*^ and *lola*^*12052R-1*^ RNAi lines. Both *c587-Gal4* and *Tj-Gal4* drivers are expressed in same somatic cells named intermingled cells (ICs) in the larval ovary, which give rise to adult escort cells (ECs) [29, 30]. qRT-PCR results showed that expression of *lola* transcripts was significantly reduced in the ovary of *Tj* > *lola*^*12574*^ RNAi line than in the *Tj* > *w*^*1118*^ control, and the transcriptional level of *lola* transcripts in the ovary of *Tj* > *lola*^*12052R-1*^ RNAi line was also reduced compared to the *Tj* > *w*^*1118*^ line (Fig. 2A). Knockdown expression of *lola* transcripts driven by both *c587-Gal4* and *Tj-Gal4* in the ovary had similar impacts on female fecundity (Fig. 2C). These results indicated that *lola* is required for female fertility. Since both *lola*^*12574*^ and *lola*^*12052R-1*^ RNAi lines had similar effects on female fertility, we used *lola*^*12574*^ RNAi line in most of our following experiments.

### *Lola* is required for ovariole morphogenesis

**Fig. 3:**
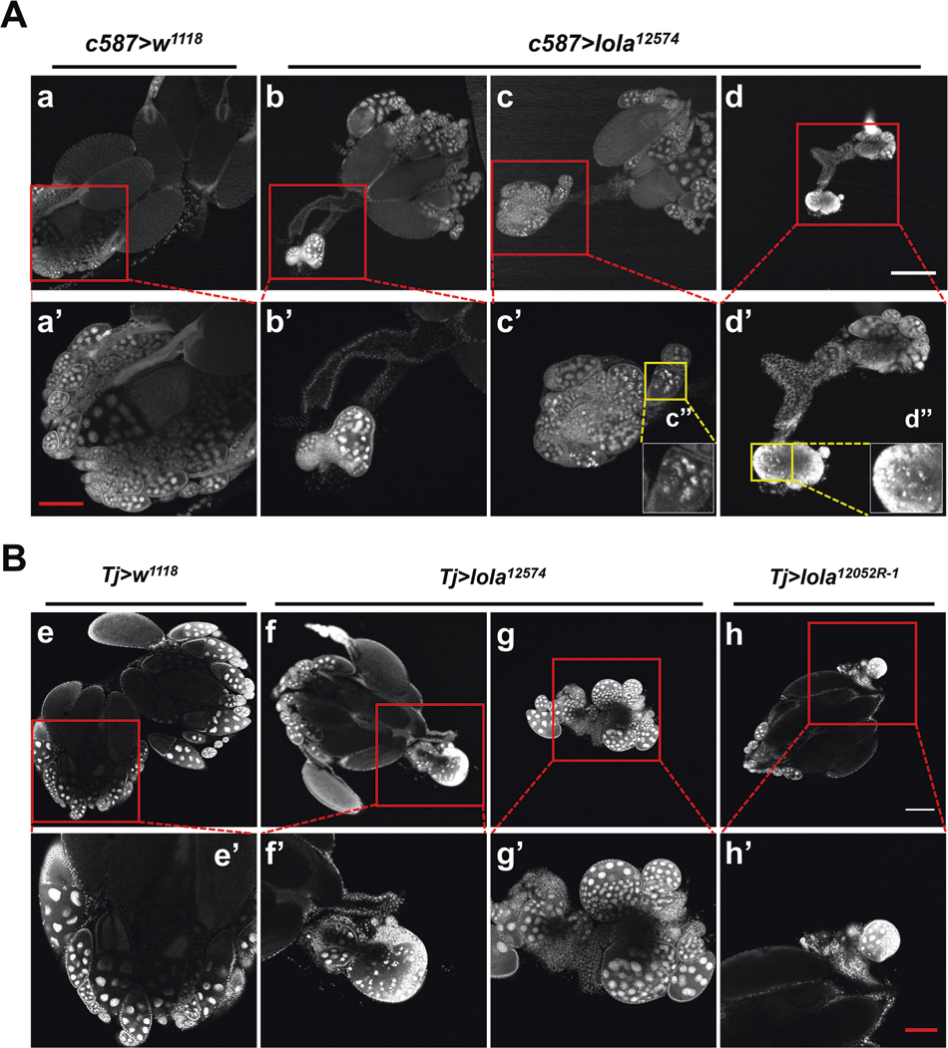
RNAi of *lola* results in smaller ovaries and fewer ovarioles. **A** Phenotypes of ovaries from the *c587* > *w*^*1118*^ and *c587* > *lola*^*12574*^ lines. Ovaries with normal size and number of ovarioles (**a**, **a**’) from the *c587* > *w*^*1118*^ control line (*n* = 25), and ovaries with either one (**b**, **c**) or both (**d**) in smaller size and fewer ovarioles (**c**’) or lacking ovarioles (**b**’, **d**’) from the *c587* > *lola*^*12574*^ RNAi line (*n* = 58). **B** Phenotypes of ovaries from the *Tj* > *w*^*1118*^, *Tj* > *lola*^*12574*^ and *Tj* > *lola*^*12052R-1*^ lines. Ovaries *w*ith normal size and number of ovarioles (**e**, **e**’) from the *Tj* > *w*^*1118*^ control, ovaries with either one (**f**) or both (**g**) in smaller size from the *Tj* > *lola*^*12574*^ RNAi line, and ovaries with one in smaller size (**h**) from the *Tj* > *lola*^*12052R-1*^ RNAi line. **a**’-**h**’ Higher magnifications of the ovary regions from the corresponding ovaries (**a**–**h**). **c**”, **d**” Higher magnifications of the ovary regions from the corresponding ovaries (**c**’, **d**’) showing DNA fragmentations. Scale bar: 200 µm in **a**–**h**, and 100 µm in **a**’–**h**’.

To explore the underlying mechanism that *lola* affects female fertility, ovaries from 3-day-old *c587* > *lola*^*12574*^, *Tj* > *lola*^*12574*^ and *Tj* > *lola*^*12052R-1*^ RNAi lines as well as *c587* > *w*^*1118*^ and *Tj* > *w*^*1118*^ control flies were dissected and stained to examine ovarian development. We found that in the *c587* > *lola*^*12574*^ RNAi females, 48.3% of the ovaries had one ovary with significantly smaller size (Fig. 3A (b, c)), and 27.6% of the ovaries had a pair of smaller ovaries (Fig. 3A (d)). In all the smaller ovaries, either the number of ovarioles decreased (Fig. 3A (c’)) or ovarioles were not observed (Fig. 3A (b’, d’)), and some egg chambers were fused (Fig. 3A (c’)). Similar and slightly less severe phenotypes were observed in the ovaries of *Tj* > *lola*^*12574*^ and *Tj* > *lola*^*12052R-1*^ RNAi females (Fig. 3B). Taken together, these results indicated that *lola* is essential for ovariole morphogenesis.

### *Lola* is required for GSC niche establishment

**Fig. 4:**
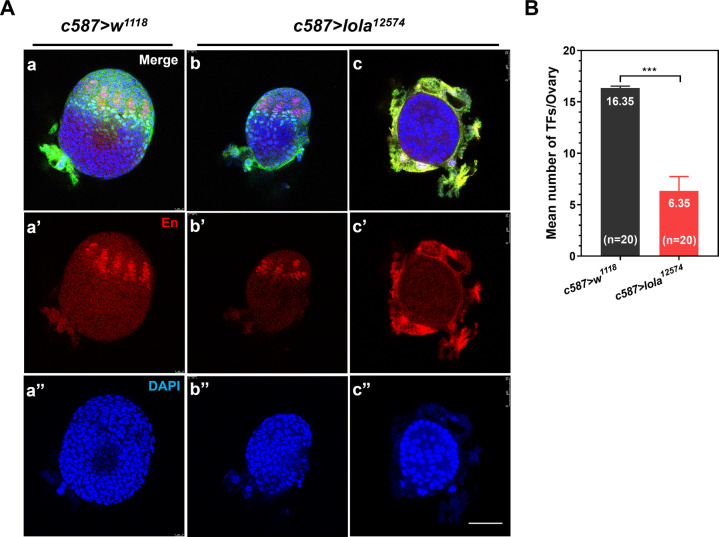
RNAi of *lola* affects TF formation during WPP. **A** Ovaries from the control *c587* > *w*^*1118*^ line (**a)** and *c587* > *lola*^*12574*^ RNAi line (**b**, **c**) at the WPP stage were immunostained with En antibody to mark TFCs (**a**’**-c**’, red), and nuclei were stained with DAPI (**a**”**–c**”, blue). In a control ovary (**a**–**a**”), TFs were well organized, while in *c587* > *lola*^*12574*^ RNAi ovaries, either some TFCs were arranged randomly (**b–b**”) or no TFs were formed (**c–c**”). **B** The mean number of TFs per ovary in *c587* > *lola*^*12574*^ RNAi ovaries is significantly lower than that of the *c587* > *w*^*1118*^ control ovaries (*n* = 20). Significant difference was determined by the student’s t-test and indicated by ***p < 0.001. Scale bar: 30 µm.

Since the number of ovarioles in adult flies matches the number of TFs formed in the larval ovary [24], we speculate that knockdown of *lola* expression may affect the formation of TF in the larval ovary. To investigate this possibility, ovaries from the *c587* > *lola*^*12574*^ RNAi and *c587* > *w*^*1118*^ control lines at the white pre-pupal (WPP) stage were dissected and stained with TF marker Engrailed (En) (Fig. 4). Ovaries from the *c587* > *lola*^*12574*^ RNAi line (Fig. 4A (b, c)) were much smaller than those in the *c587* > *w*^*1118*^ control line (Fig. 4A (a)), a result consistent with that of the adult ovaries (Fig. 3). In the ovary of *c587* > *w*^*1118*^ control line, TF stacks were well organized (Fig. 4A (a’)), while in the ovary of *c587* > *lola*^*12574*^ RNAi line, TF stacks were disordered and the number of TFs was reduced (Fig. 4A (b’)), this is also consistent with the result in adult ovary that smaller ovaries had fewer TFs (Fig. 3A (c’)). In the most severe case, no TF stacks were formed in the ovary of *c587* > *lola*^*12574*^ RNAi line (Fig. 4A (c’)), which matches the morphology in adult ovaries that no distinguishable ovarioles were observed in the smaller ovaries (Fig. 3A (b’, d’)). The number of TFs per ovary in the *c587* > *lola*^*12574*^ RNAi was significantly fewer than that in the *c587* > *w*^*1118*^ control (Fig. 4B). The formation of TFs in the larval stage is the starting point to establish GSC niche, thus *lola* is required for GSC niche establishment.

### *Lola* is involved in regulating ovarian apoptosis in the adult stage

**Fig. 5:**
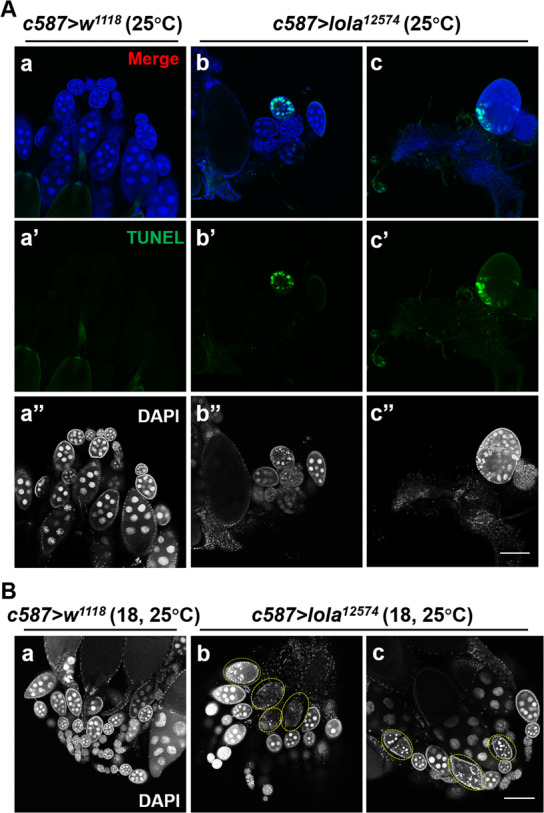
RNAi of *lola* induces ovarian apoptosis. **A** Ovaries from the *c587* > *w*^*1118*^ control line (**a**) and *c587* > *lola*^*12574*^ RNAi line reared at 25 °C from eggs to adults (**b**, **c**) were stained with TUNEL assay for apoptotic cells (**a**’**–c**’, green), and nuclei were stained with DAPI (**a**”**–c**”, white). In a control ovary, cells were TUNEL-negative (**a**–**a**”), while in *c587* > *lola*^*12574*^ RNAi ovaries, TUNEL-positive was observed (**b**–**b**”, **c**–**c**”). **B** The *c587* > *w*^*1118*^ and *c587* > *lola*^*12574*^ RNAi lines were reared at 18 °C from eggs to pupae and then at 25 °C from pupae to adults. Ovaries from 3-4 days *c587* > *w*^*1118*^ and *c587* > *lola*^*12574*^ RNAi adults were dissected and stained with DAPI. DNA fragments were detected in the ovaries of *c587* > *lola*^*12574*^ RNAi adults (**b** and **c**, circled in yellow), but not in the ovaries of *c587* > *w*^*1118*^ control adults (**a**). Scale bar: 100 µm.

We observed some DNA fragments in the smaller ovaries of *c587* > *lola*^*12574*^ RNAi line (Fig. 3A (c”, d”)), and speculate that smaller in size of ovary may be due to apoptosis in the ovarioles. To investigate this, we performed TUNEL (Terminal deoxynucleotidyl Transferase Biotin dUTP Nick End Labeling) and DAPI staining. The presence of DNA fragmentation, as depicted by green staining, was detected in 89.7% of the smaller ovaries of the *c587* > *lola*^*12574*^ RNAi line (*n* = 29) (Fig. 5A (b’, c’)) but not in the ovaries of the *c587* > *w*^*1118*^ control line (Fig. 5A (a’)). To determine whether these phenotypes were due to loss of Lola function during ovary development or in the adult stage, we utilized the well-established characteristics of Gal4 driver, which has low activity at low temperatures but increased activity at higher temperatures. The *c587* > *lola*^*12574*^ and *c587* > *w*^*1118*^ flies were reared at 18 °C from eggs to pupae with functional niches [30], and then reared at 25 °C from pupae to adults to knock down *lola* expression. Ovaries from 3 to 4 days adults were dissected and stained, and the results showed that ovaries from the *c587* > *lola*^*12574*^ RNAi adults under these conditions had normal size and proper number of ovarioles (Fig. 5B (b, c)) compared to those of the *c587* > *w*^*1118*^ control adults (Fig. 5B (a)). However, in 43% of ovaries from the *c587* > *lola*^*12574*^ RNAi adults (*n* = 44), about 3–6 egg chambers showed DNA fragments during mid-oogenesis (Fig. 5B (b, c)), while in ovaries of the *c587* > *w*^*1118*^ control adults, no more than three egg chambers showed DNA fragments (Fig. 5B (a)). These results suggest that *lola* represses apoptosis in *Drosophila* adult ovary.

### *Lola* regulates gene expression in the ovary

**Fig. 6:**
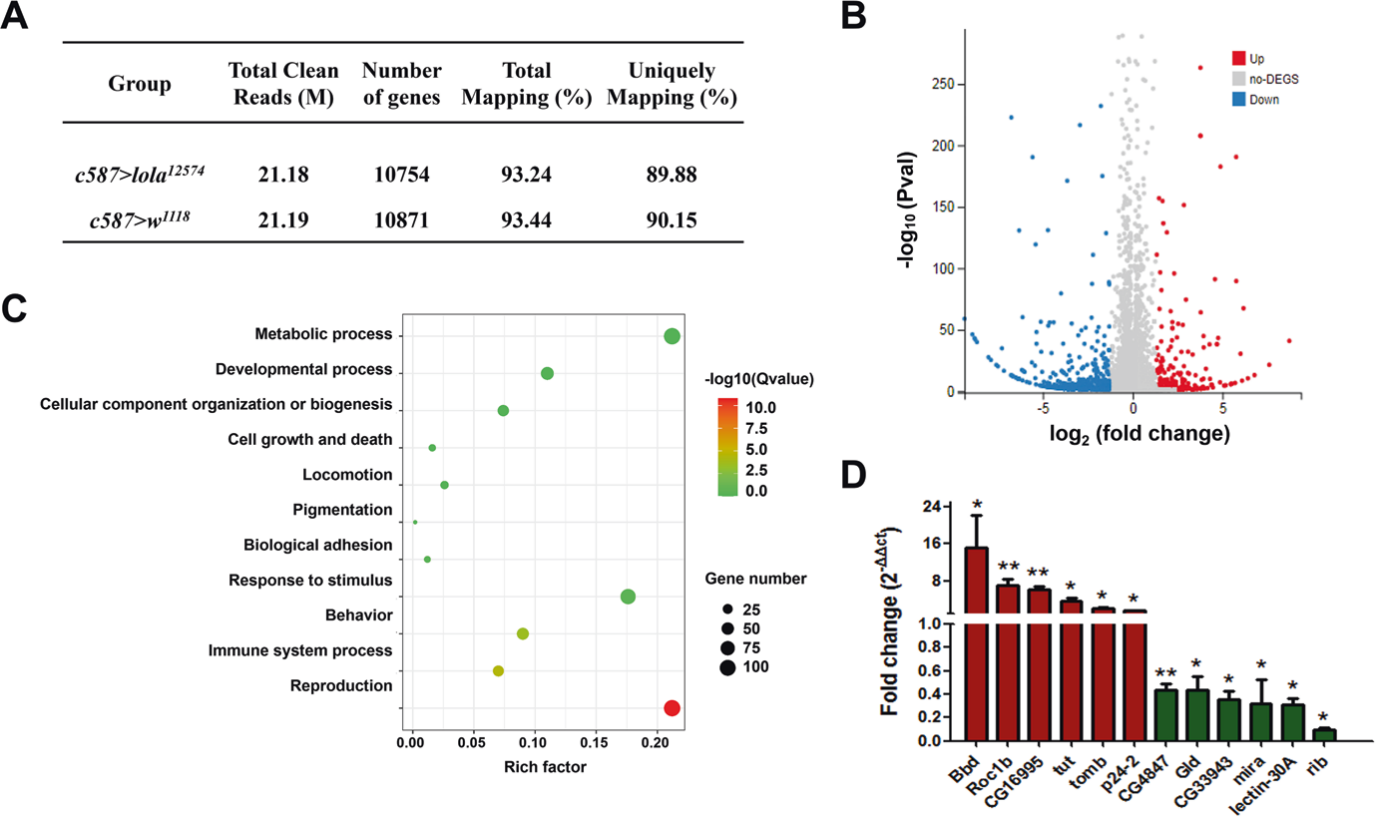
RNAi of *lola* in the ovary results in transcript alterations as assessed by RNA-seq analysis. **A** The numbers of total clean reads, number of genes, total mapping and unique mapping for each sample. **B** Volcano plot of differentially expressed genes (DEGs) in the ovary of *c587* > *lola*^*12574*^ RNAi line relative to the *c587* > *w*^*1118*^ control. **C** GO analysis of DEGs from comparison of the *c587* > *lola*^*12574*^ RNAi and *c587* > *w*^*1118*^ control groups. **D** qRT-PCR validation of select DEGs from RNA sequencing data. Significant difference was determined by the student’s t-test and indicated by *p < 0.05 and **p < 0.01.

To better understand how *lola* plays such an important role in regulating ovarian development, we carried out RNA-sequencing (RNA-Seq) with RNAs isolated from the ovaries of *c587* > *w*^*1118*^ control and *c587* > *lola*^*12574*^ RNAi females. We identified a total of 12695 genes by RNA-Seq (Supplementary Table S1). The numbers of clean reads, expressed genes, total mapped reads and unique matches for each sample were shown in Fig. 6A. Compared with the control, a total of 433 genes have at least 1.3-fold change (q-value < 0.05) in the expression levels, of which, 152 genes were upregulated and 281 genes were downregulated in the ovary of *c587* > *lola*^*12574*^ RNAi line (Fig. 6B, Supplementary Tables S2, S3). This result indicated that Lola serves as a transcription factor in the ovary. Gene Ontology (GO) analysis showed that 106 differentially expressed genes (DEGs) were involved in reproduction and 106 DEGs were also involved in metabolic process (Fig. 6C). Among the reproduction-related DEGs, many have been shown to play a role in gonad development, oogenesis and oviposition, such as *rib*, *mira*, *p24-2* and *BG642312* [31–34]. There are also 8 DEGs involved in cell growth and death (Fig. 6C), including *Roc1b* and *tomb* [35, 36].

To confirm RNA-seq data, 12 differentially expressed genes associated with reproduction and cell growth and death were selected for qRT-PCR analysis (Table 1). The results showed that expression profiles of all the select DEGs in the ovary measured by qRT-PCR were consistent with those of the RNA-seq data, with 6 DEGs (*Bbd*, *Roc1b*, *CG16995*, *tut*, *tomb*, and *p24-2*) upregulated and 6 DEGs (*CG4847*, *Gld*, CG33943, *mira*, *lectin-30A* and *rib*) downregulated (Fig. 6D). Taken together, these results suggest that *lola* regulates expression of genes involved in reproduction, cell growth and death.

**Table 1:** Differentially expressed genes selected for qRT-PCR validation.

Relative expression	Gene symbol	Log_2_ Fold difference	Biological functions
Up-regulated	tut	1.34	Regulation of mitotic amplification of germ cells
	Roc1b	1.41	Involved in caspase activation during spermatogenesis
	Bbd	1.49	Involved in multicellular organism reproduction
	p24-2	1.71	Involved in post-mating oviposition
	tomb	1.86	Meiotic-arrest gene involved in spermatogenesis
	CG16995	2.39	Involved in multicellular organism reproduction
Down-regulated	BG642312	−1.62	Regulation of oviposition
	mira	−1.74	Involved in oogenesis
	rib	−1.71	Regulation of gonad development
	Gld	−2.1	Involved in sperm storage
	CG4847	−2.48	Involved in multicellular organism reproduction
	lectin-30A	−4.58	Involved in multicellular organism reproduction

### *Lola* regulates ovarian morphogenesis by suppressing *Roc1b* expression

**Fig. 7:**
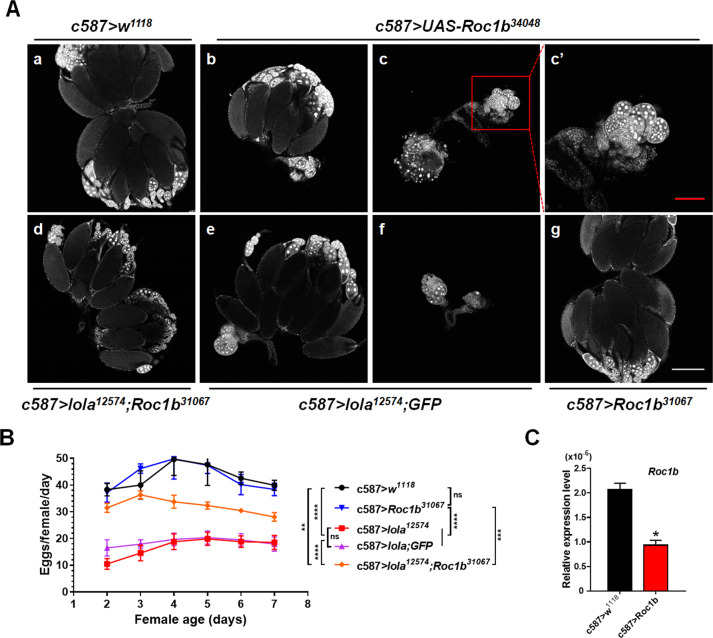
*Lola* regulates ovarian morphogenesis by suppressing *Roc1b* expression. **A** Phenotypes of ovaries from the *c587* > *w*^*1118*^, *c587* > *UAS-Roc1b*^*34048*^, *c587* > *lola*^*12574*^*;Roc1b*^*31067*^, *c587* > *lola*^*12574*^*;GFP* and *c587* > *Roc1b*^*31067*^ lines. **a** Ovaries with normal size and number of ovarioles from the *c587* > *w*^*1118*^ control. **b**, **c** Overexpression of *Roc1b* resulted in one smaller ovary with fewer ovarioles (**b**) or a pair of smaller ovaries with fused egg chambers (**c**), which mimic the phenotypes of *lola*^*12574*^ RNAi. **d** Knockdown of *Roc1b* in the *c587* > *lola*^*12574*^ RNAi line rescued defects in ovarian morphogenesis. **e**, **f** Knockdown of *GFP* in the *c587* > *lola*^*12574*^ RNAi line resulted in one smaller ovary with fewer ovarioles (**e**) or a pair of smaller ovaries with fused egg chambers (**f**). **g** Ovaries with normal size and number of ovarioles from the *c587* > *Roc1b*^*31067*^. **c**’ Higher magnification of the ovary region from **c**. **B** Knockdown expression of *Roc1b* in flies (*c587* > *Roc1b*^*31067*^) did not have an effect on female fertility, while knockdown expression of *Roc1b* in the *c587* > *lola*^*12574*^ RNAi line increased female fertility. **C** Expression of *Roc1b* in the ovaries of the *c587* > *Roc1b*^*31067*^ and *c587* > *w*^*1118*^ control lines. Significant difference was determined by the student’s t-test and indicated by *p < 0.05, **p < 0.01, ***p < 0.001, ****p < 0.0001, ns for non-significant. Scale bar: 100 µm in **a**–**g**, 300 µm in **c**’.

Given that knockdown of *lola* in the ovary induced apoptosis, we pay attention to one DEG (*Roc1b*) that is related to apoptosis. It has been shown that *Roc1b* is required for activation of effector caspase during spermatogenesis [35], yet its role in ovarian development remains unknown. In the ovary of *c587* > *lola*^*12574*^ RNAi line, *Roc1b* expression was upregulated, which may induce ovarian apoptosis. To verify whether *Roc1b* is responsible for the defects in ovarian morphogenesis of the *c587* > *lola*^*12574*^ RNAi line, *Roc1b* was overexpressed in the ovary driven by *c587-Gal4*. Surprisingly, overexpression of *Roc1b* caused defects in ovarian morphogenesis, such as egg chamber fusion and DNA fragmentation in smaller ovaries (Fig. 7A (b, c and c’)), a phenotype similar to that in the *c587* > *lola*^*12574*^ RNAi line. Moreover, knockdown expression of *Roc1b* in the ovary of *c587* > *lola*^*12574*^ RNAi line can obviously rescue the female fertility and the phenotype of lacking ovarioles (Fig. 7A (d), B), and only 14.3% of ovaries (*n* = 49) from the *c587* > *lola;Roc1b* adults showed DNA fragmentation during mid-oogenesis compared to 43% of ovaries from the *c587* > *lola*^*12574*^ RNAi adults. For *c587-Gal4* titration control, we knocked down expression of *lola* in combination with a *UAS-GFP* RNAi and found that there was no difference in the female fertility between the *c587* > *lola;GFP* line and the *c587* > *lola*^*12574*^ RNAi line (Fig. 7B). We also found that in the *c587* > *lola;GFP* females, 36.7% of the ovaries had one ovary with significantly smaller size (Fig. 7A (e)) and 26.8% of the ovaries had a pair of smaller ovaries (Fig. 7A (f)), which were similar to the phenotypes of ovaries from the *c587* > *lola*^*12574*^ RNAi (Fig. 3A (b–d)). Moreover, we observed DNA fragmentation in 80.8% of the smaller ovaries (*n* = 26) from the *c587* > *lola;GFP* females. These results ruled out the possibility that the phenotype of *lola* RNAi rescued by *Roc1b* RNAi was due to Gal4 titration. In addition, knockdown expression of *Roc1b* alone (Fig. 7C) did not have an effect on female fertility and ovarian morphogenesis (Fig. 7A (g), B). Together, these results indicated that *lola* controls ovarian development by suppressing *Roc1b* expression.

## Discussion

*Drosophila* ovary is a model system for understanding the stem cell niche, yet the genetic mechanisms underlying establishment of the niche remain largely unknown. To date, only three transcription factors, Bab, Lmx1a and En/Inv, have been identified as essential factors for the formation or proper stacking of TFs [18, 25, 26]. In this study, we characterized the function of Lola in regulation of ovary development in *Drosophila*. By using two independent *lola* RNAi lines (*lola*^*12574*^ and *lola*^*12052R-1*^) driven by somatic drivers *c587-Gal4* and *Tj-Gal4*, we showed that *lola* is required for the formation of TFs at the time when ovarian stem cell niche is established. It has been shown that Bab is involved in the formation of TFs [18, 22]. Similar to Bab protein, Lola also contains the BTB domain. Both Bab and Lola have been shown to be involved in the formation of TF. Thus, we speculate that BTB transcription factors such as Lola and Bab may be essential for the formation of TFs.

We observed that ovarian atrophy occurred in the *lola* RNAi line when niches were not established normally, a result consistent with previous report that niche dysfunction may lead to tissue degeneration [37]. It has been reported that loss of *lola-K* resulted in blocking the developmental PCD in the late-oogenesis and disrupted the induced PCD during mid-oogenesis in response to starvation [12]. We showed that *lola* is related to adult ovarian apoptosis. But *lola*-regulated apoptosis was not due to starvation since all flies were under well-fed conditions. Also, ovarian apoptosis in *lola*^*12574*^ RNAi line occurred at an earlier time (mid stage) rather than at the late stage of oogenesis and *Roc1b* RNAi partly rescued this phenotype, suggesting that *lola* control ovarian apoptosis through regulating expression of *Roc1b* and other genes. Given that *c587* is expressed in escort cells and early follicle cells of *Drosophila* adults but is not expressed in the mid-oogenesis [19], *lola* is acting non-autonomously to promote egg chamber survival.

It has been shown that Lola-F bound to chromosomal kinase JIL-1 in yeast two hybrid assays [2]. Loss of function in *JIL-1* affected female fertility and resulted in smaller ovaries [38], a morphology similar to that of *lola* RNAi line. However, expression of *JIL-1* in the ovary of *lola*^*12574*^ RNAi line did not change significantly. Further study is required to determine which Lola isoform(s) plays a role in ovarian development.

Using RNA-seq analysis, we identified 433 differentially expressed genes (DEGs) in the ovary of *lola*^*12574*^ RNAi line, with 152 DEGs upregulated and 281 DEGs downregulated. Among these DEGs, we selected an apoptosis-related gene, *Roc1b*. It has been reported that *Roc1b* activates caspase activity during spermatogenesis [35], but its role in ovary development has not been well understood. We showed that overexpression of *Roc1b* in the ovary caused defects in ovarian morphogenesis. To our knowledge, this is the first report to demonstrate that expression of *Roc1b*, a downstream gene of *lola*, can affect ovarian development. Roc1b homolog RBX1 in human has been reported to play a role in high-grade serous ovarian cancer (HGSOC) in women [39]. Lola is a BTB-ZF (zinc finger) transcription factor that is required for stem cell maintenance in *Drosophila* testis [10]. Strikingly, another BTB-ZF transcription factor Promyelocytic leukemia zinc-finger (Plzf), which has a similar structure to Lola, was found to play a role in the maintenance of spermatogonial stem cells in mouse testis [40, 41]. Our findings together with the published results indicate that the reproductive mechanism is conserved from fruit flies to mammals. Therefore, investigating functions of *lola* in *Drosophila* ovary will lead to better understand the role of *lola* in ovary development from invertebrates to mammals.

The establishment of the stem cell niche is regulated by several signaling pathways, including target of rapamycin, insulin, ecdysone, Hippo, and activin signaling pathways [42–45]. It remains unclear how *lola* plays a role in the establishment of stem cell niche, whether through one or more of the above-mentioned signaling pathways. Future studies will focus on this subject.

## Materials and methods

### *Drosophila* lines and experimental conditions

*Lola*^*12574*^ and *lola*^*12052R-1*^ RNAi lines targeting the common BTB domain were obtained from the Vienna *Drosophila* Resource Center (VDRC 12574) and Fly Stocks of the National Institute of Genetics (NIG 12052 R-1), respectively. *UAS-Roc1b* (#34048) and *Roc1b* RNAi (#31067) lines were obtained from Bloomington *Drosophila* Stock Center (BDSC). *UAS-GFP* RNAi (#THJ0356) line was obtained from TsingHua Fly Center. The *c587-Gal4* line was kindly provided by Professor Ting Xie at the Stowers Institute for Medical Research, Kansas City, MO, USA, and *Tj-Gal4* line was kindly provided by Professor Lei Zhang at the Shanghai Institute of Biochemistry and Cell Biology, Chinese Academy of Sciences, Shanghai, China. Wild-type *w*^*1118*^ was maintained in the laboratory. *c587* > *w*^*1118*^ and *Tj* > *w*^*1118*^ (control) lines were obtained by crossing virgin female *c587-Gal4* and *Tj-Gal4*, respectively, with male *w*^*1118*^. All the fly lines were reared on standard cornmeal/molasses diet with p-hydroxybenzoic acid methylester as a mold inhibitor [46].

For analysis of the effect of *lola* in the adult stage, flies reared at 25 °C (from eggs to adults) were used for crosses, and parents were removed 24 h later. Then eggs were collected and kept at 18 °C until pupal stage, pupae were switched to 25 °C until adults, and ovaries were dissected from 3 to 4 days old control and *lola* RNAi adults. Flies in all other experiments were reared at 25 °C from eggs to adults.

### Fertility test

For female fertility assays, 10 1-day-old females and 15 males were placed in bottles with egg collection plates containing grape juice, sucrose and agar (10% grape juice, 2.2% sucrose, 1.4% agar, and 1% ethyl acetate) with wet yeast. Egg collection plates were changed every 24 h. Fertility is reported as eggs laid per female per day [46].

### Immunostaining and TUNEL assays

Ovaries of WPP stage were selected from strictly white pupae. Adult ovaries were taken from day 3 females. All ovaries were dissected in phosphate buffered saline (PBS), fixed for 10 min in 4% paraformaldehyde at room temperature, washed three times in PBS containing 0.1% Triton X-100 (PBT) (each for 15 min), and blocked in 5% normal goat serum for 1 h. Ovaries were incubated with primary antibodies diluted in PBT overnight at 4 °C. Then, ovaries were washed three times in PBT and incubated with secondary antibodies at room temperature for 1 h. After washing three times again with 0.1% PBT, ovaries were finally mounted in PBS/glycerol medium with DAPI as described previously [47]. The primary antibodies used were rabbit anti-Lola (a gift from Professor Lei Zhang) (1:500) and mouse anti-En (4D9, DSHB, IA, USA) (1:50). The secondary antibodies were DyLight 594 conjugated goat anti rabbit (#A23440, Abbkine, Beijing, China) (1:1000) and DyLight 488 conjugated goat anti mouse (#A23210, Abbkine, Beijing, China) (1:1000). TUNEL assay was performed using the DeadEnd Fluorometric TUNEL kit (Promega, WI, USA) following the manufacturer’s instructions. All images were collected on a Zeiss LSM 710 Confocal Microscope (Thornwood, NY, USA).

### RNA sequencing

We used 3-day-old *c587* > *lola*^*12574*^ RNAi and *c587* > *w*^*1118*^ (control) flies in this study. Ovaries from 80 *c587* > *lola*^*12574*^ RNAi females and 40 *c587* > *w*^*1118*^ females were dissected in PBS. Total RNA was extracted with Trizol (Invitrogen, CA, USA) following the recommendations of the manufacturer. Total RNA was used in the following procedures: (1) oligo-dT magnetic beads was used to purify mRNA with poly(A) tails; (2) the purified mRNA was fragmented and reverse transcribed to synthesize double-stranded cDNAs (ds-cDNAs) using *EasyScript* RT/RI Enzyme Mix (Transgene, Beijing, China); (3) the ds-cDNAs were subjected to traditional processing that included ligation of indexed Illumina adapters and amplification using limited-cycle PCR. (4) the ds-cDNAs PCR products were heat-denatured to form single strands, and then a bridge primer was used to circularize the single-stranded DNA to obtain a single-stranded circular DNA library; (5) sequencing was performed at the Beijing Genomics Institute (BGI, Shenzhen, China) using an Illumina HiSeq 2000 (Illumina, San Diego, CA, USA).

### Bioinformatics analysis of RNA-seq data

The transcript abundances in this study were determined using Fragments Per Kilobase per Million mapped reads (FPKM) values. Differentially expressed genes (DEGs) were identified based on a log_2_ fold-change > 1.3 (or log_2_ fold-change < −1.3) and a *p* < 0.05 with four biological replicates. Volcano plot was obtained to display visually the distribution of −log_10_ *p*-values and fold-change values of DEGs between two groups. Gene Ontology (GO) enrichment analysis of DEGs was performed by using the GOseq R package.

### Quantitative real-time PCR (qRT-PCR)

Total RNA was extracted from 3-day-old *c587* > *lola*^*12574*^ RNAi and *c587* > *w*^*1118*^ control female ovaries using Trizol reagent (Invitrogen). The first-strand cDNA was synthesized from 2 μg of total RNA using EasyScript first-strand cDNA synthesis SuperMix (Transgene, Beijing, China). qRT-PCR was performed with a TransStart Tip Green qPCR SuperMix (Transgene). The qRT-PCR experiments were conducted using a CFX connect™ real-time system (BioRad, CA, USA), as described previously [48]. The relative expression of the gene was calibrated against the reference gene *rp49* using 2^-ΔCT^ (ΔCT = CT target gene–CT rp49). The primers used in this study are shown in Supplementary Table S4.

### Statistical analysis

Three biological replicates and three technical replicates for each biological replicate were performed. The significant difference was determined by the student’s t-test or by one-way ANOVA followed by a post hoc Tukey’s honestly significant difference [HSD] test using GraphPad.

## Supplementary information

Supplementary Table legends

Total genes identified by RNA-seq.

Up-regulated Genes

Down-regulated Genes

The primers used in this study.

Reproducibility checklist

Original Data

Original Data

Original Data

Original Data

Original Data

Original Data

Original Data

Original Data

Original Data

Original Data

Original Data

Original Data

Original Data

Original Data

Original Data

Original Data

Original Data

Original Data

Original Data

Original Data

Original Data

Original Data

Original Data

Original Data

Original Data

Original Data

Original Data

Original Data

Original Data

Original Data

Original Data

Original Data

Original Data

Original Data

Original Data

Original Data

Original Data

Original Data

Original Data

Original Data

Original Data

Original Data

Original Data

Original Data

Original Data

Original Data

Original Data

Original Data

Original Data

Original Data

Original Data

Original Data

Original Data

Original Data

Original Data

Original Data

Original Data

Original Data

Original Data

## Data Availability

All datasets generated and analyzed during this study are included in this published article and its Supplementary Information files. Additional data are available from the corresponding author upon reasonable request.
