# Supplementary material for: A dual role of lola in Drosophila ovary development: regulating stem cell niche establishment and repressing apoptosis
Source: Cell Death Dis. 2022 Sep 2;13(9):756. doi: 10.1038/s41419-022-05195-9 (PMC9440207; doi:10.1038/s41419-022-05195-9)
Supplement: Supplementary file 1 — Supplementary Table legends [file 41419_2022_5195_MOESM1_ESM.docx]

**Supplementary Table S1.** **Total genes identified by RNA-seq.**

**Supplementary Table S2. Genes with increased transcript levels in *lola* RNAi ovaries relative to control ovaries.**

**Supplementary Table S3.** **Genes with decreased transcript levels in *lola* RNAi ovaries relative to control ovaries.**

**Supplementary Table S4. The primers used in this study.**
