# Supplementary figures and images for: A dual role of lola in Drosophila ovary development: regulating stem cell niche establishment and repressing apoptosis
Source: Cell Death Dis. 2022 Sep 2;13(9):756. doi: 10.1038/s41419-022-05195-9 (PMC9440207; doi:10.1038/s41419-022-05195-9)

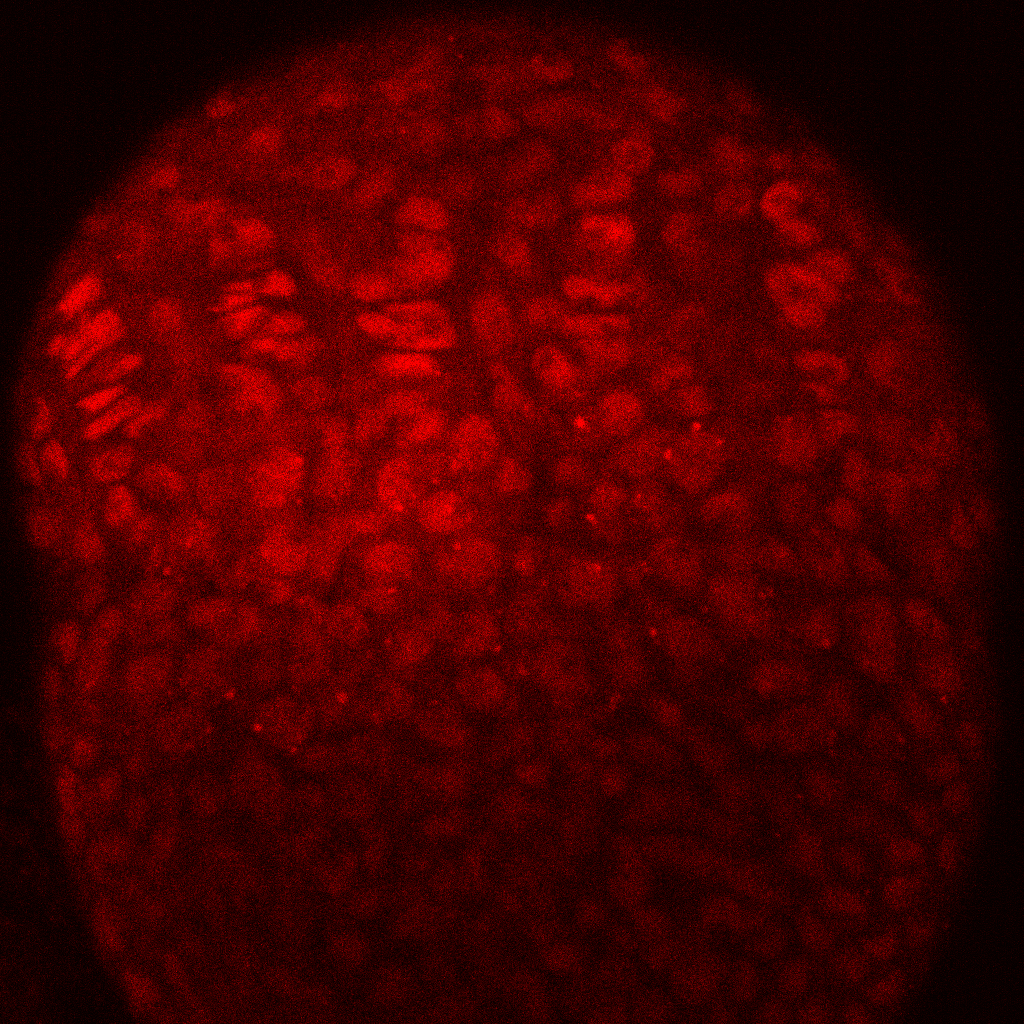

Supplement: Supplementary file 7 — Original Data [file 41419_2022_5195_MOESM7_ESM.tif]

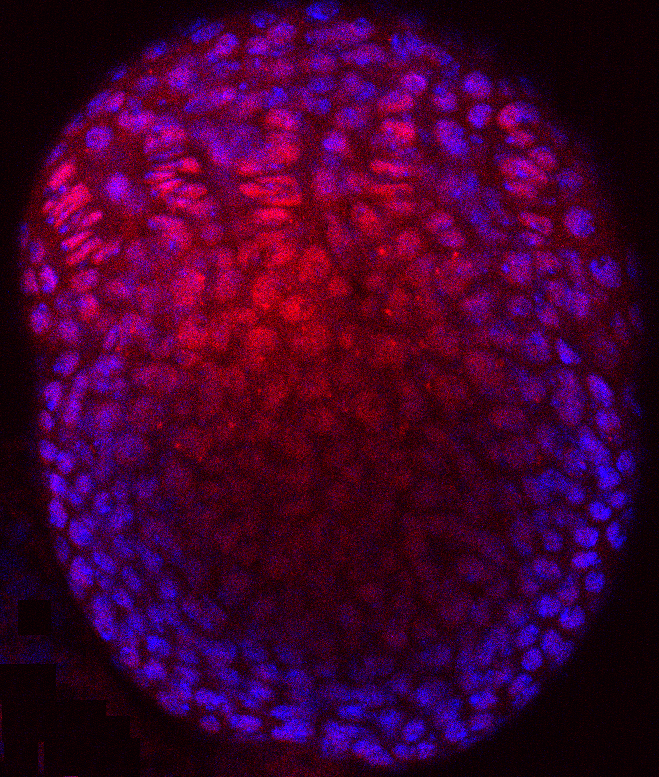

Supplement: Supplementary file 8 — Original Data [file 41419_2022_5195_MOESM8_ESM.tif]

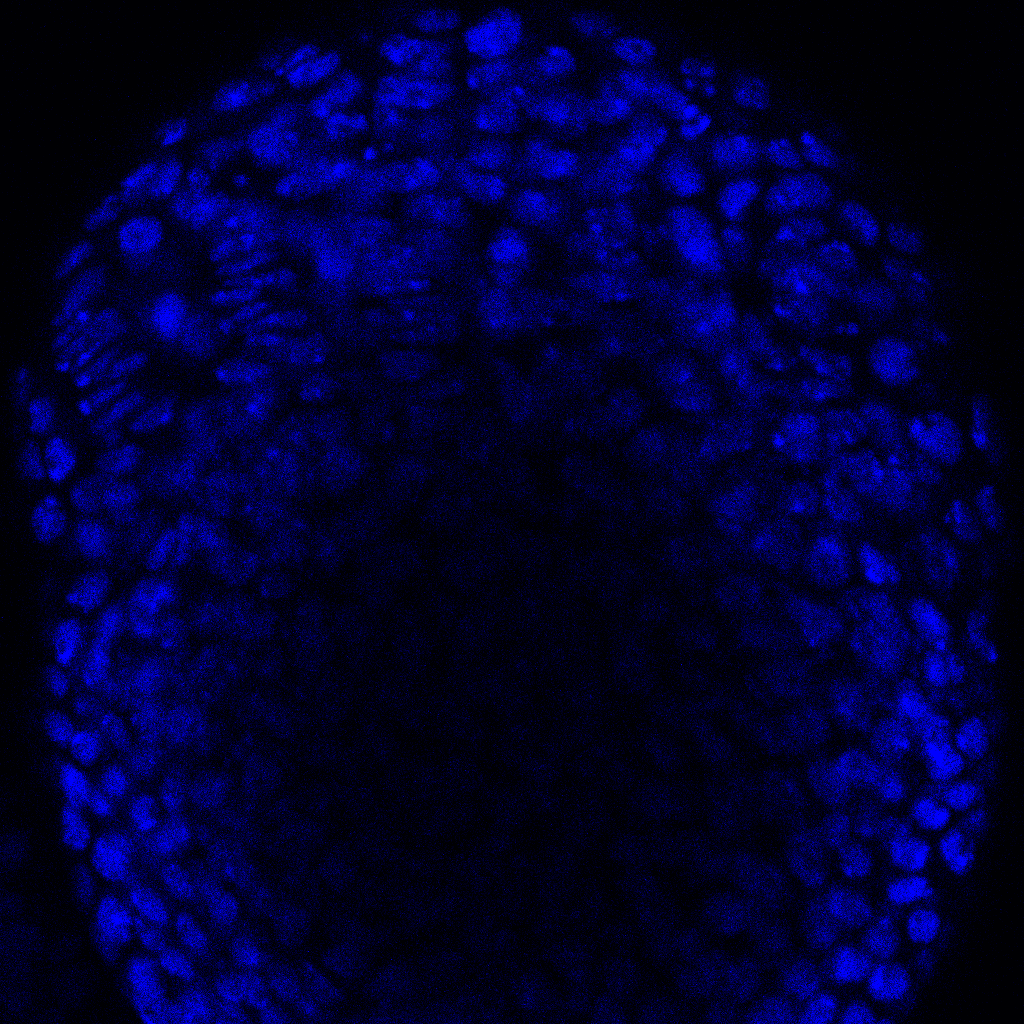

Supplement: Supplementary file 9 — Original Data [file 41419_2022_5195_MOESM9_ESM.tif]

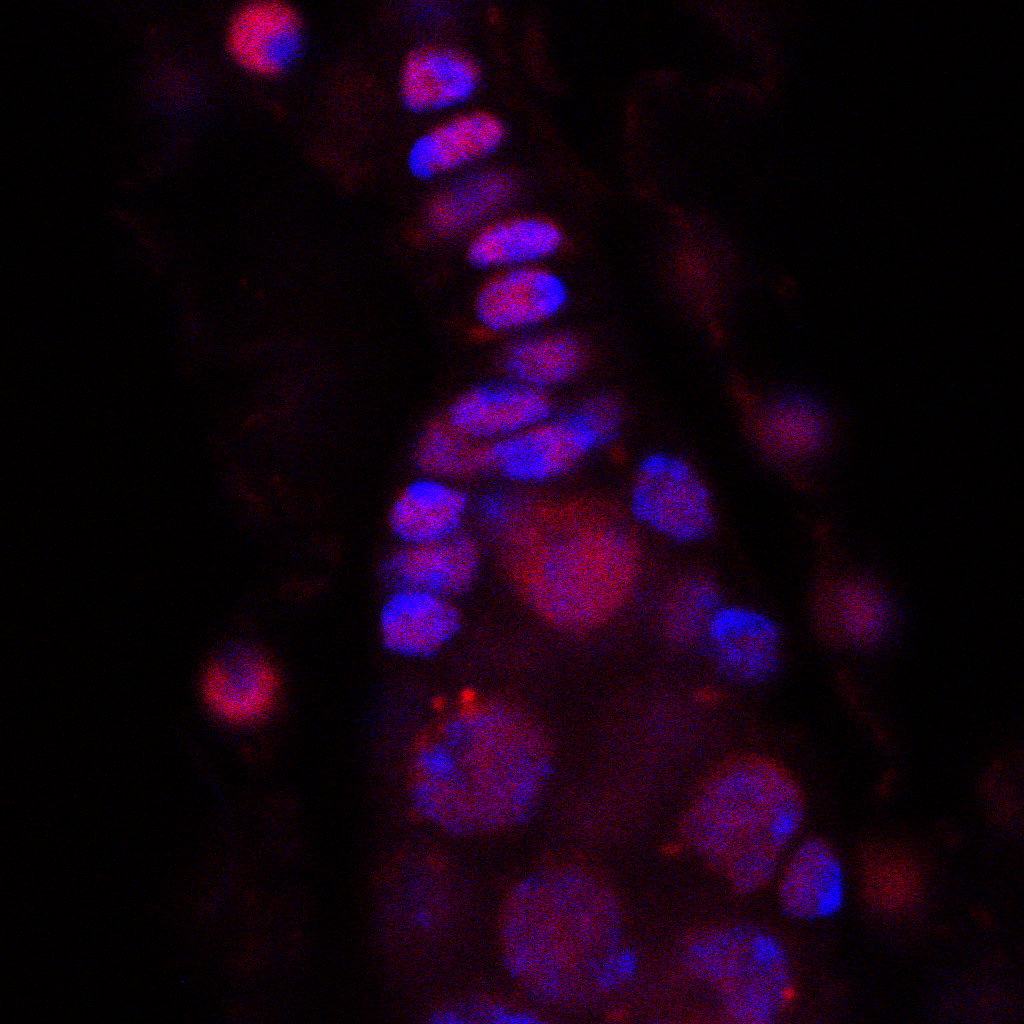

Supplement: Supplementary file 10 — Original Data [file 41419_2022_5195_MOESM10_ESM.tif]

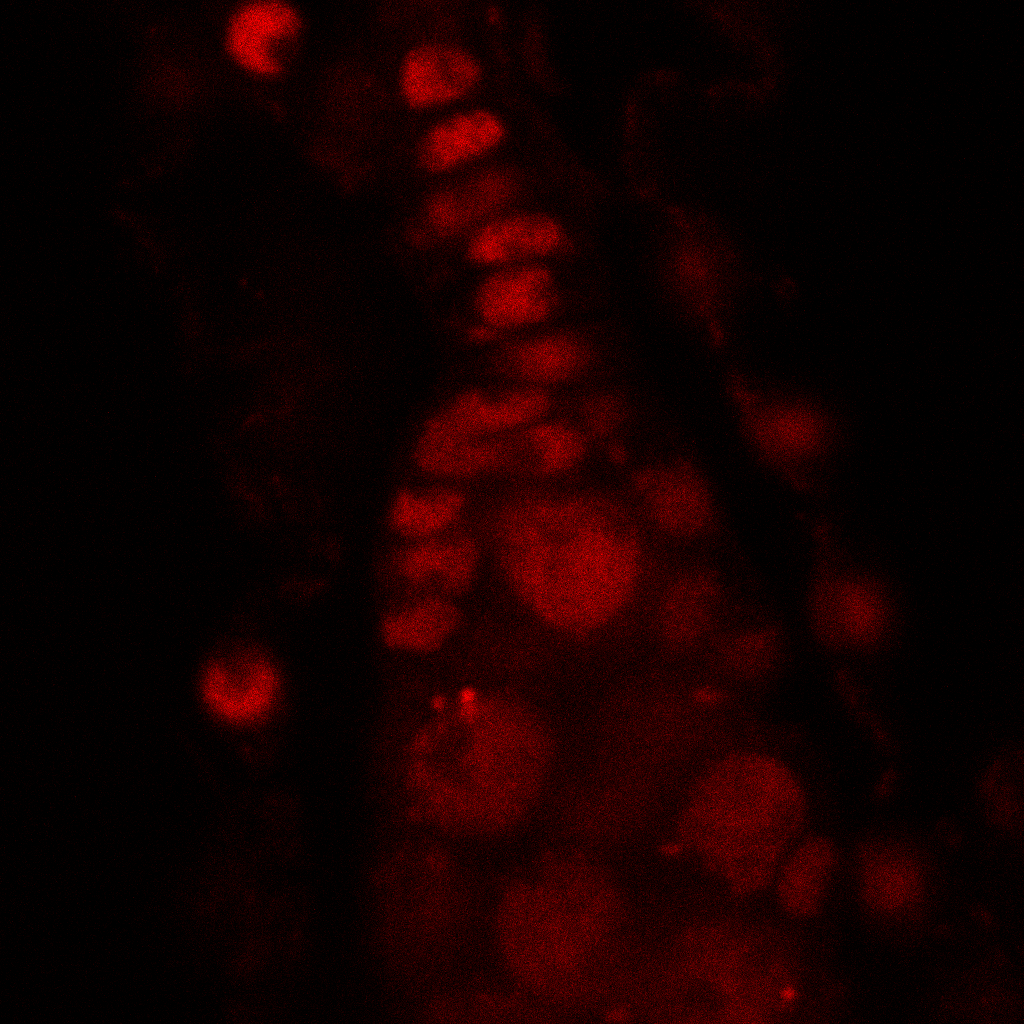

Supplement: Supplementary file 11 — Original Data [file 41419_2022_5195_MOESM11_ESM.tif]

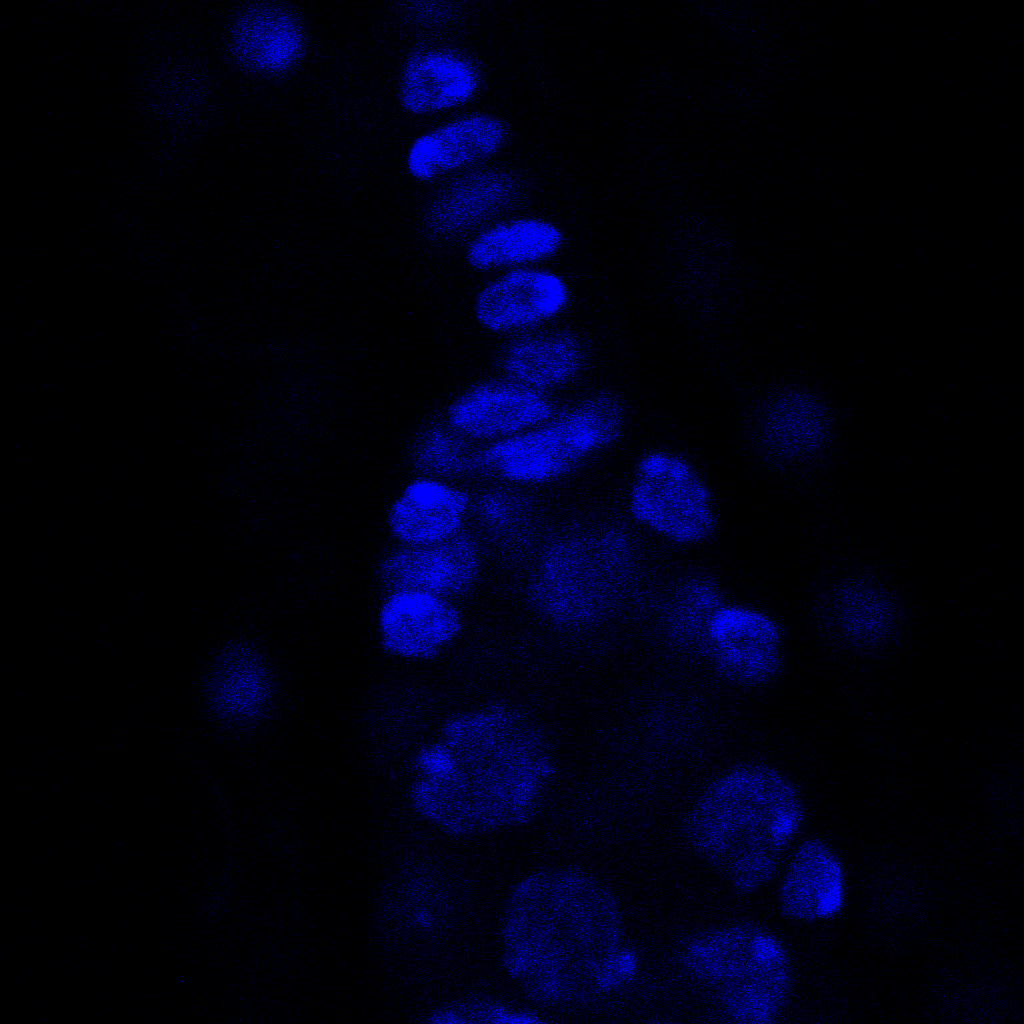

Supplement: Supplementary file 12 — Original Data [file 41419_2022_5195_MOESM12_ESM.tif]

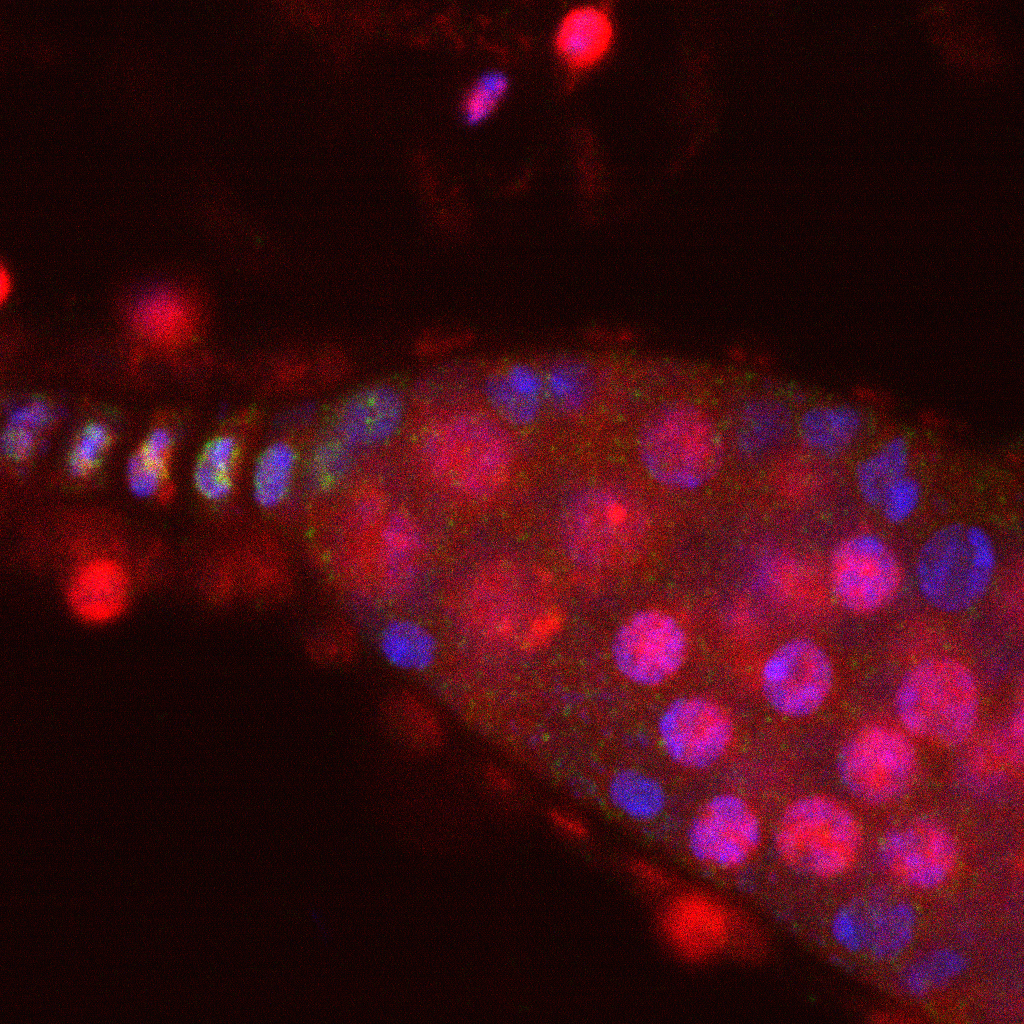

Supplement: Supplementary file 13 — Original Data [file 41419_2022_5195_MOESM13_ESM.tif]

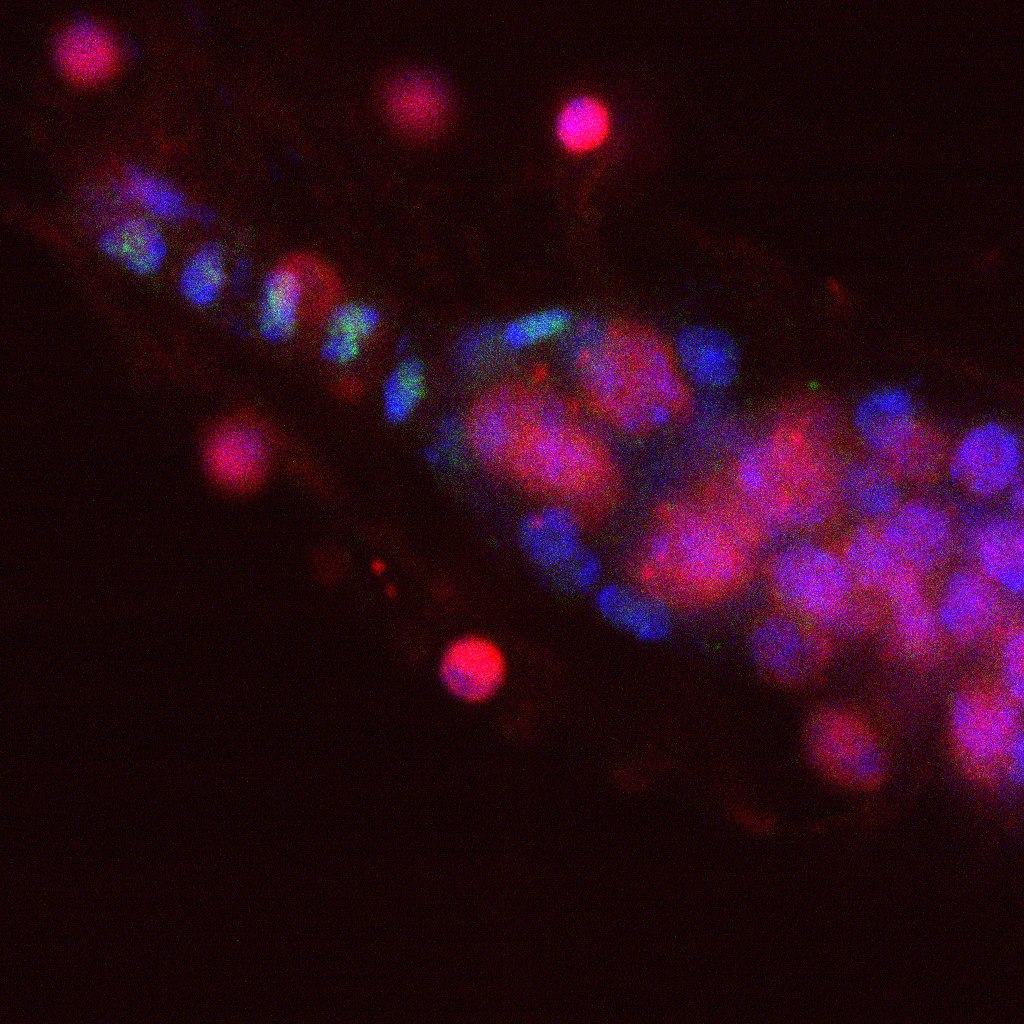

Supplement: Supplementary file 14 — Original Data [file 41419_2022_5195_MOESM14_ESM.tif]

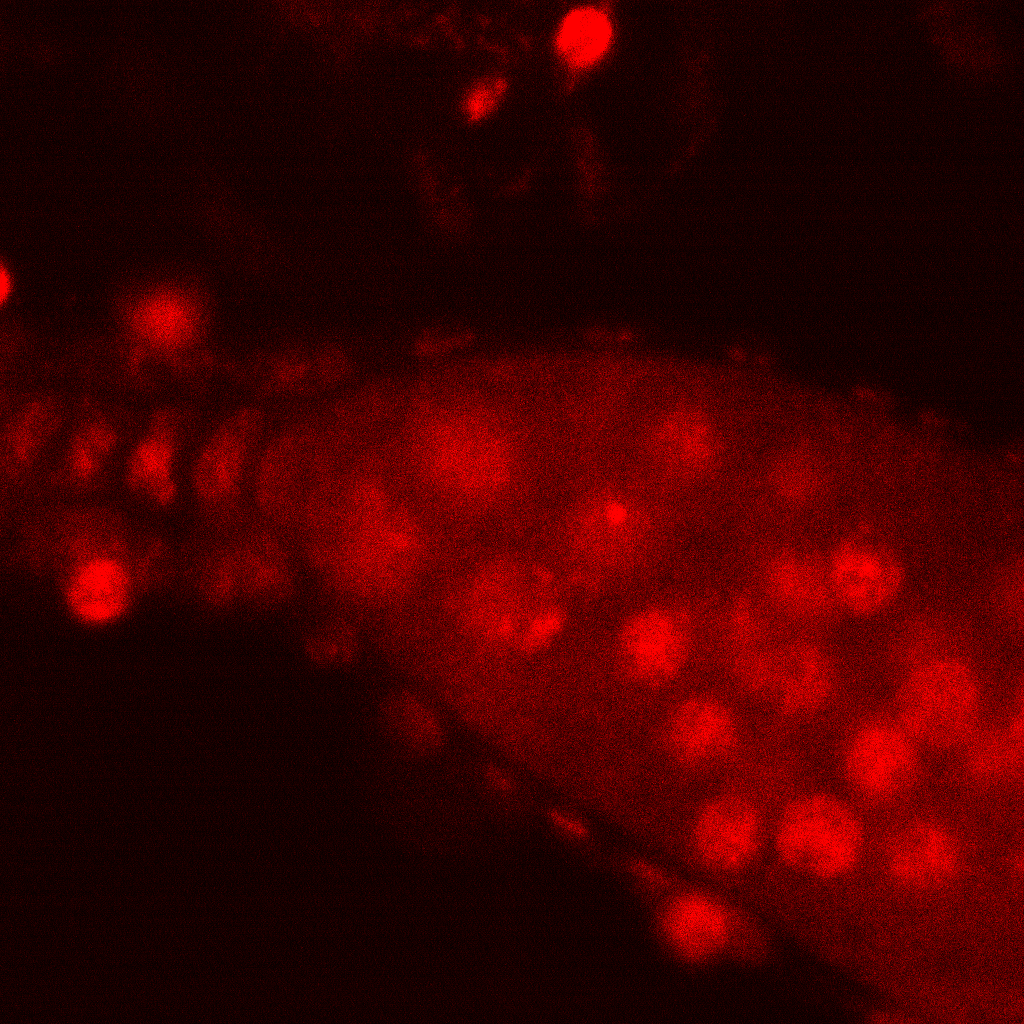

Supplement: Supplementary file 15 — Original Data [file 41419_2022_5195_MOESM15_ESM.tif]

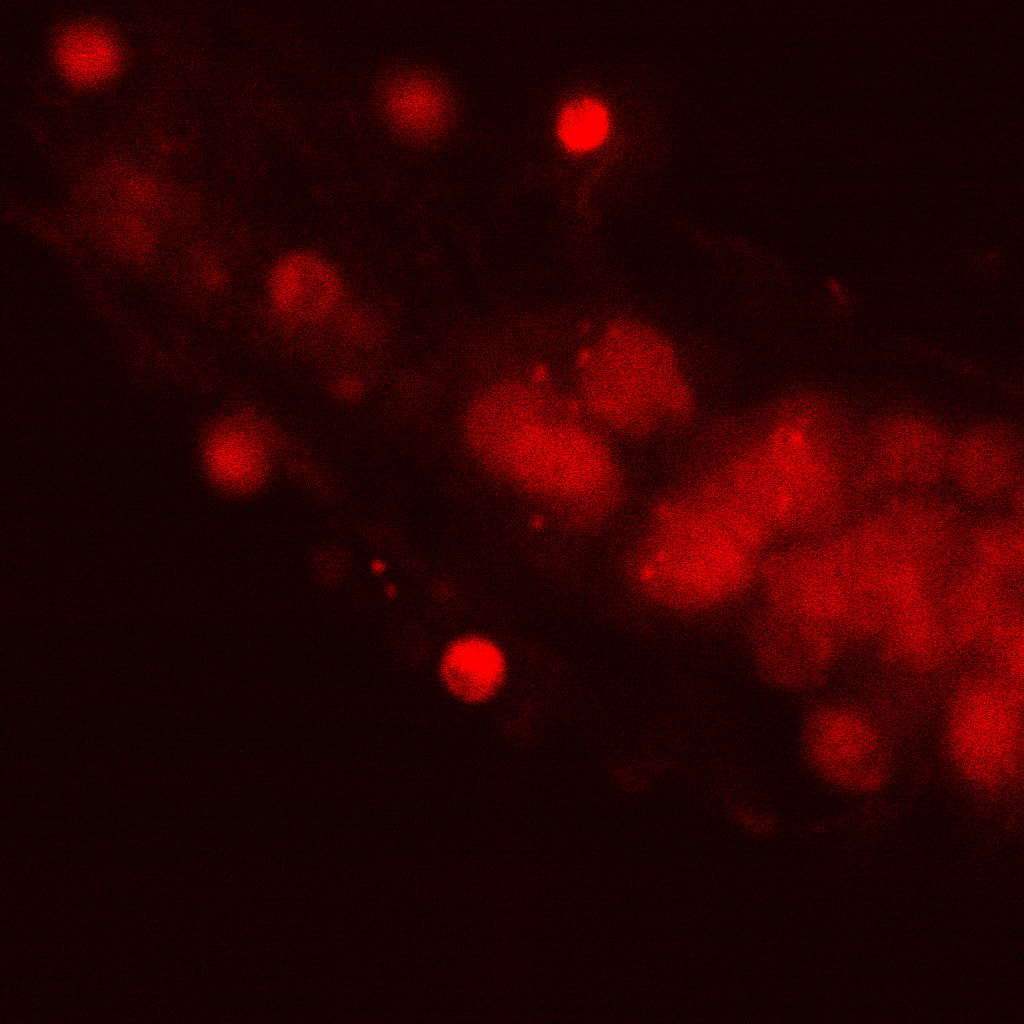

Supplement: Supplementary file 16 — Original Data [file 41419_2022_5195_MOESM16_ESM.tif]

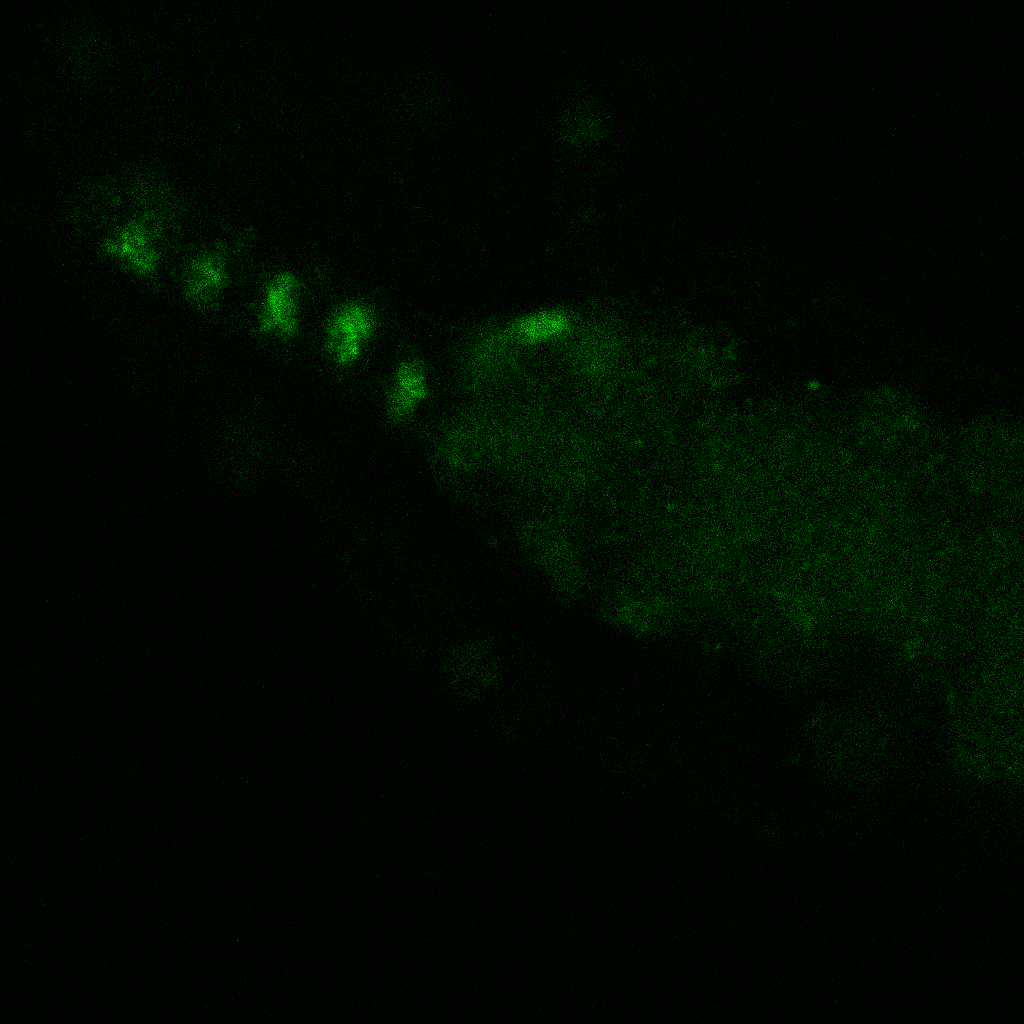

Supplement: Supplementary file 17 — Original Data [file 41419_2022_5195_MOESM17_ESM.tif]

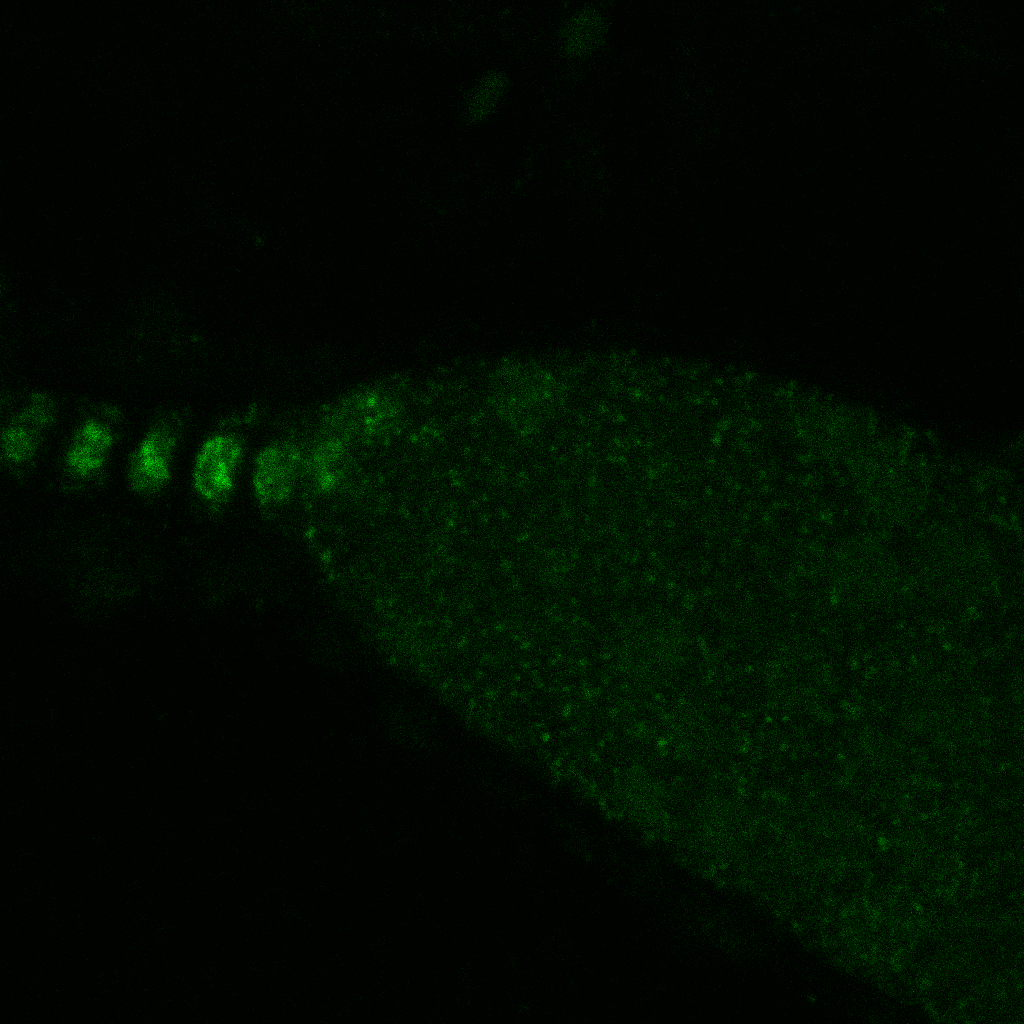

Supplement: Supplementary file 18 — Original Data [file 41419_2022_5195_MOESM18_ESM.tif]

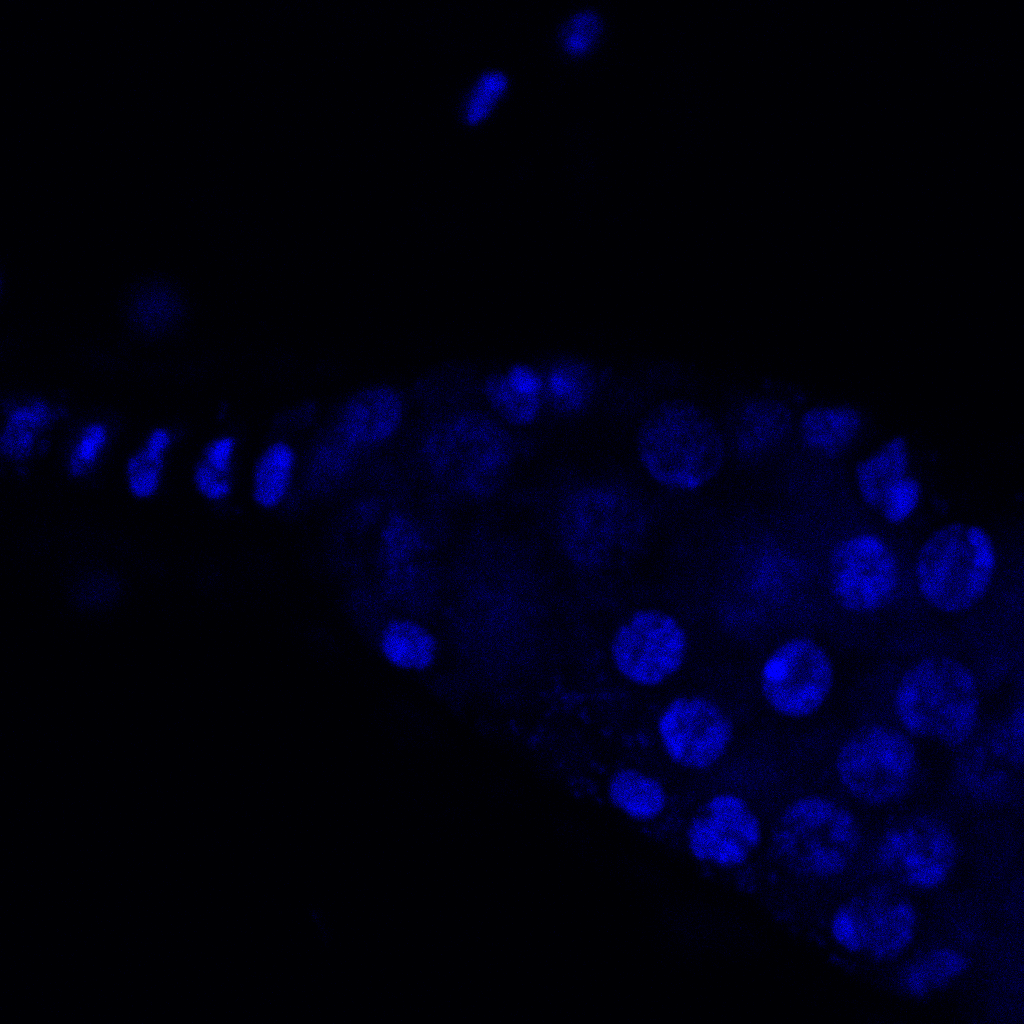

Supplement: Supplementary file 19 — Original Data [file 41419_2022_5195_MOESM19_ESM.tif]

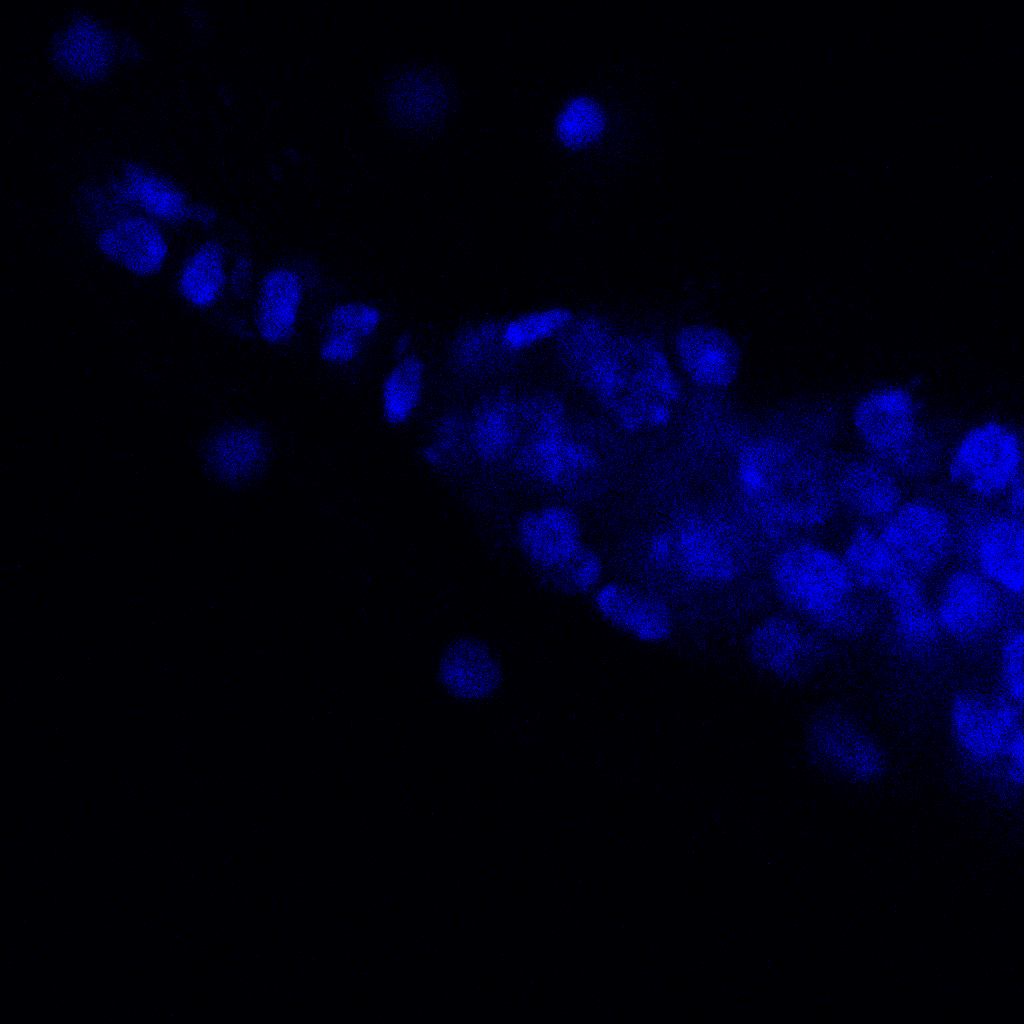

Supplement: Supplementary file 20 — Original Data [file 41419_2022_5195_MOESM20_ESM.tif]

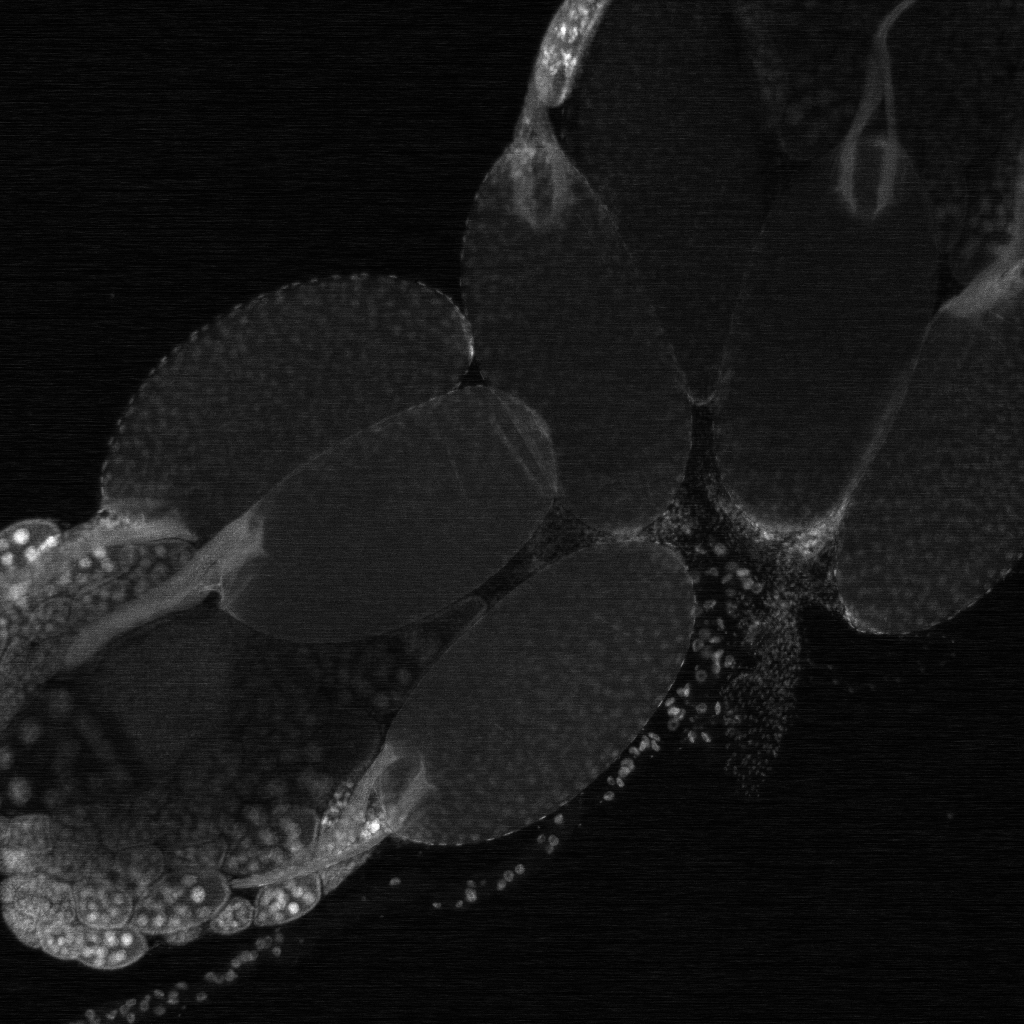

Supplement: Supplementary file 21 — Original Data [file 41419_2022_5195_MOESM21_ESM.tif]

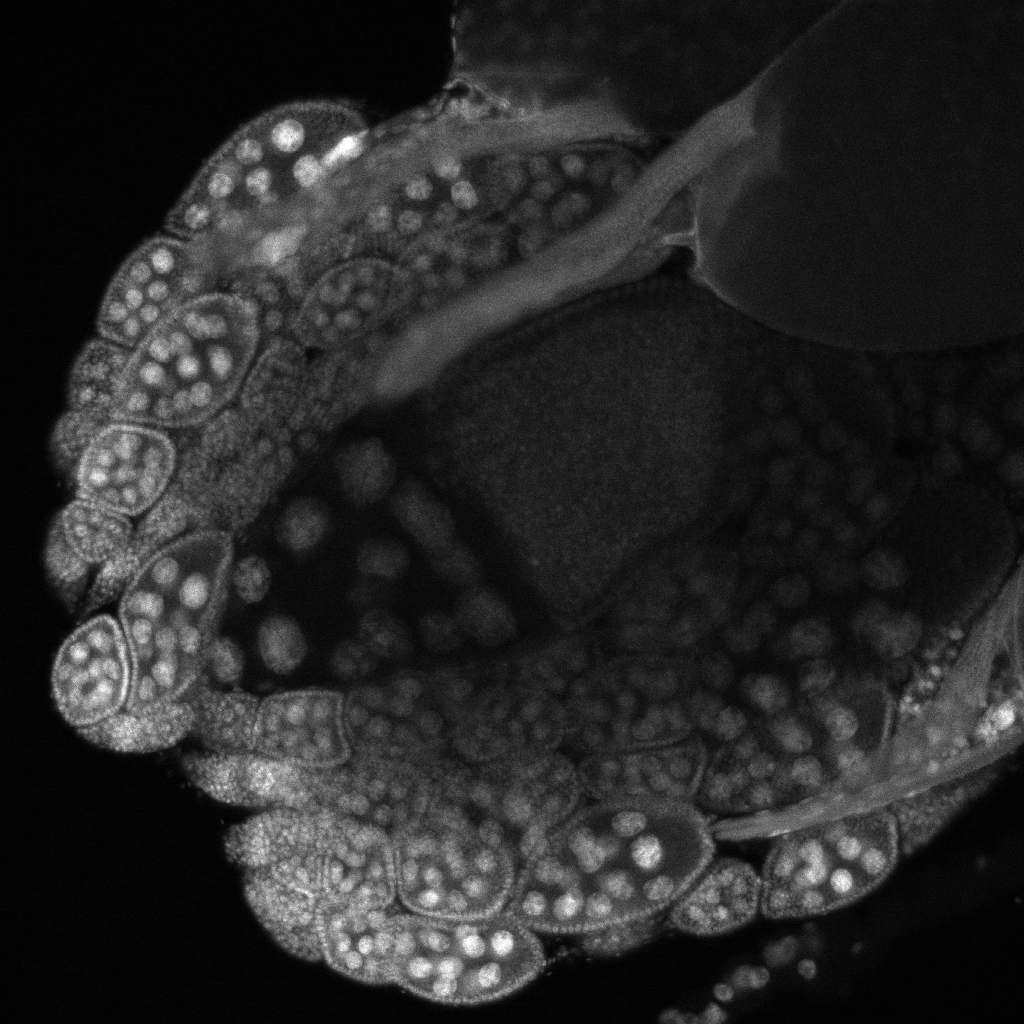

Supplement: Supplementary file 22 — Original Data [file 41419_2022_5195_MOESM22_ESM.tif]

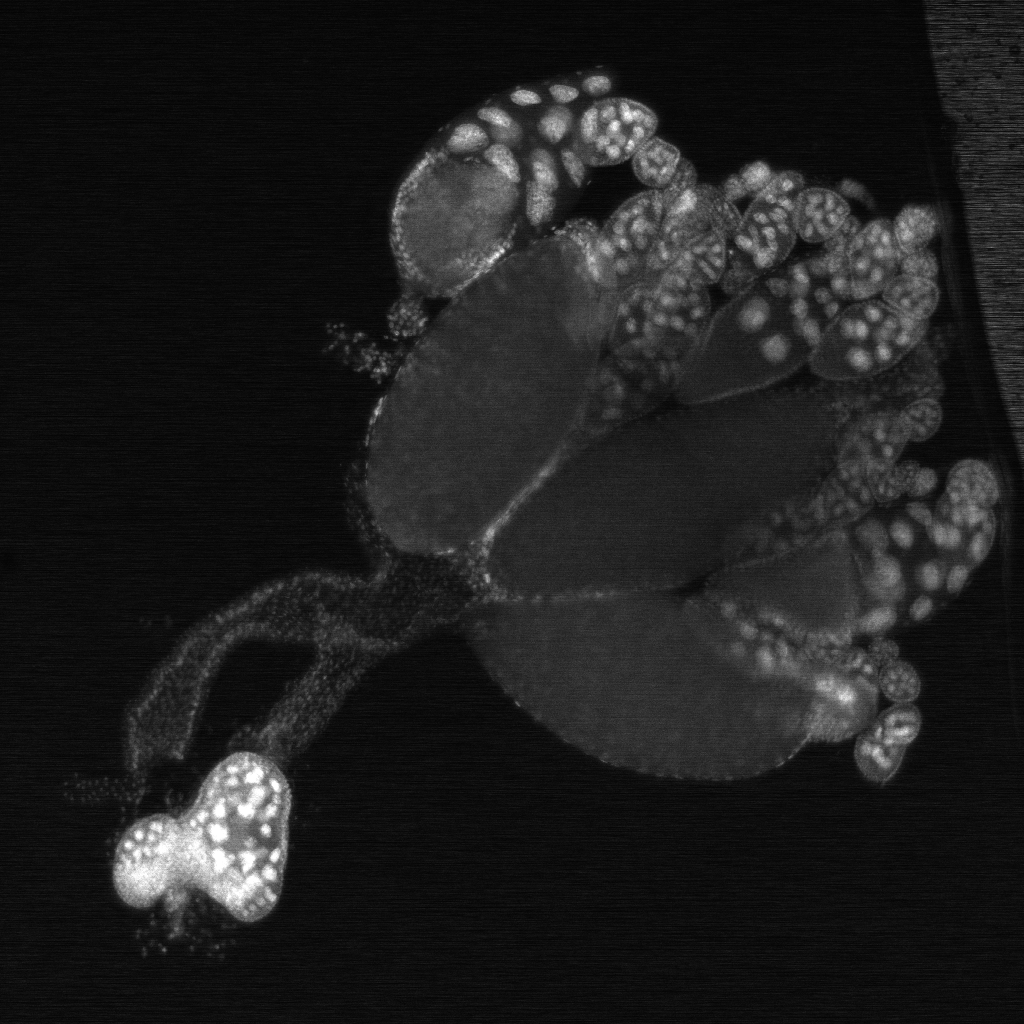

Supplement: Supplementary file 23 — Original Data [file 41419_2022_5195_MOESM23_ESM.tif]

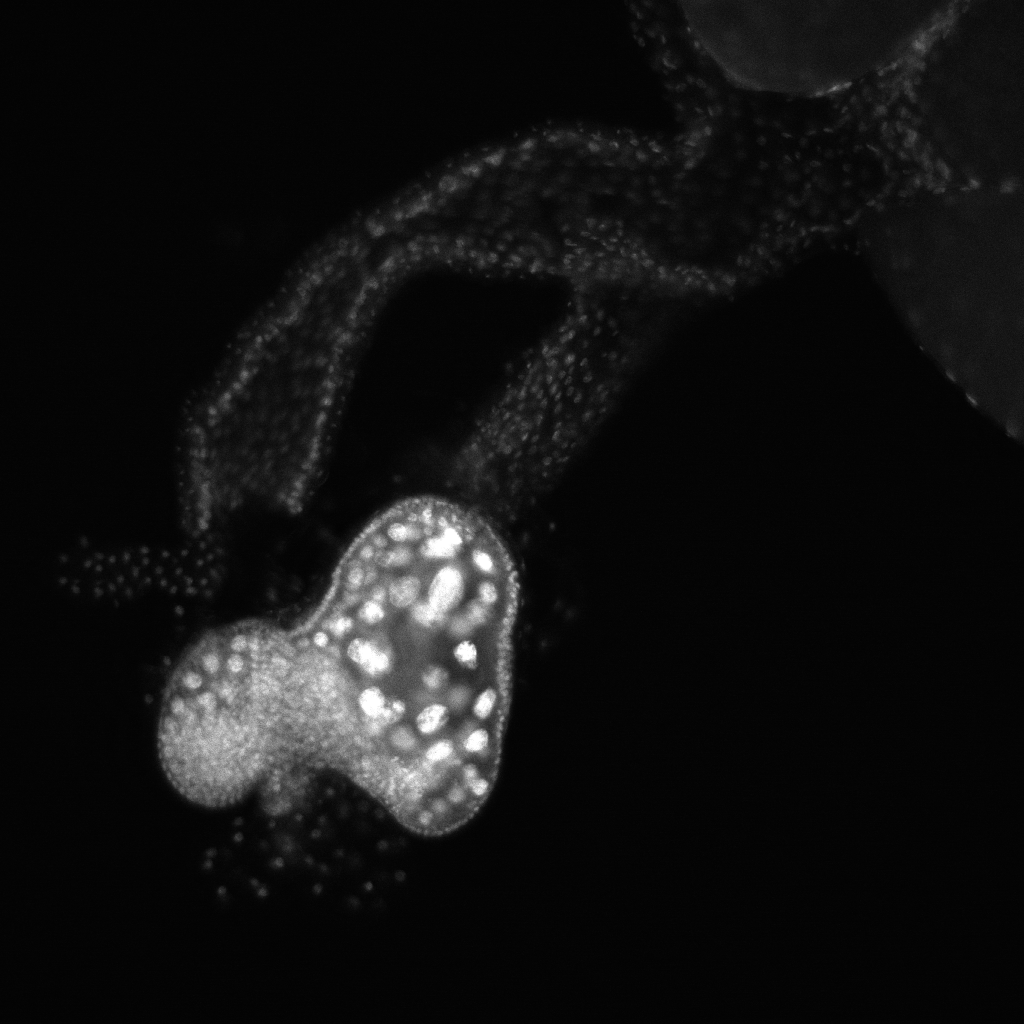

Supplement: Supplementary file 24 — Original Data [file 41419_2022_5195_MOESM24_ESM.tif]

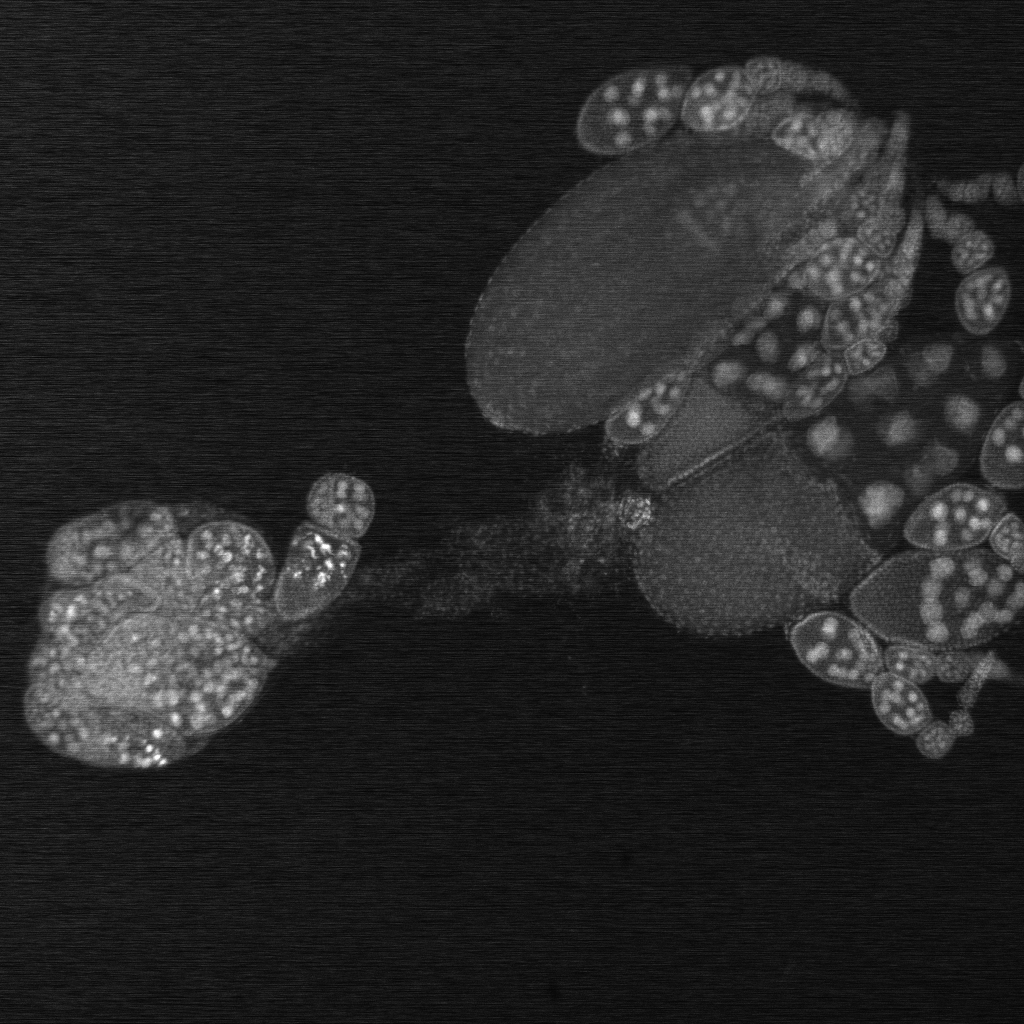

Supplement: Supplementary file 25 — Original Data [file 41419_2022_5195_MOESM25_ESM.tif]

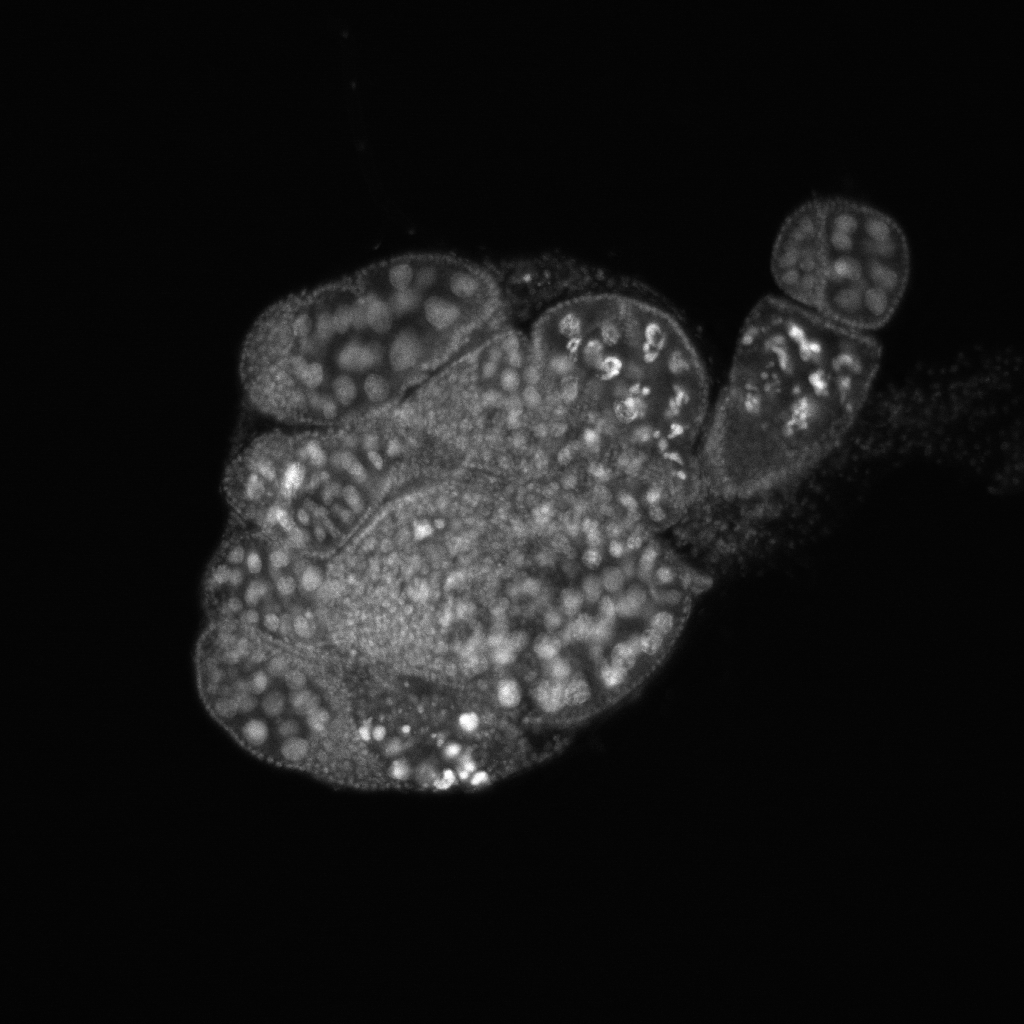

Supplement: Supplementary file 26 — Original Data [file 41419_2022_5195_MOESM26_ESM.tif]

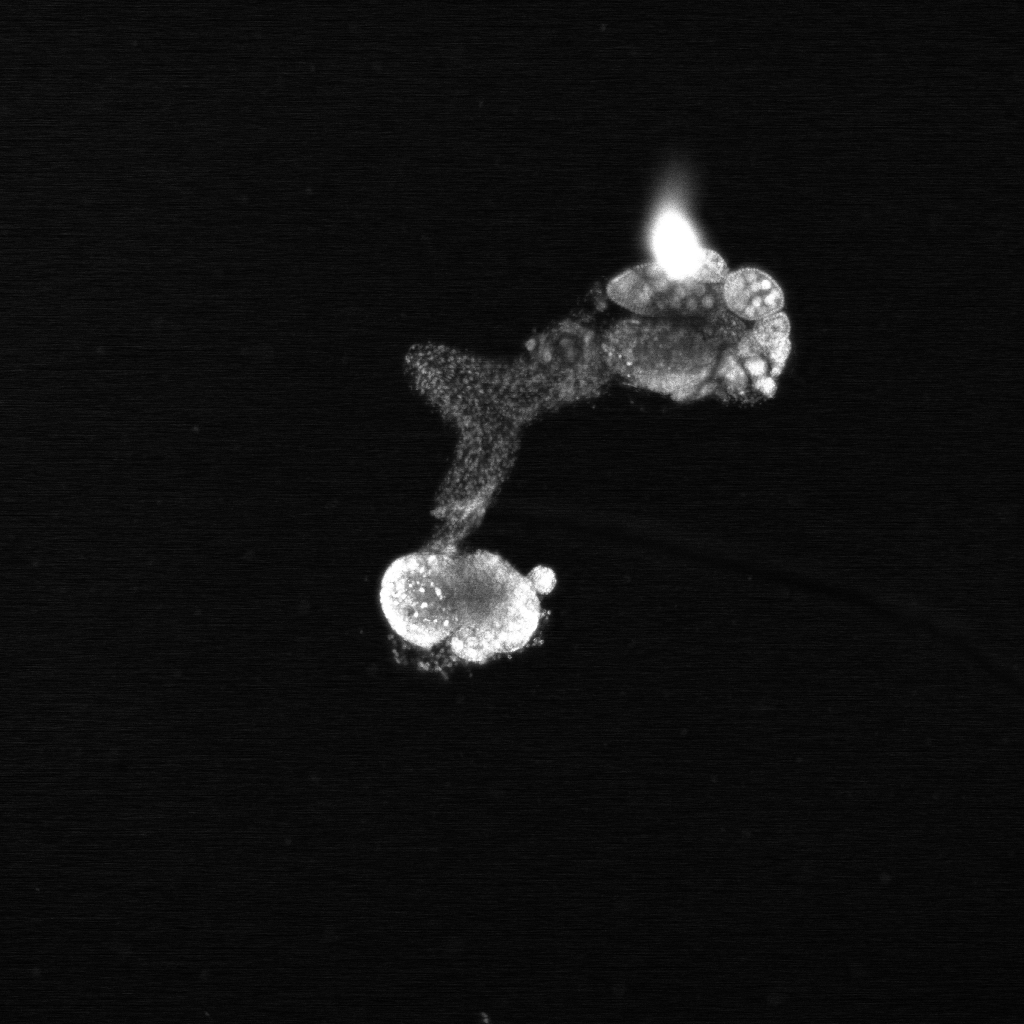

Supplement: Supplementary file 27 — Original Data [file 41419_2022_5195_MOESM27_ESM.tif]

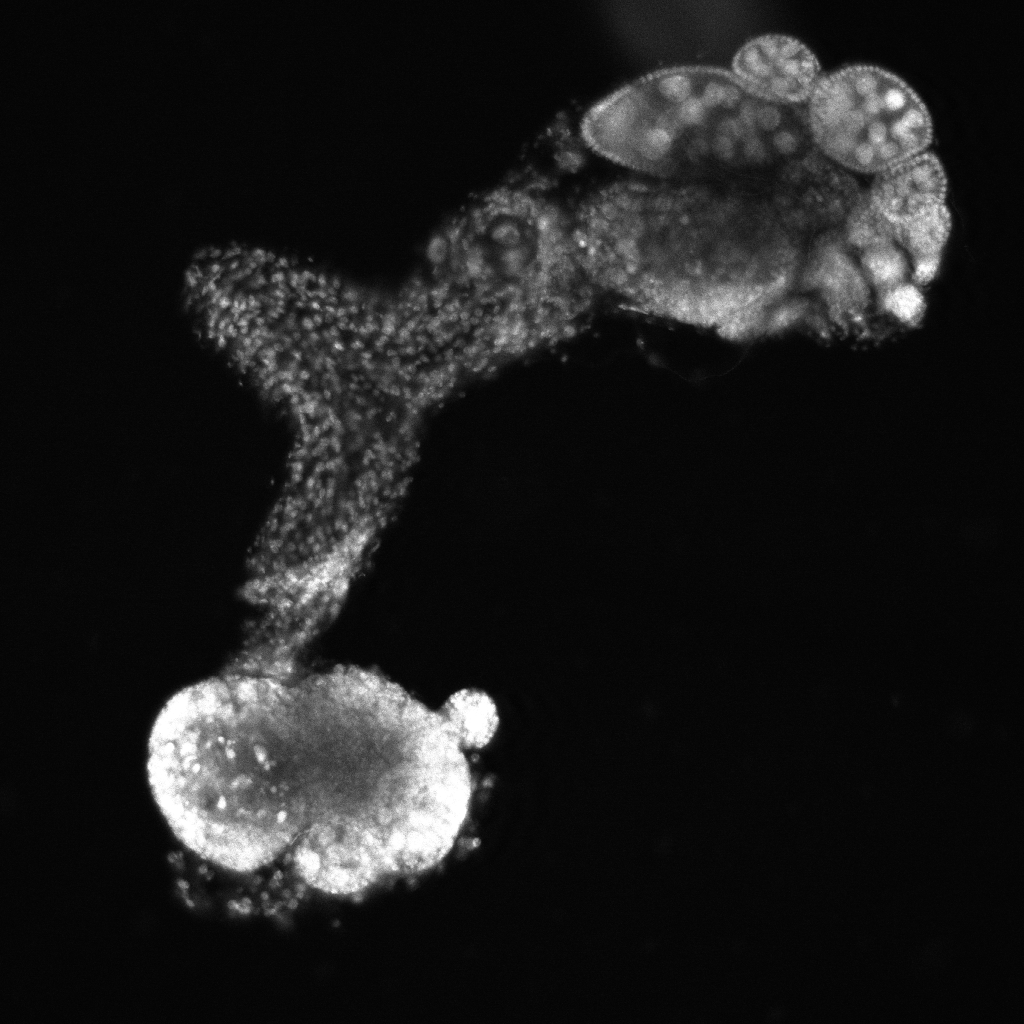

Supplement: Supplementary file 28 — Original Data [file 41419_2022_5195_MOESM28_ESM.tif]

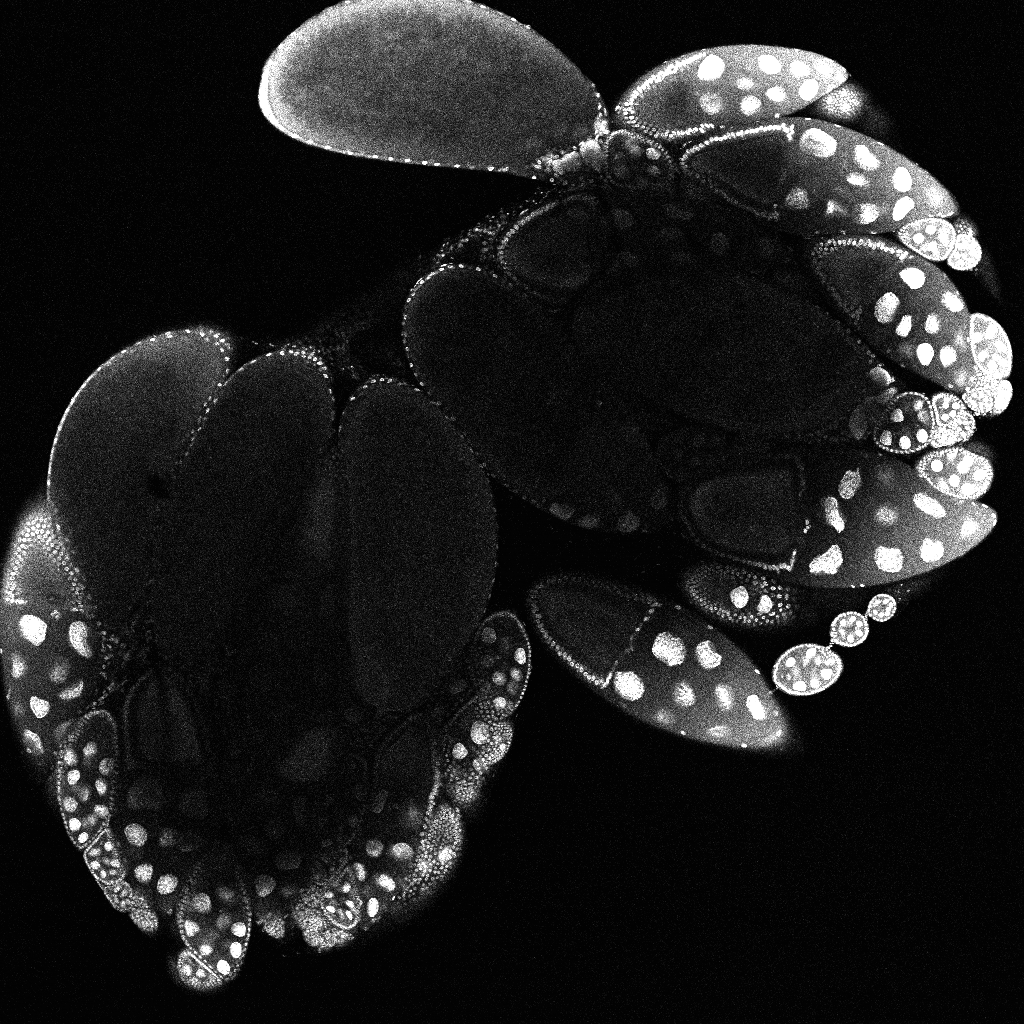

Supplement: Supplementary file 29 — Original Data [file 41419_2022_5195_MOESM29_ESM.tif]

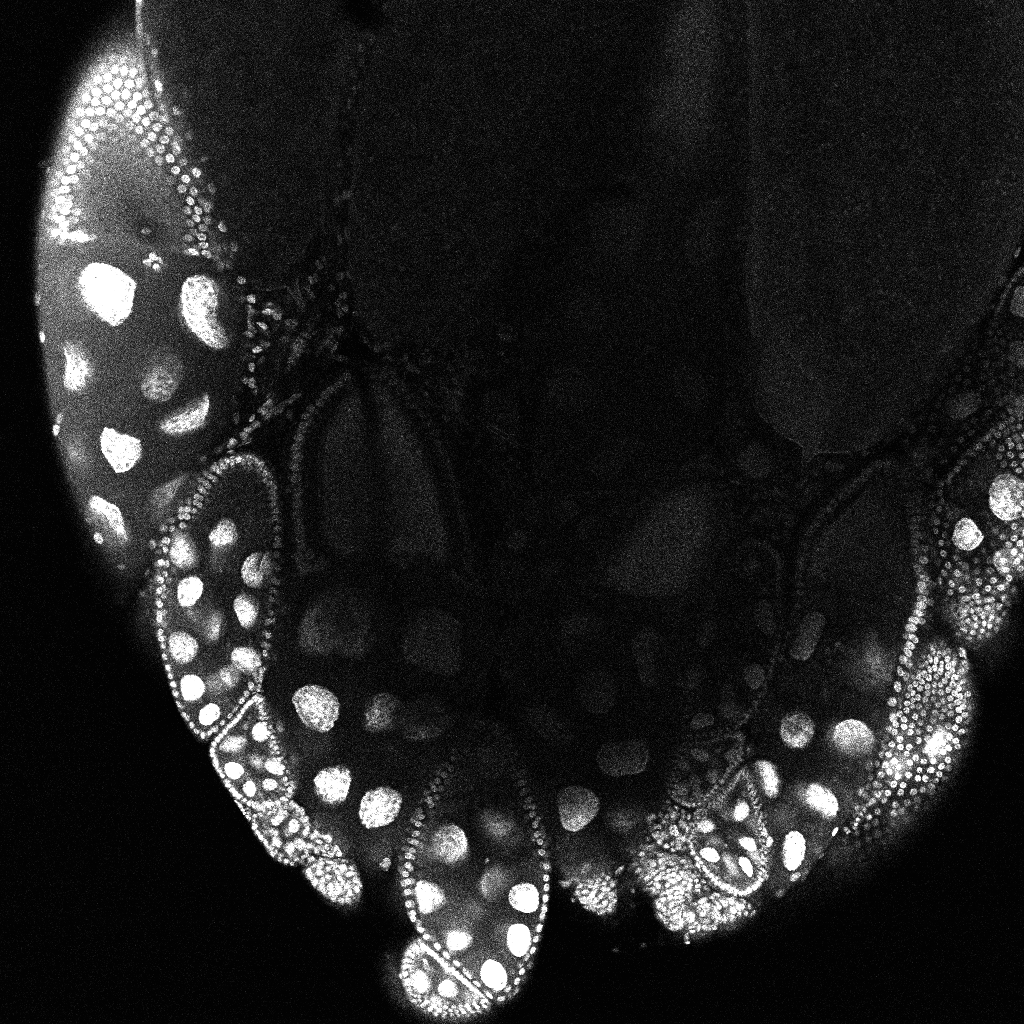

Supplement: Supplementary file 30 — Original Data [file 41419_2022_5195_MOESM30_ESM.tif]

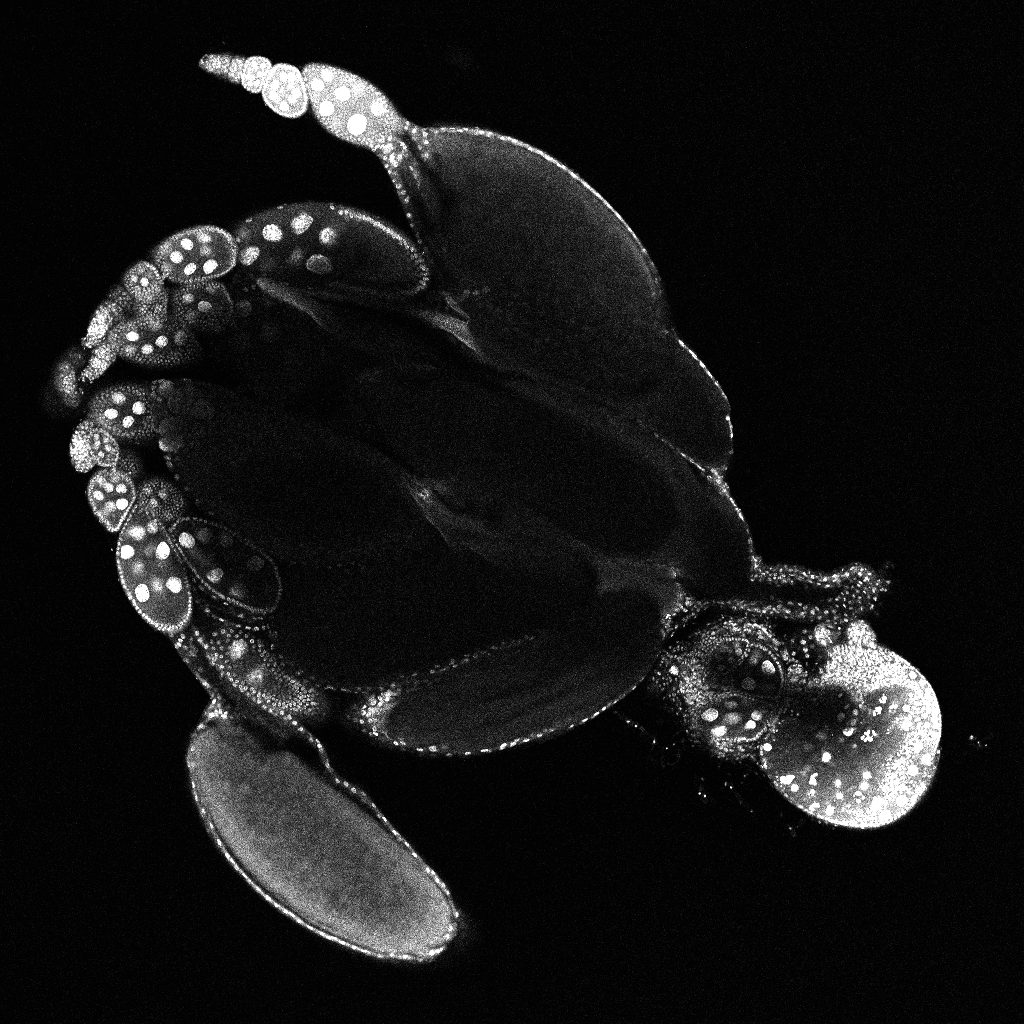

Supplement: Supplementary file 31 — Original Data [file 41419_2022_5195_MOESM31_ESM.tif]

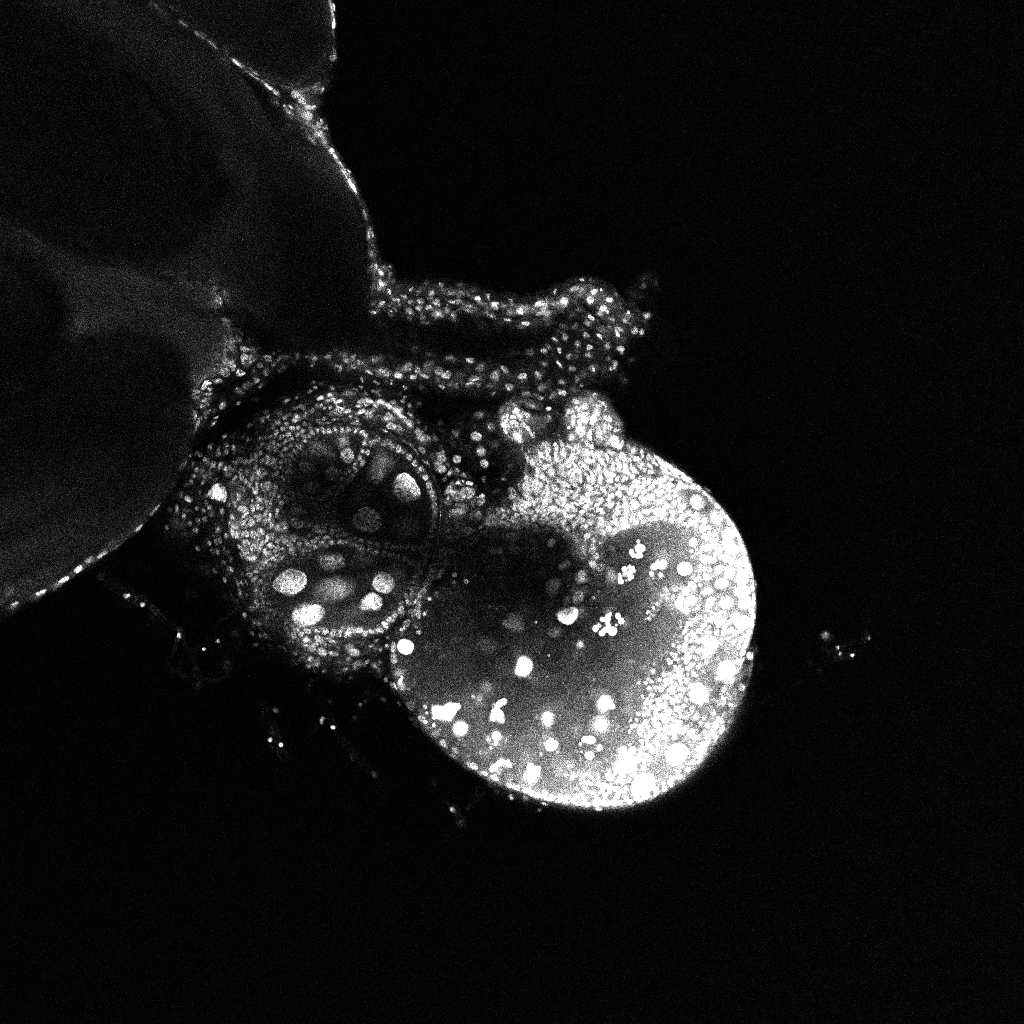

Supplement: Supplementary file 32 — Original Data [file 41419_2022_5195_MOESM32_ESM.tif]

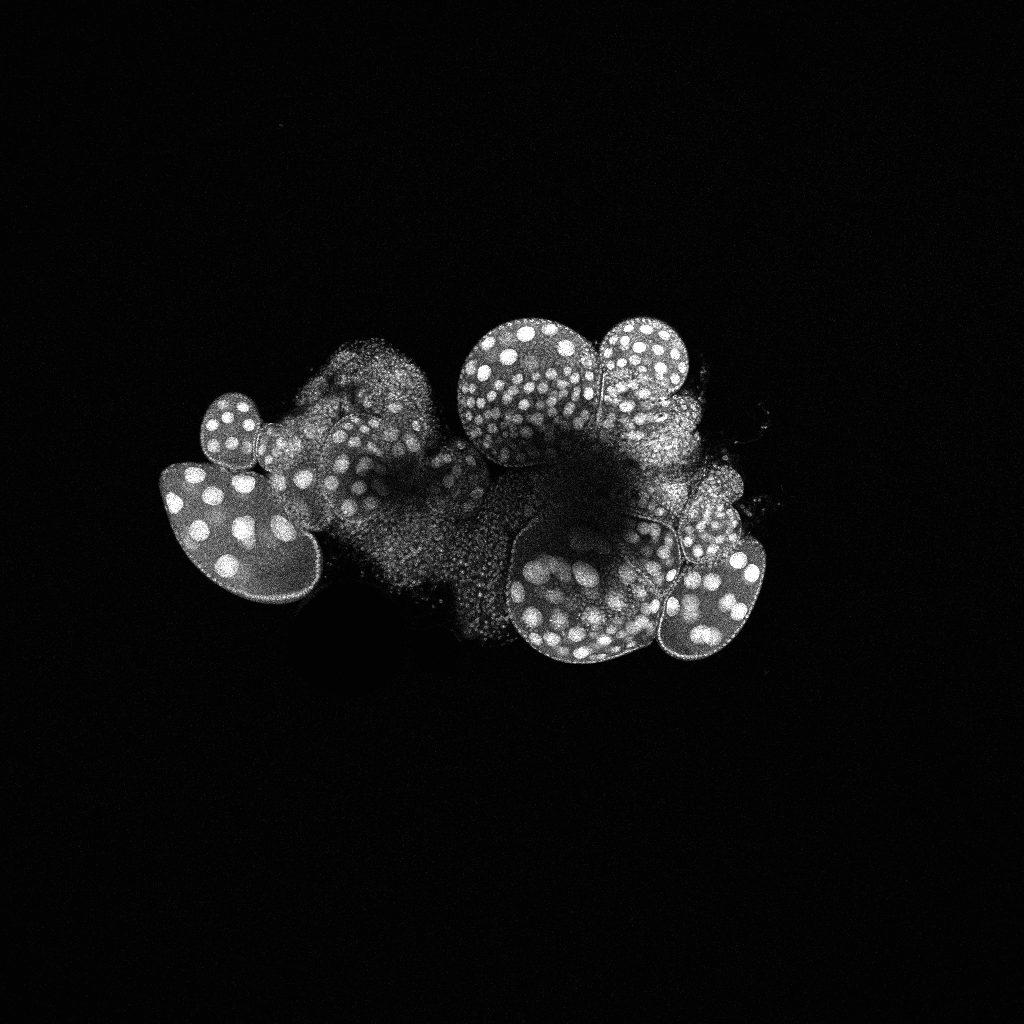

Supplement: Supplementary file 33 — Original Data [file 41419_2022_5195_MOESM33_ESM.tif]

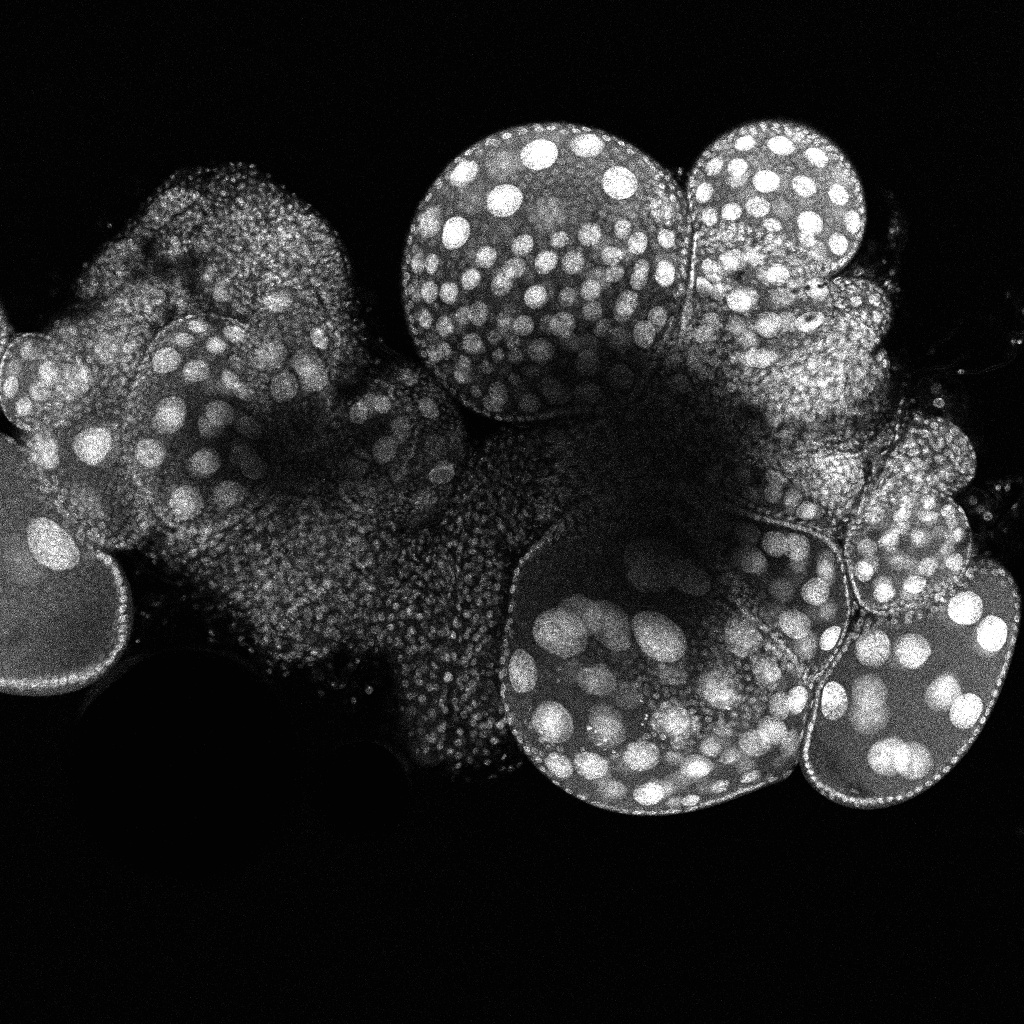

Supplement: Supplementary file 34 — Original Data [file 41419_2022_5195_MOESM34_ESM.tif]

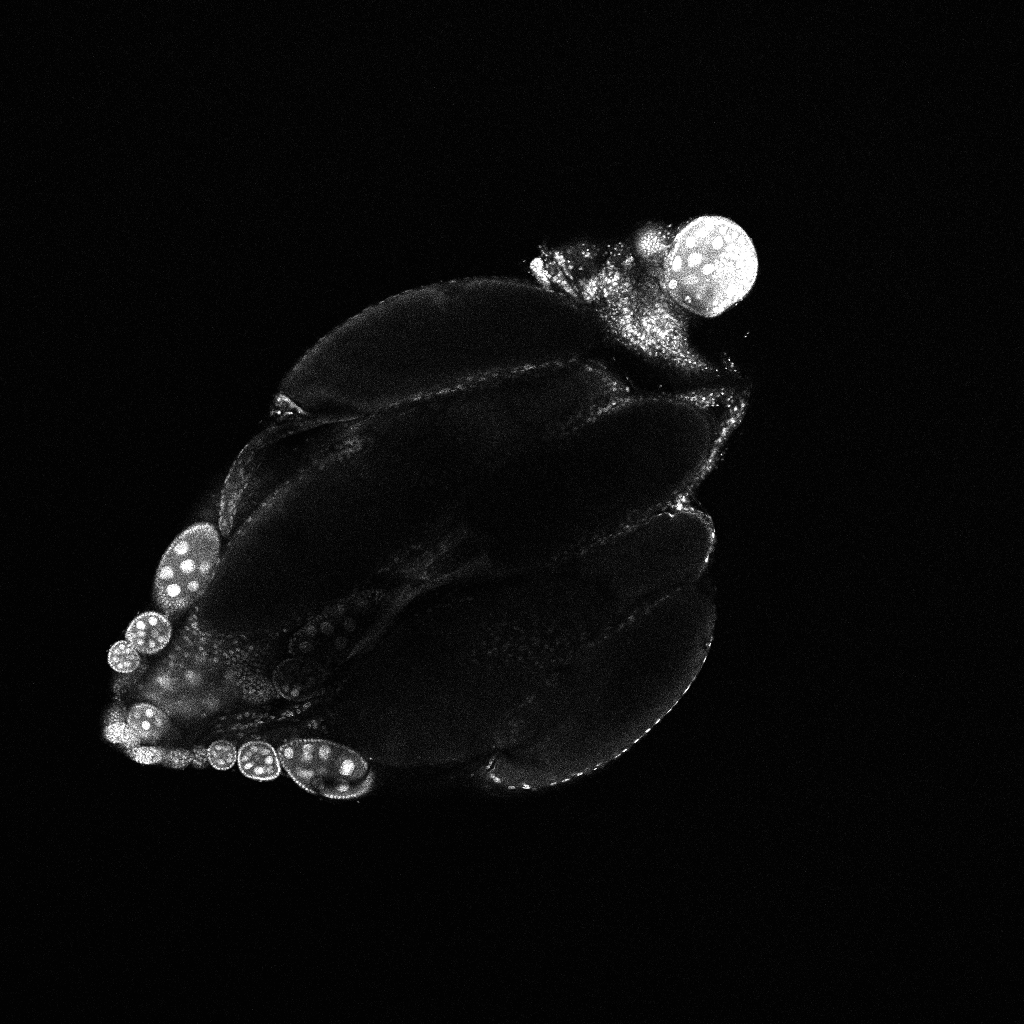

Supplement: Supplementary file 35 — Original Data [file 41419_2022_5195_MOESM35_ESM.tif]

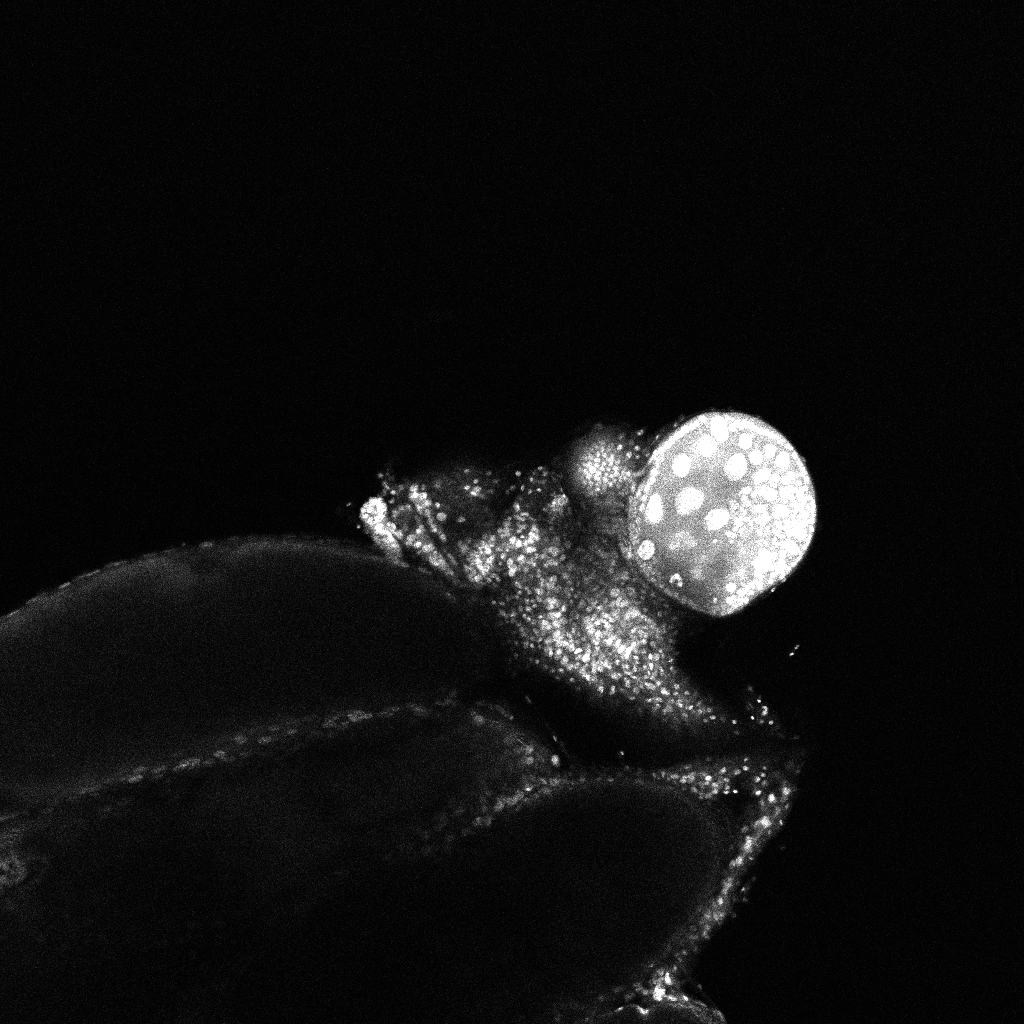

Supplement: Supplementary file 36 — Original Data [file 41419_2022_5195_MOESM36_ESM.tif]

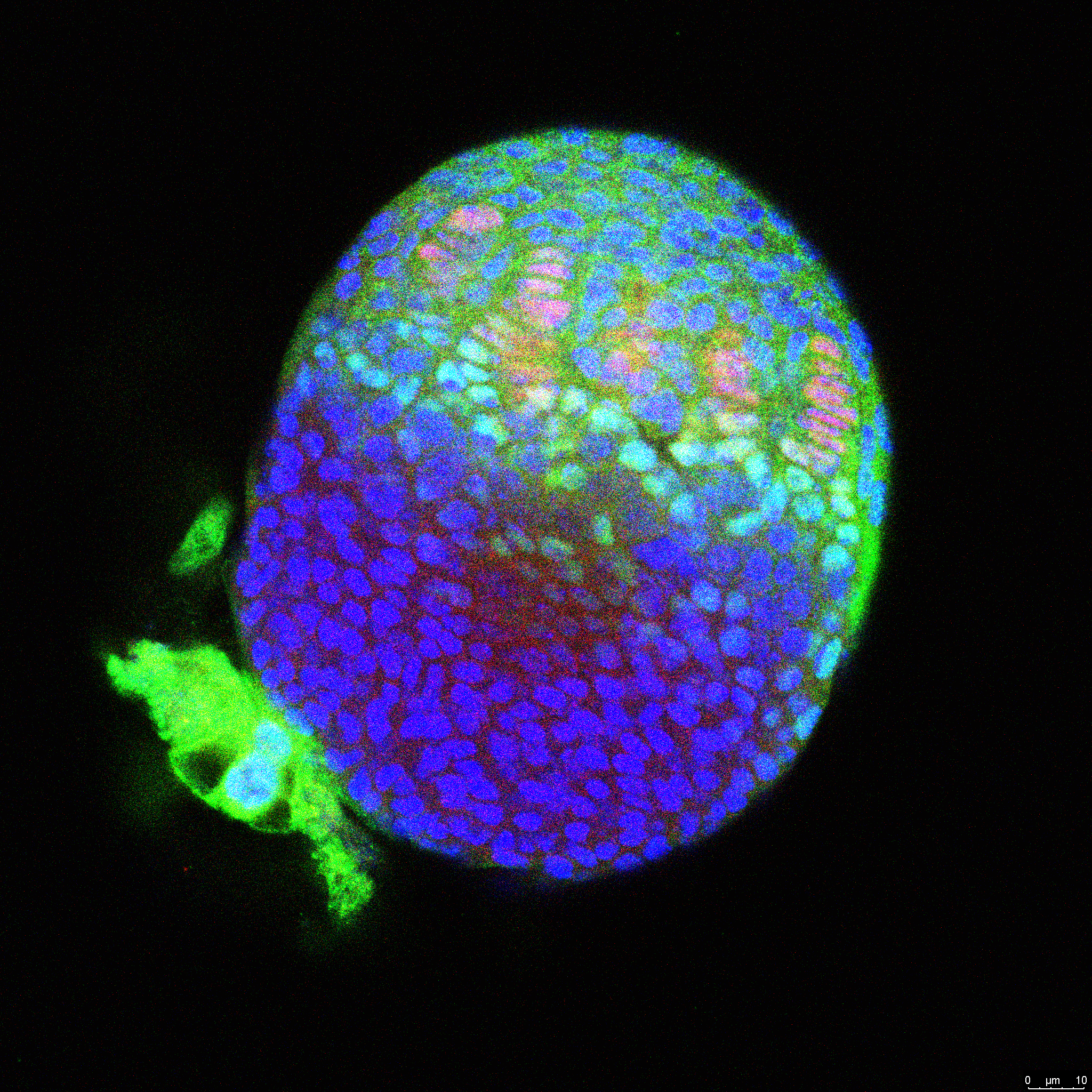

Supplement: Supplementary file 37 — Original Data [file 41419_2022_5195_MOESM37_ESM.tif]

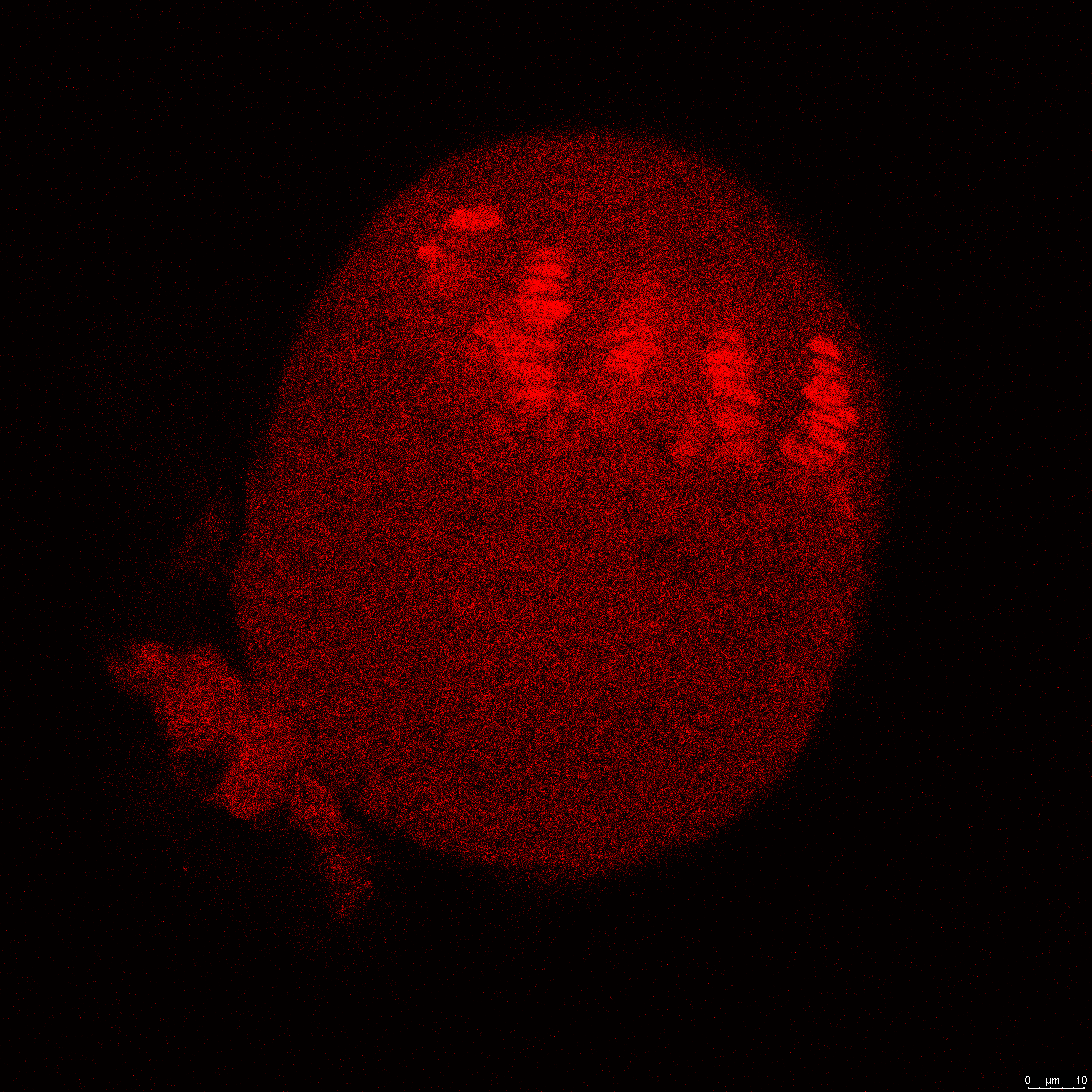

Supplement: Supplementary file 38 — Original Data [file 41419_2022_5195_MOESM38_ESM.tif]

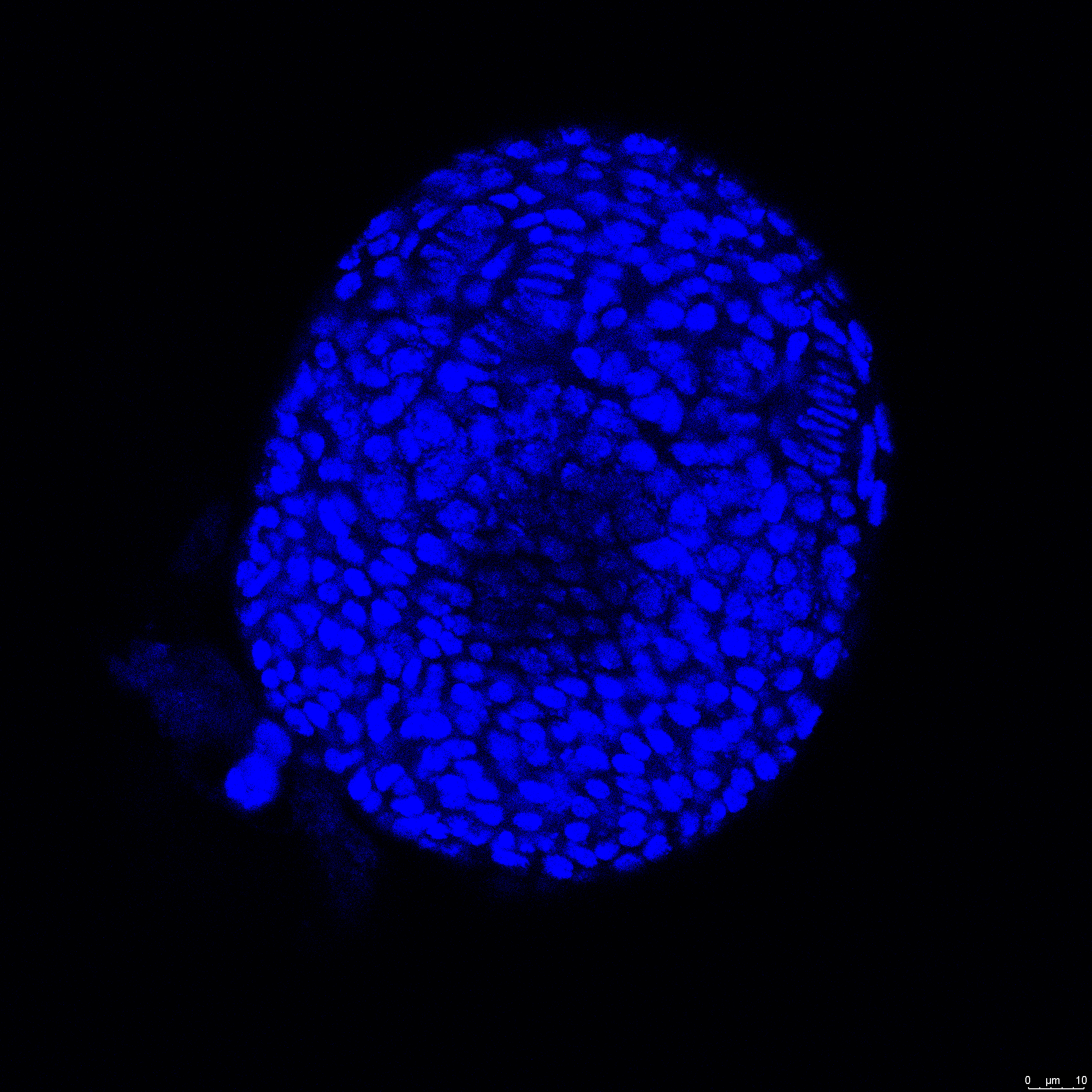

Supplement: Supplementary file 39 — Original Data [file 41419_2022_5195_MOESM39_ESM.tif]

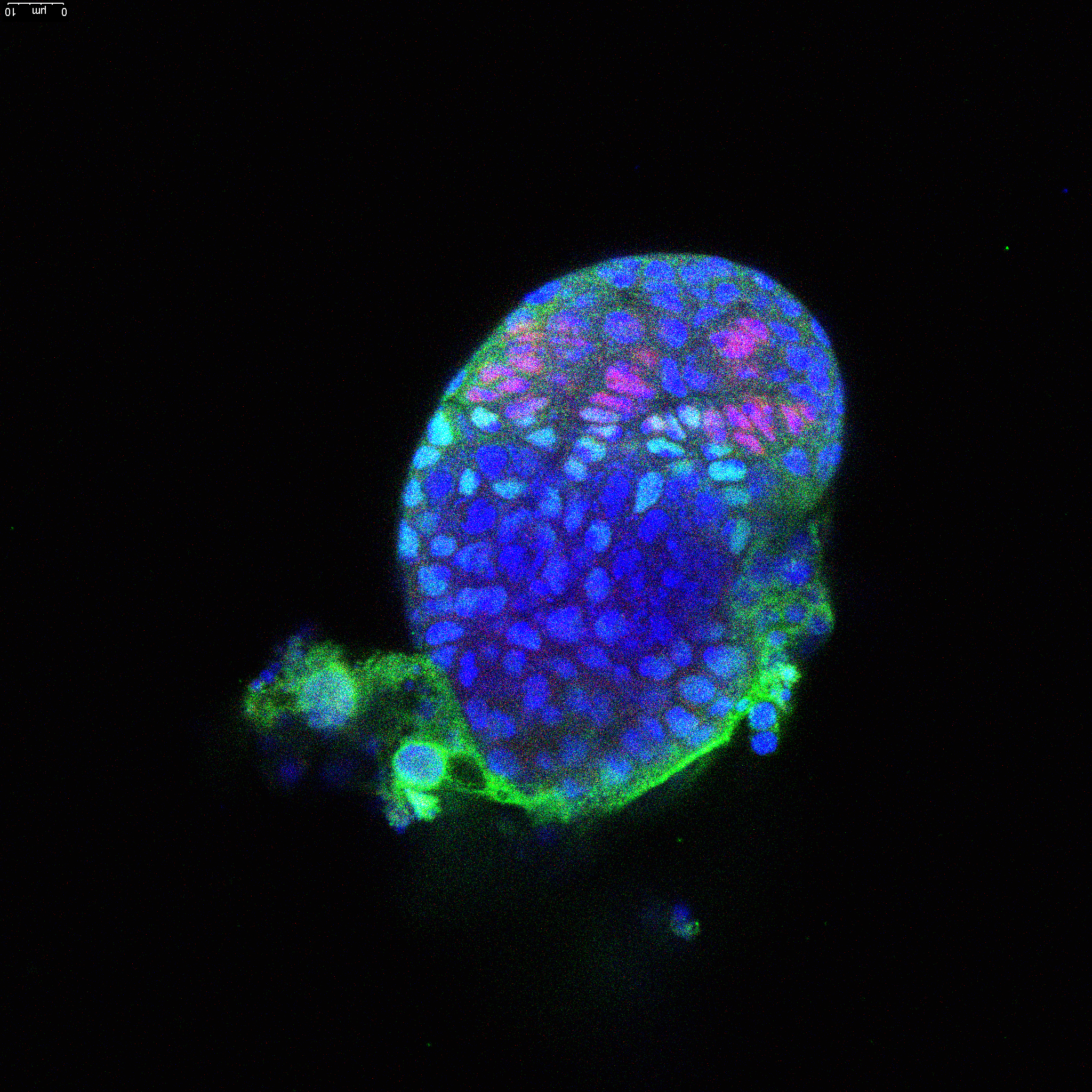

Supplement: Supplementary file 40 — Original Data [file 41419_2022_5195_MOESM40_ESM.tif]

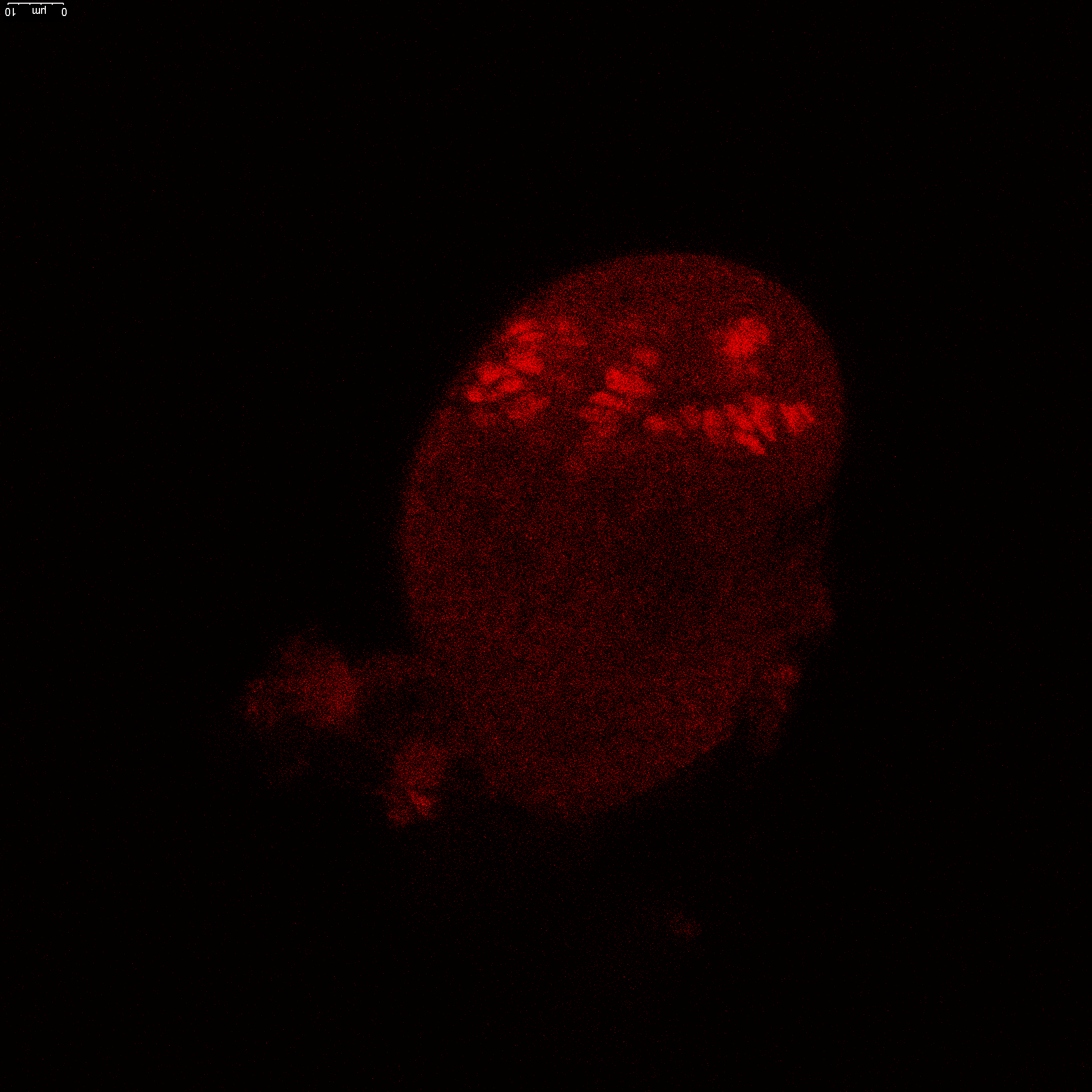

Supplement: Supplementary file 41 — Original Data [file 41419_2022_5195_MOESM41_ESM.tif]

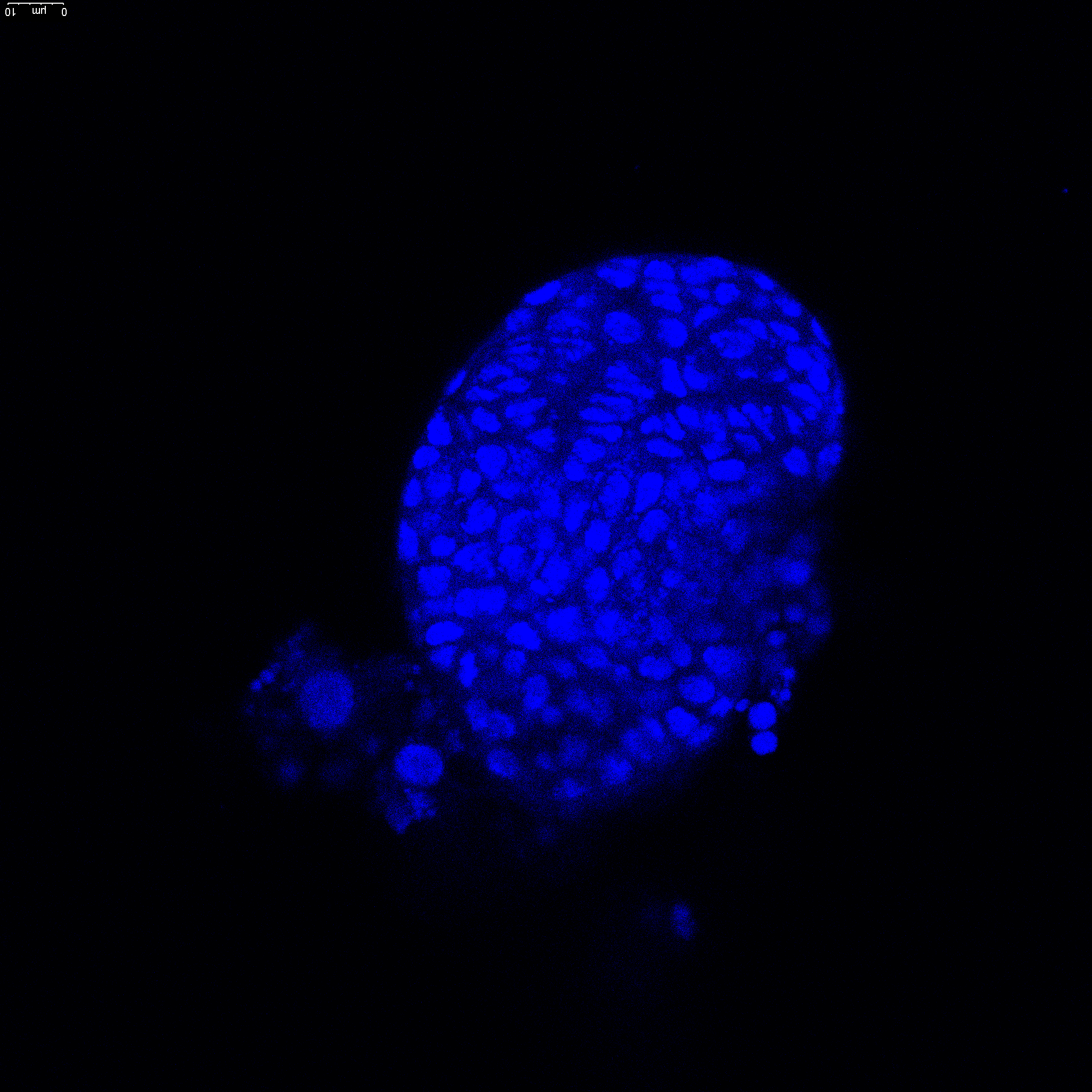

Supplement: Supplementary file 42 — Original Data [file 41419_2022_5195_MOESM42_ESM.tif]

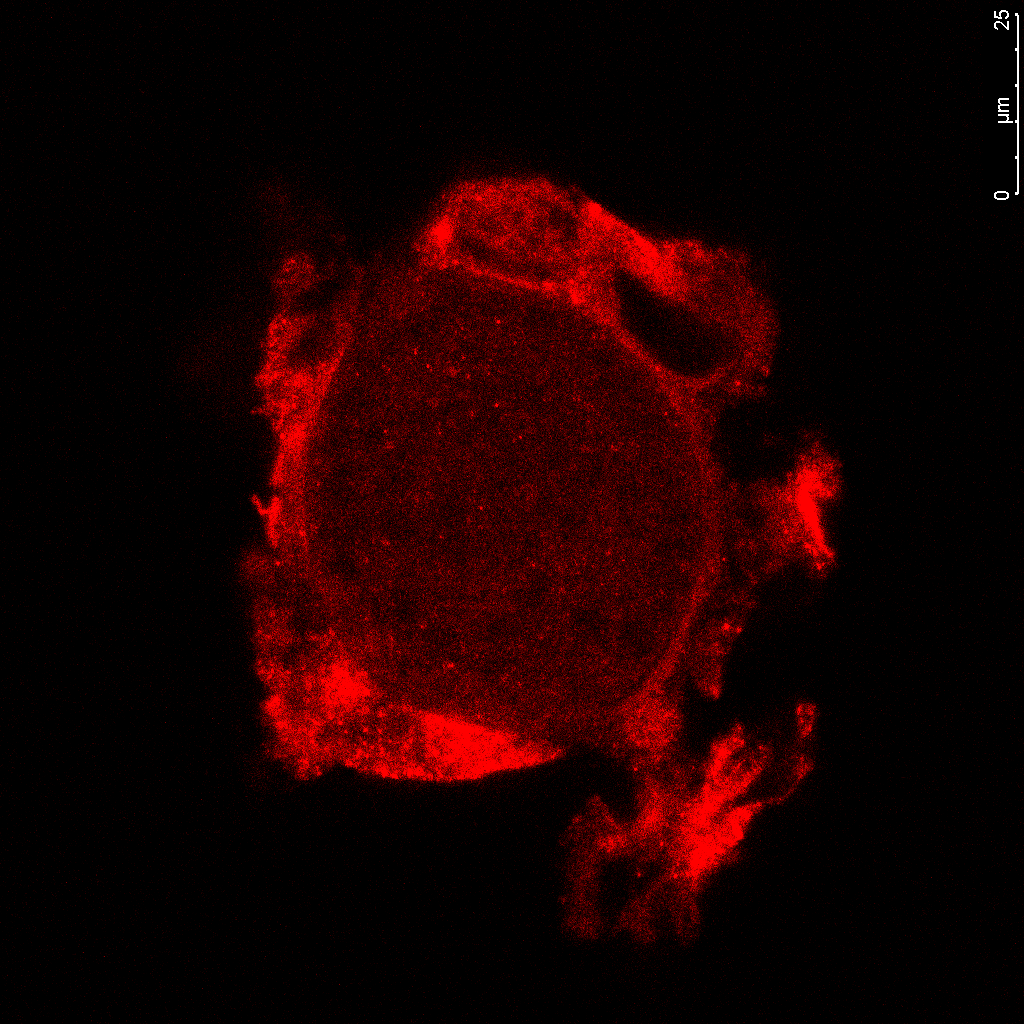

Supplement: Supplementary file 43 — Original Data [file 41419_2022_5195_MOESM43_ESM.tif]

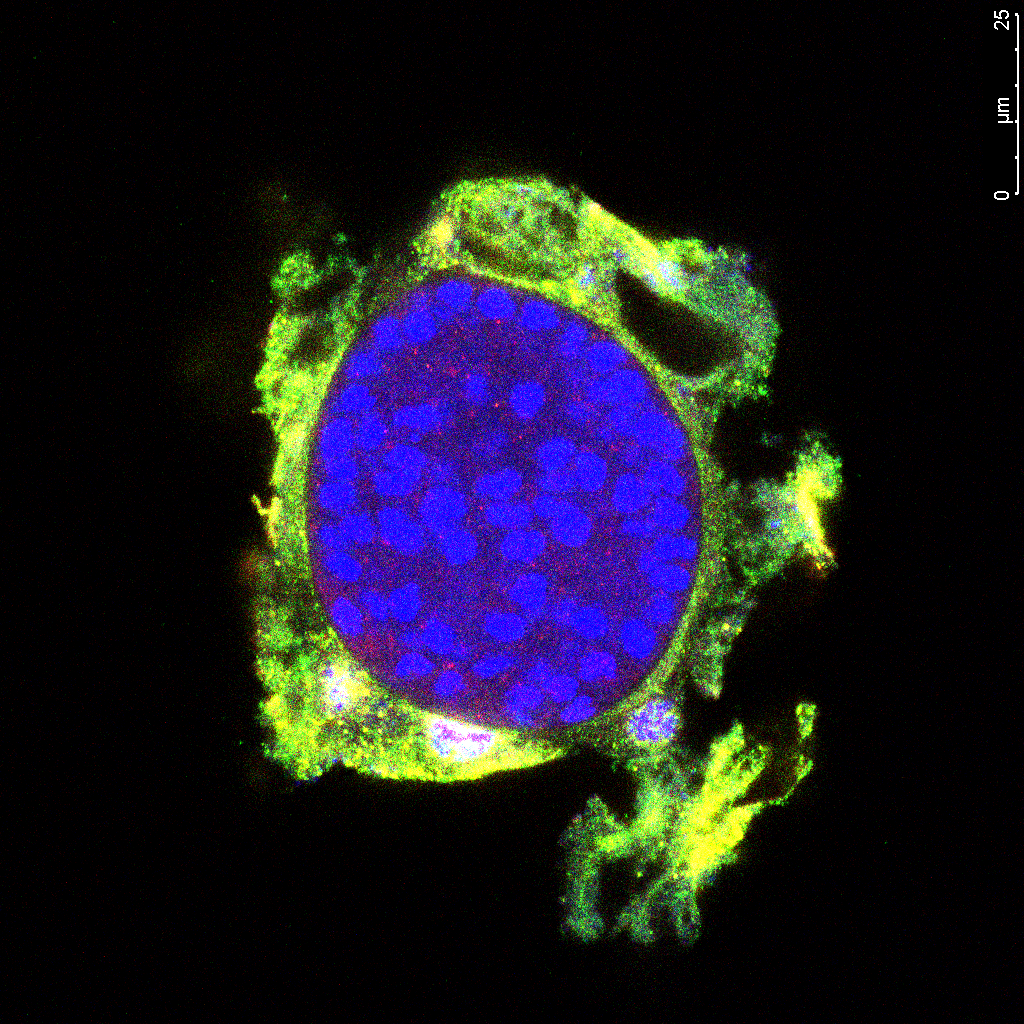

Supplement: Supplementary file 44 — Original Data [file 41419_2022_5195_MOESM44_ESM.tif]

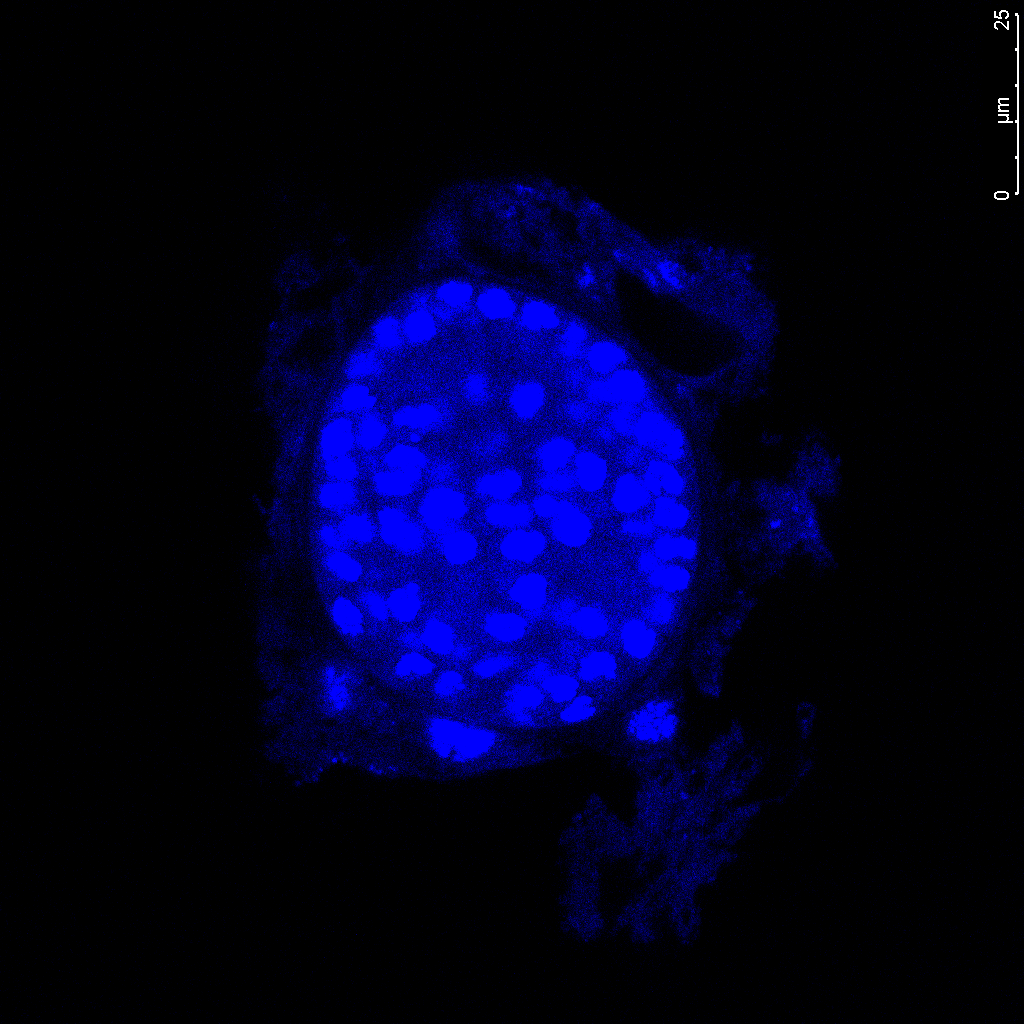

Supplement: Supplementary file 45 — Original Data [file 41419_2022_5195_MOESM45_ESM.tif]

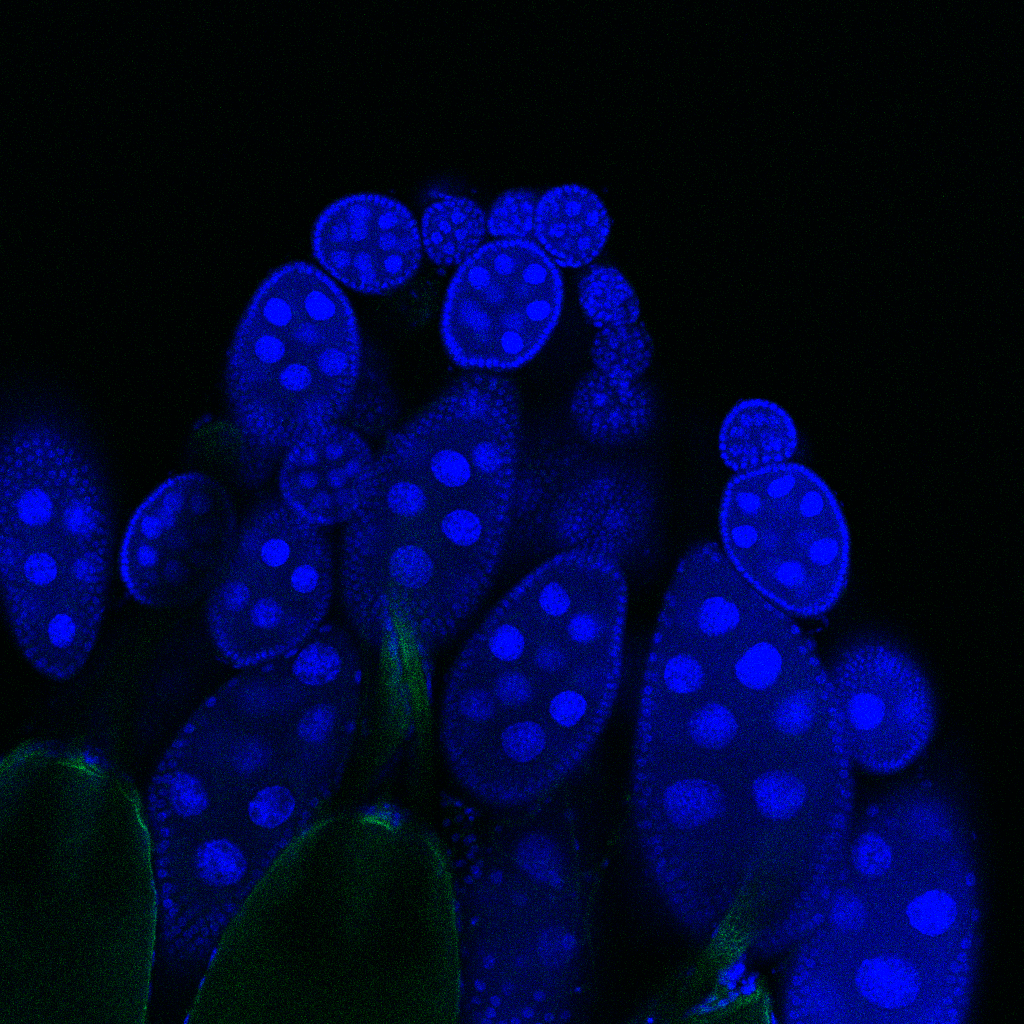

Supplement: Supplementary file 46 — Original Data [file 41419_2022_5195_MOESM46_ESM.tif]

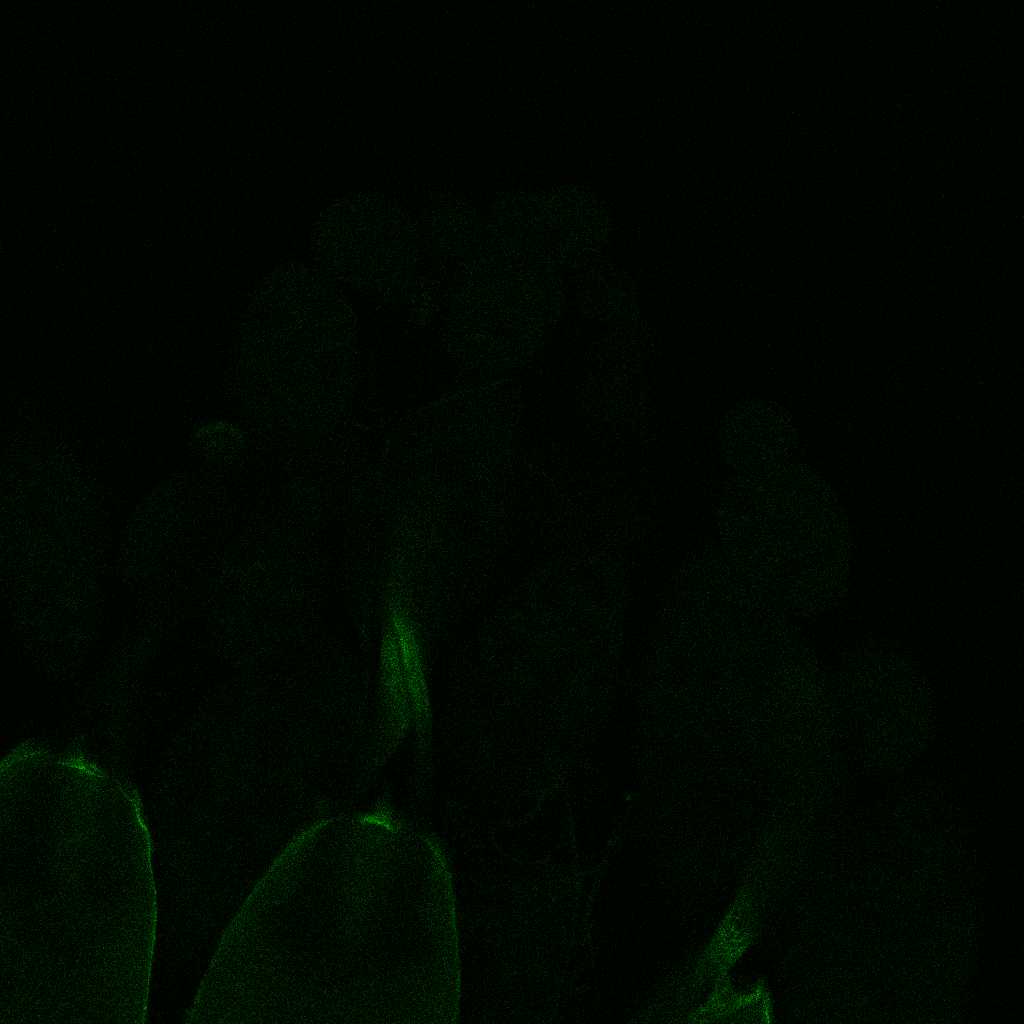

Supplement: Supplementary file 47 — Original Data [file 41419_2022_5195_MOESM47_ESM.tif]

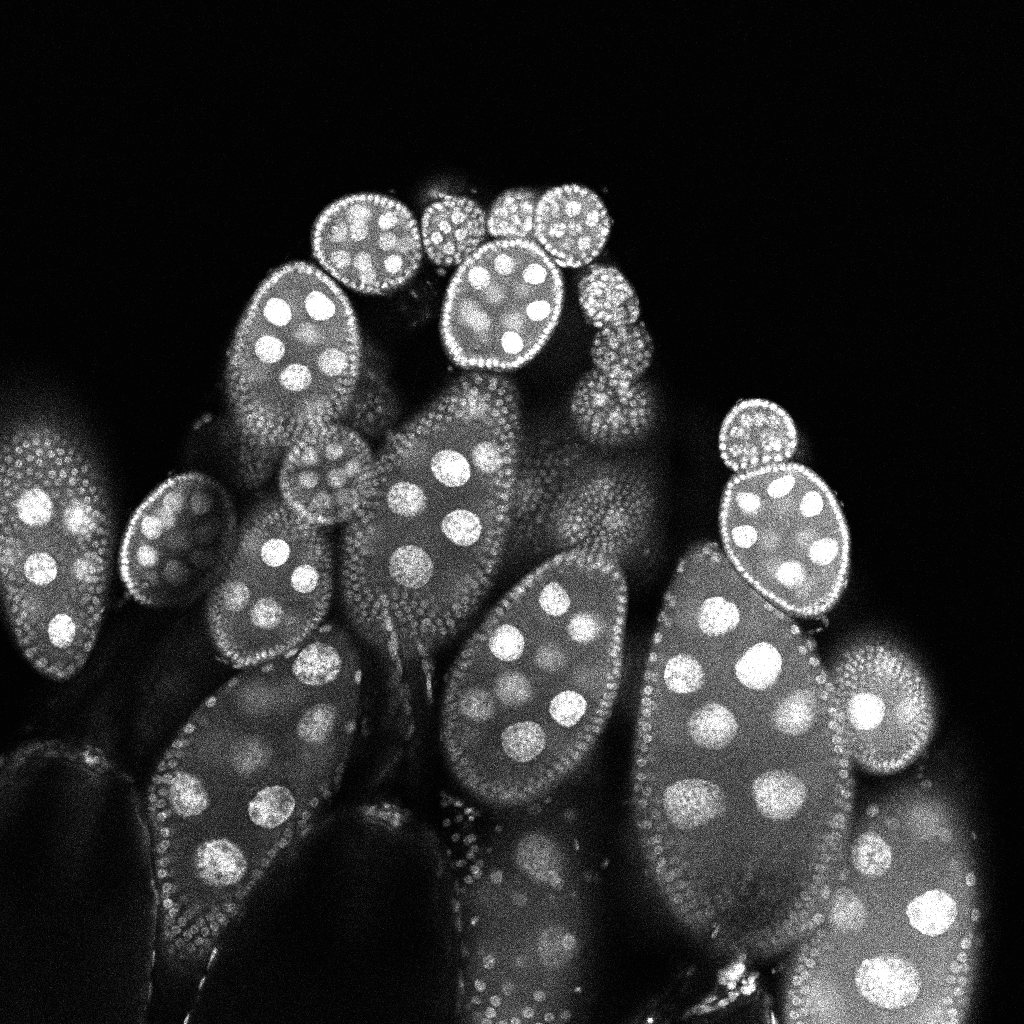

Supplement: Supplementary file 48 — Original Data [file 41419_2022_5195_MOESM48_ESM.tif]

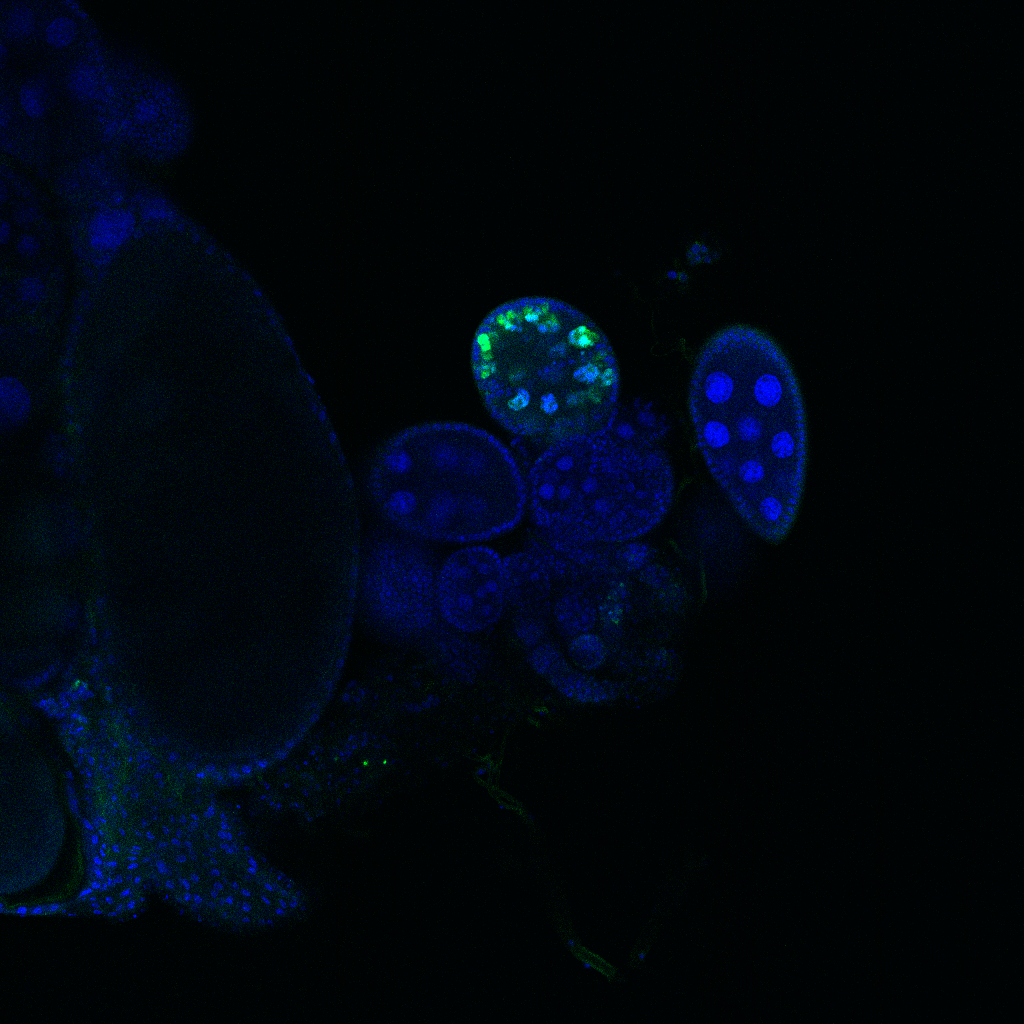

Supplement: Supplementary file 49 — Original Data [file 41419_2022_5195_MOESM49_ESM.tif]

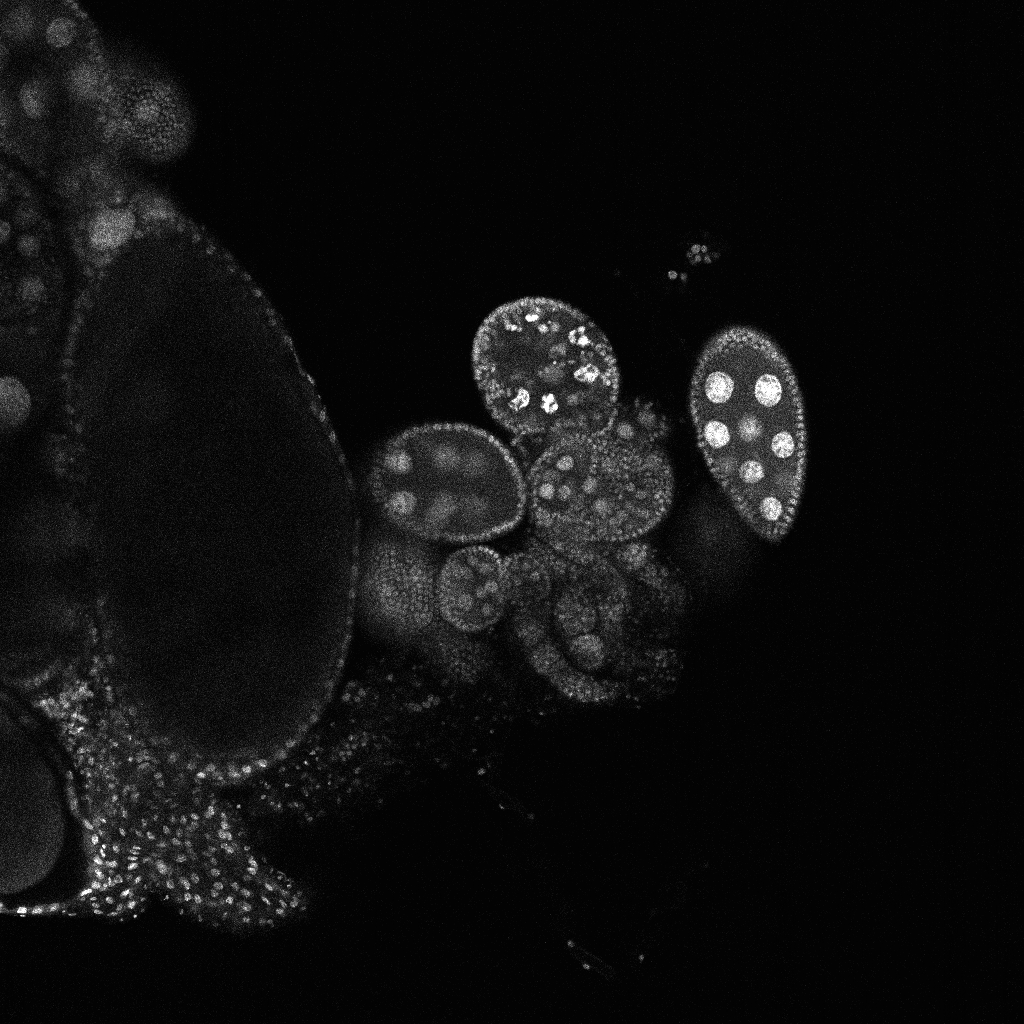

Supplement: Supplementary file 50 — Original Data [file 41419_2022_5195_MOESM50_ESM.tif]

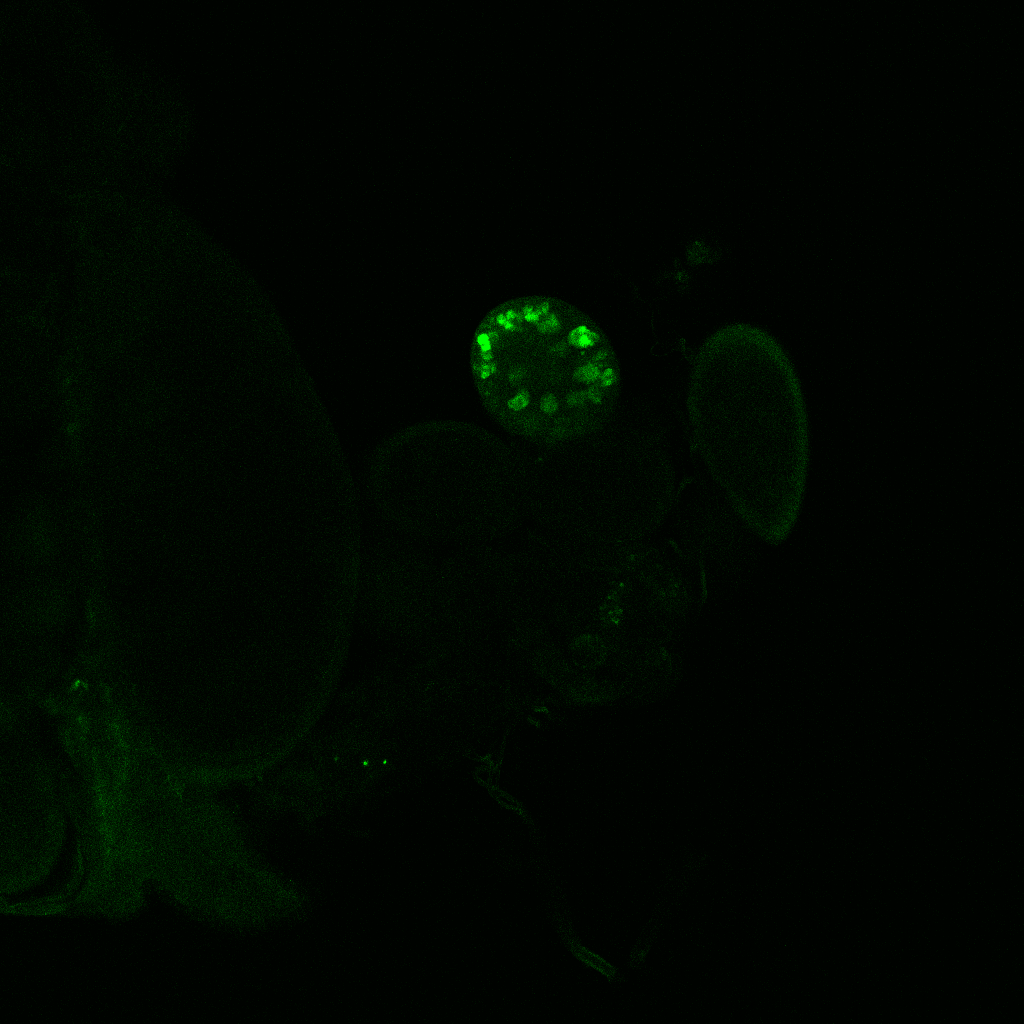

Supplement: Supplementary file 51 — Original Data [file 41419_2022_5195_MOESM51_ESM.tif]

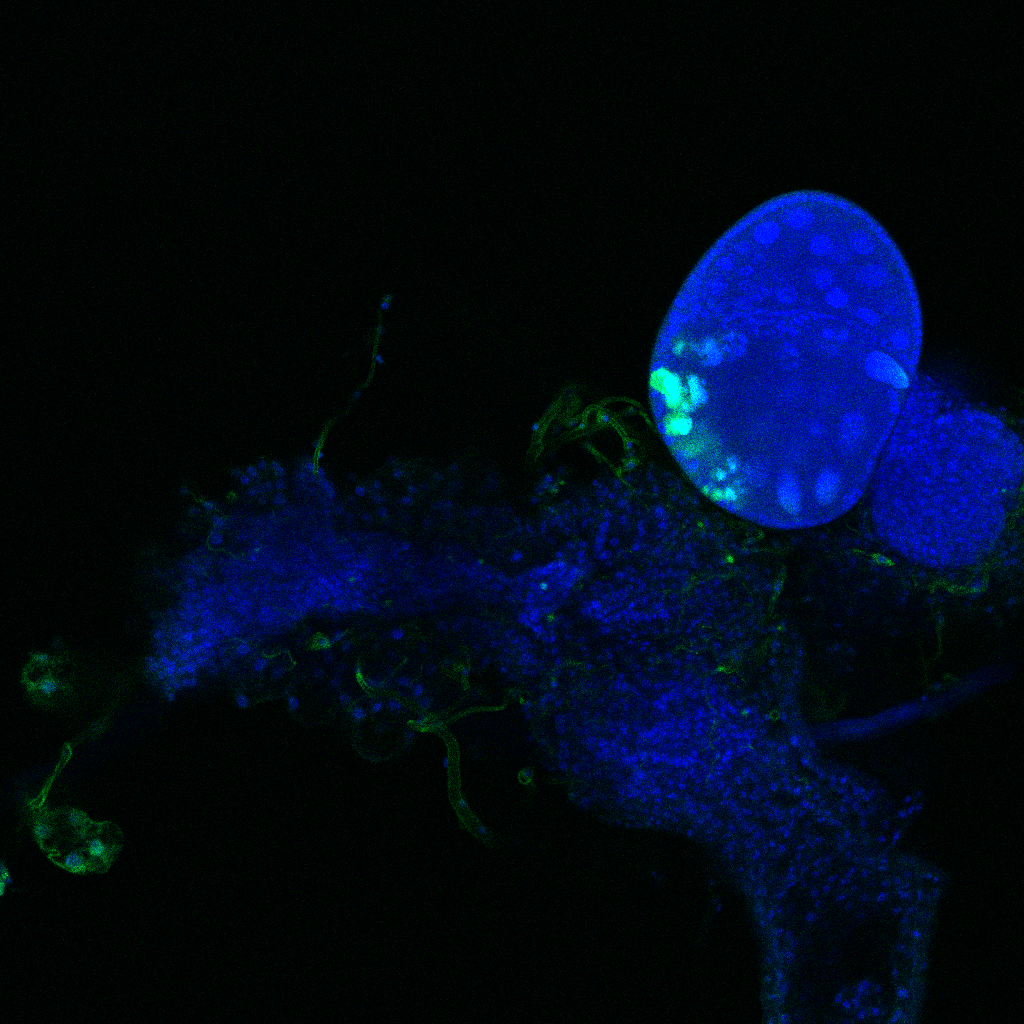

Supplement: Supplementary file 52 — Original Data [file 41419_2022_5195_MOESM52_ESM.tif]

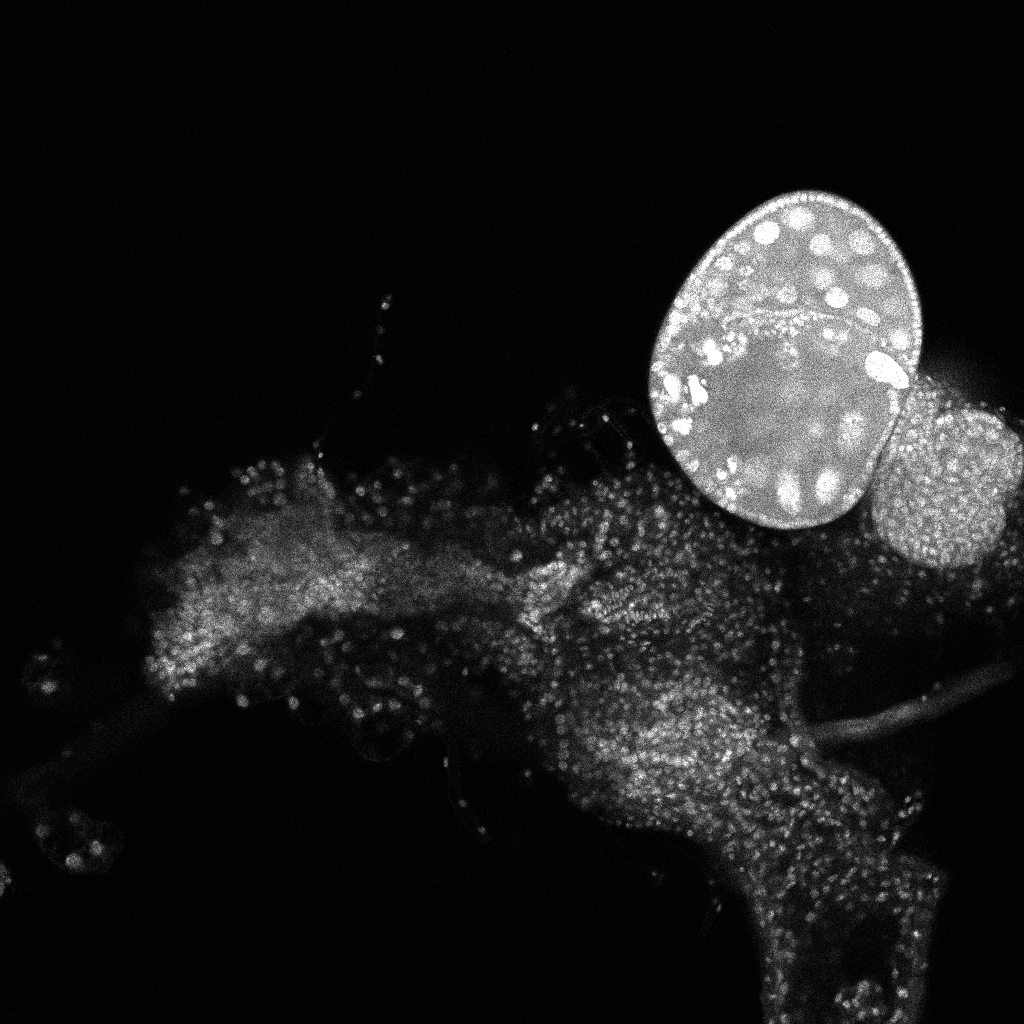

Supplement: Supplementary file 53 — Original Data [file 41419_2022_5195_MOESM53_ESM.tif]

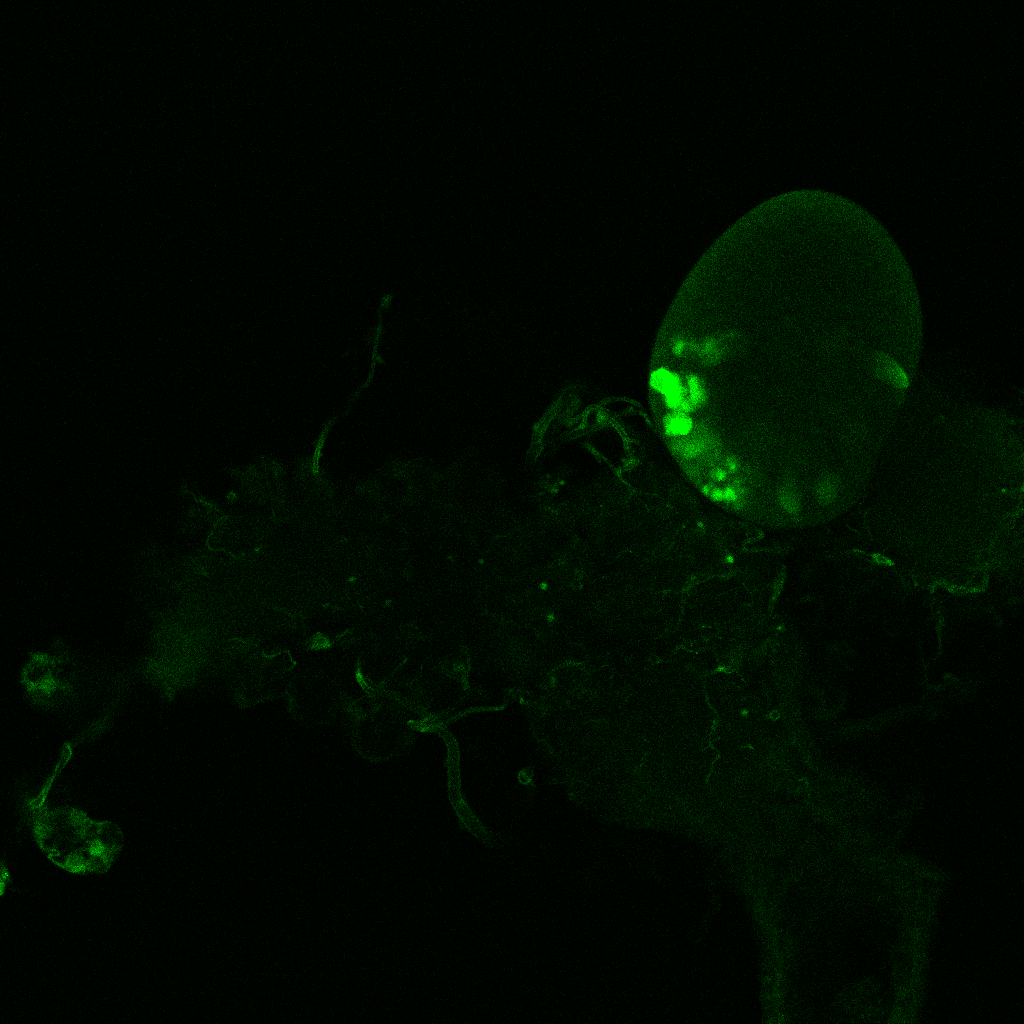

Supplement: Supplementary file 54 — Original Data [file 41419_2022_5195_MOESM54_ESM.tif]

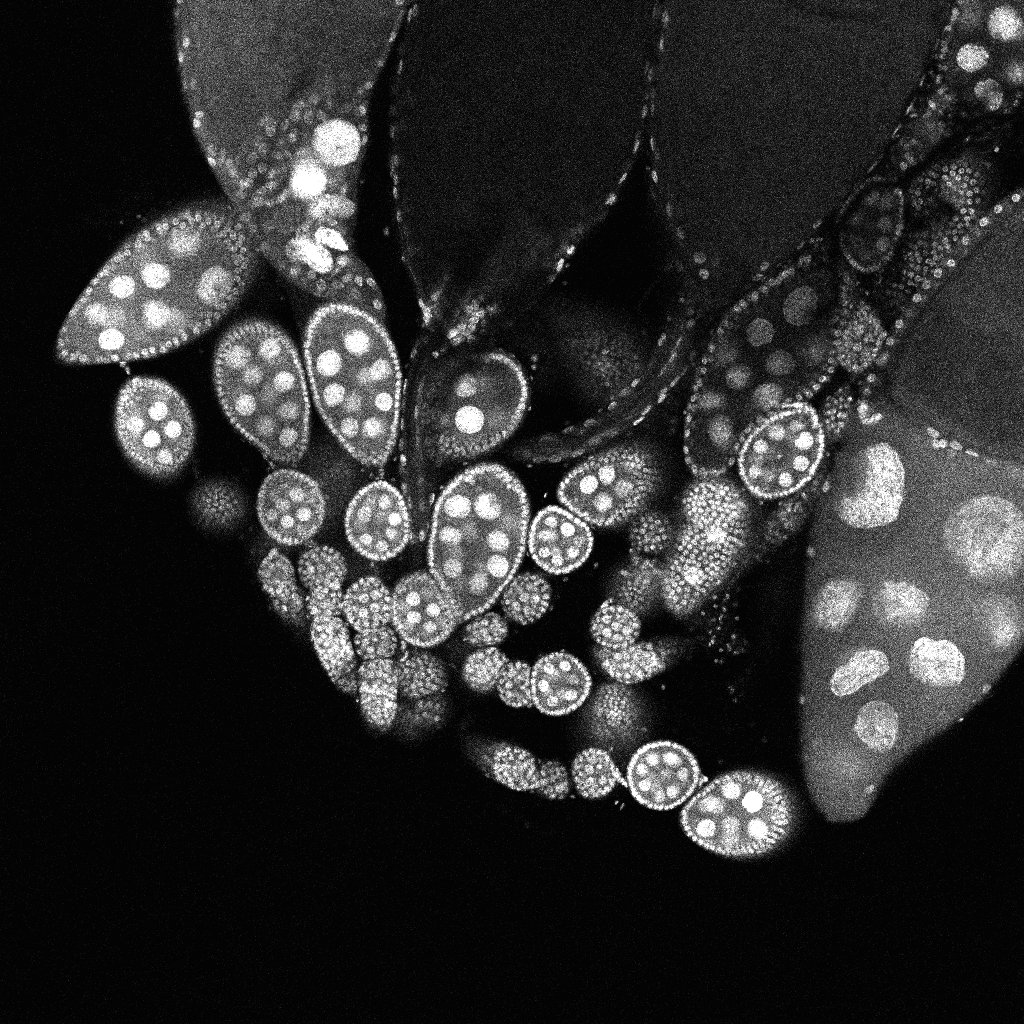

Supplement: Supplementary file 55 — Original Data [file 41419_2022_5195_MOESM55_ESM.tif]

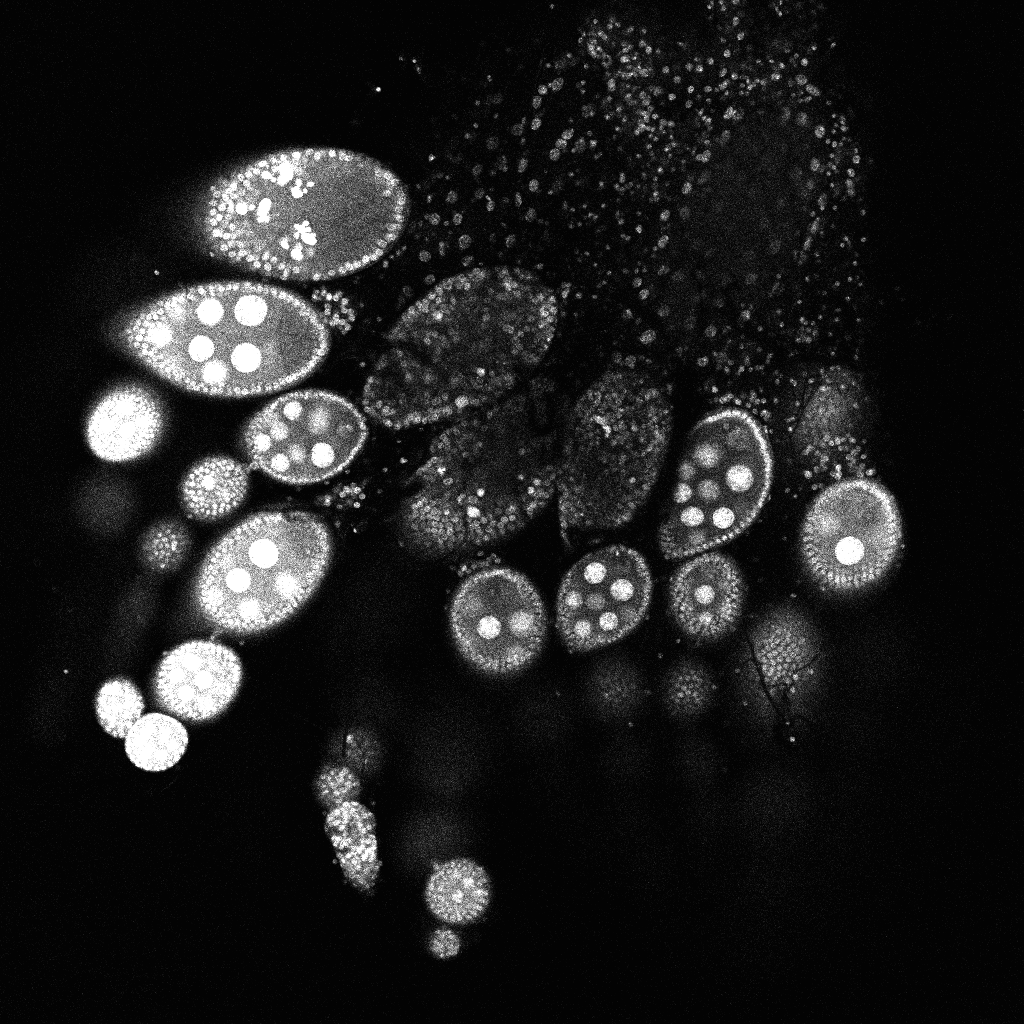

Supplement: Supplementary file 56 — Original Data [file 41419_2022_5195_MOESM56_ESM.tif]

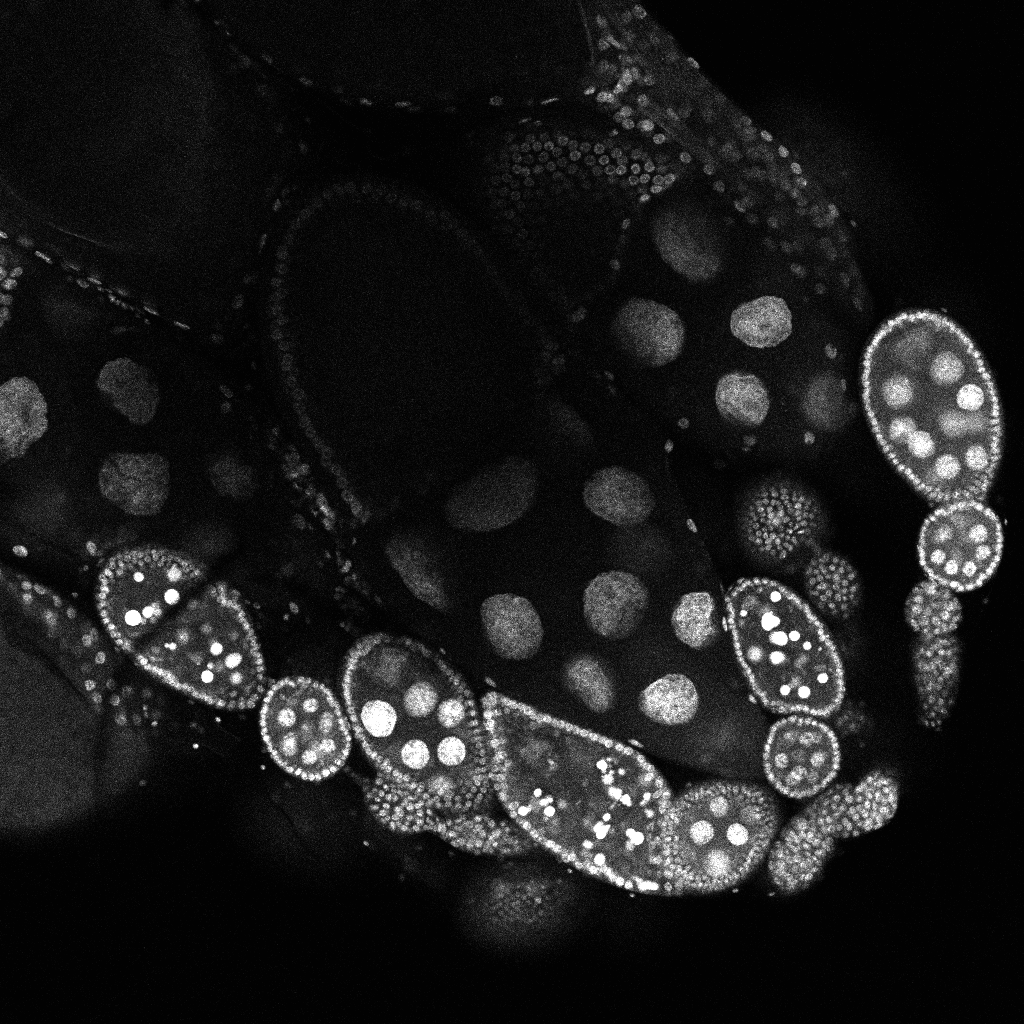

Supplement: Supplementary file 57 — Original Data [file 41419_2022_5195_MOESM57_ESM.tif]

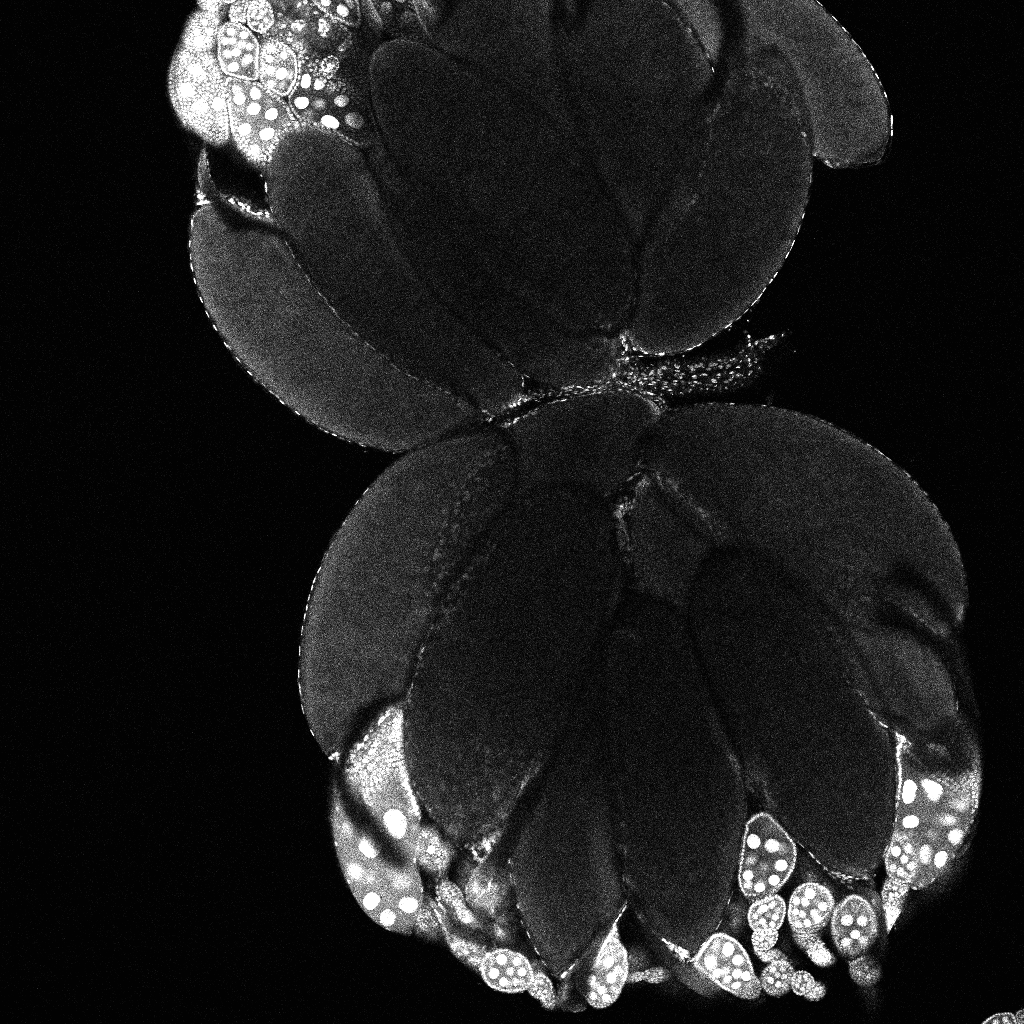

Supplement: Supplementary file 58 — Original Data [file 41419_2022_5195_MOESM58_ESM.tif]

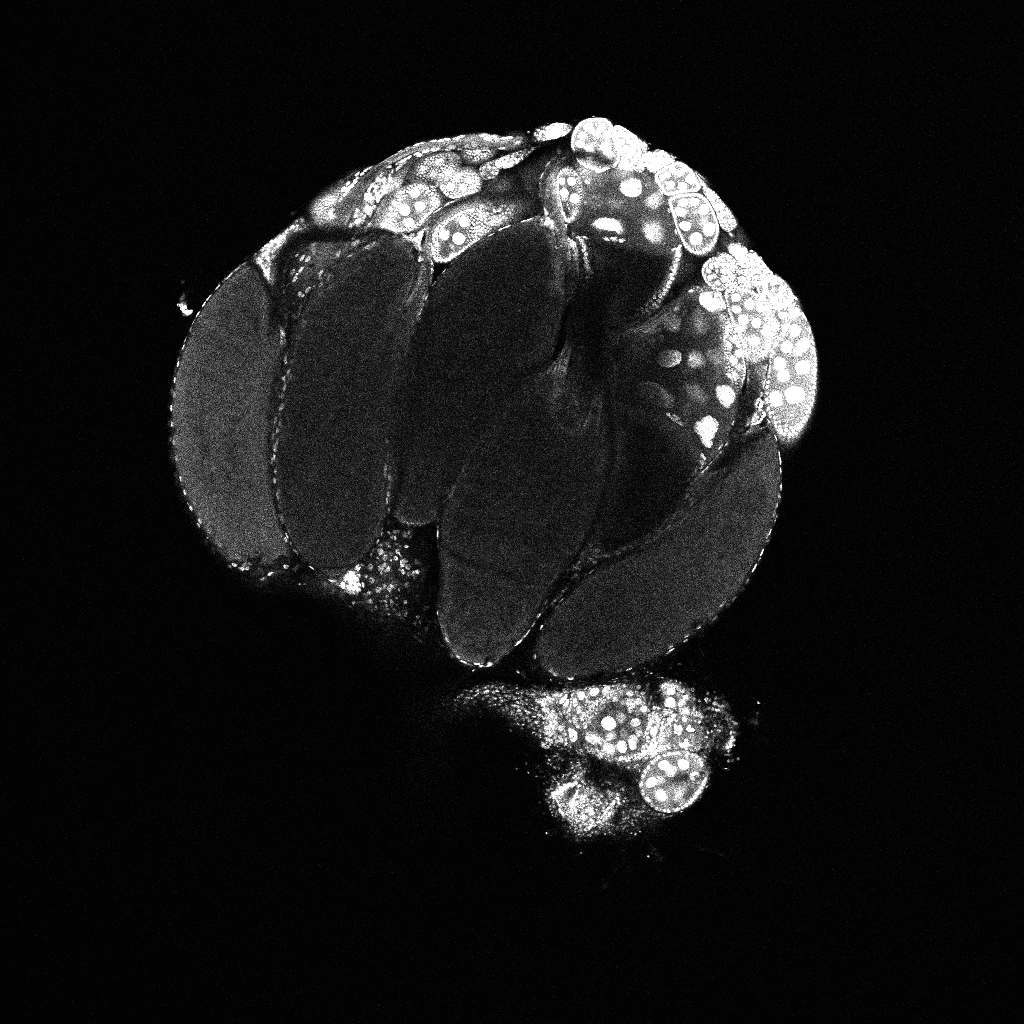

Supplement: Supplementary file 59 — Original Data [file 41419_2022_5195_MOESM59_ESM.tif]

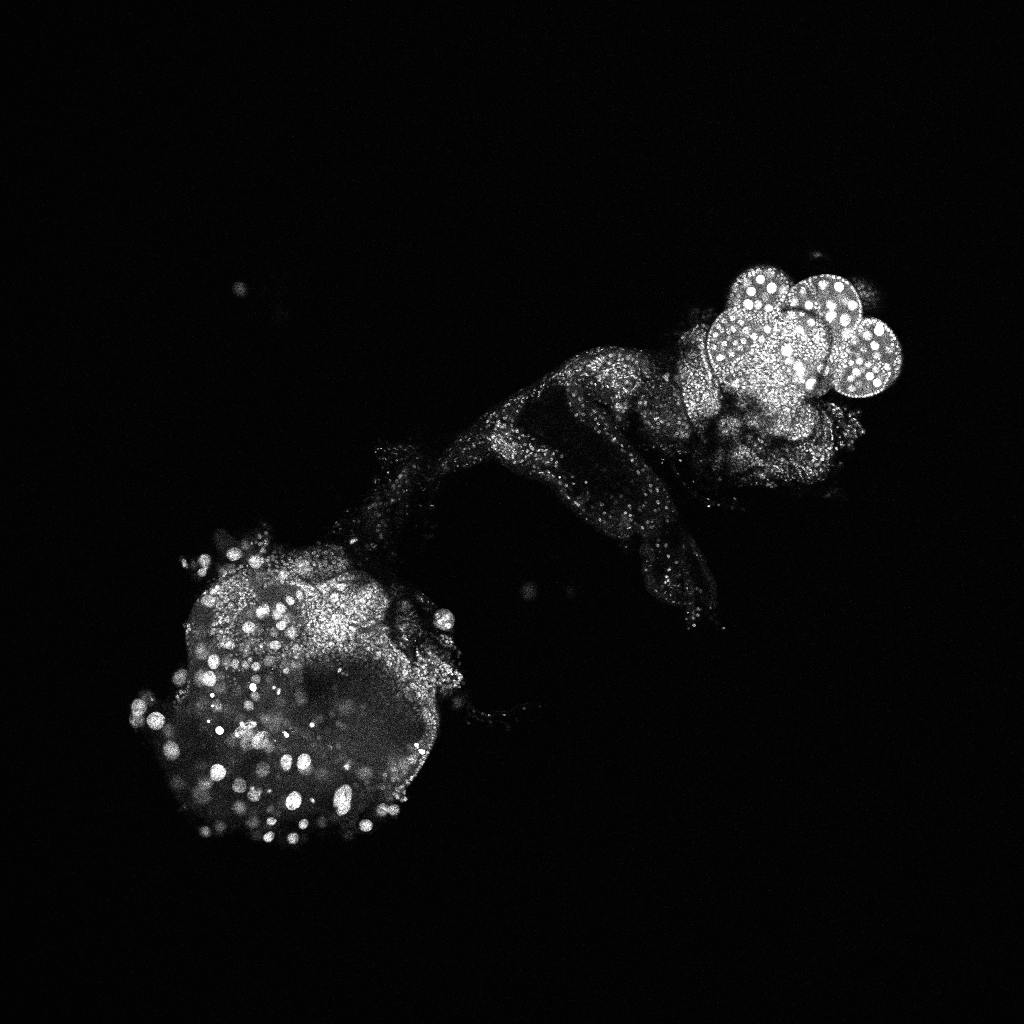

Supplement: Supplementary file 60 — Original Data [file 41419_2022_5195_MOESM60_ESM.tif]

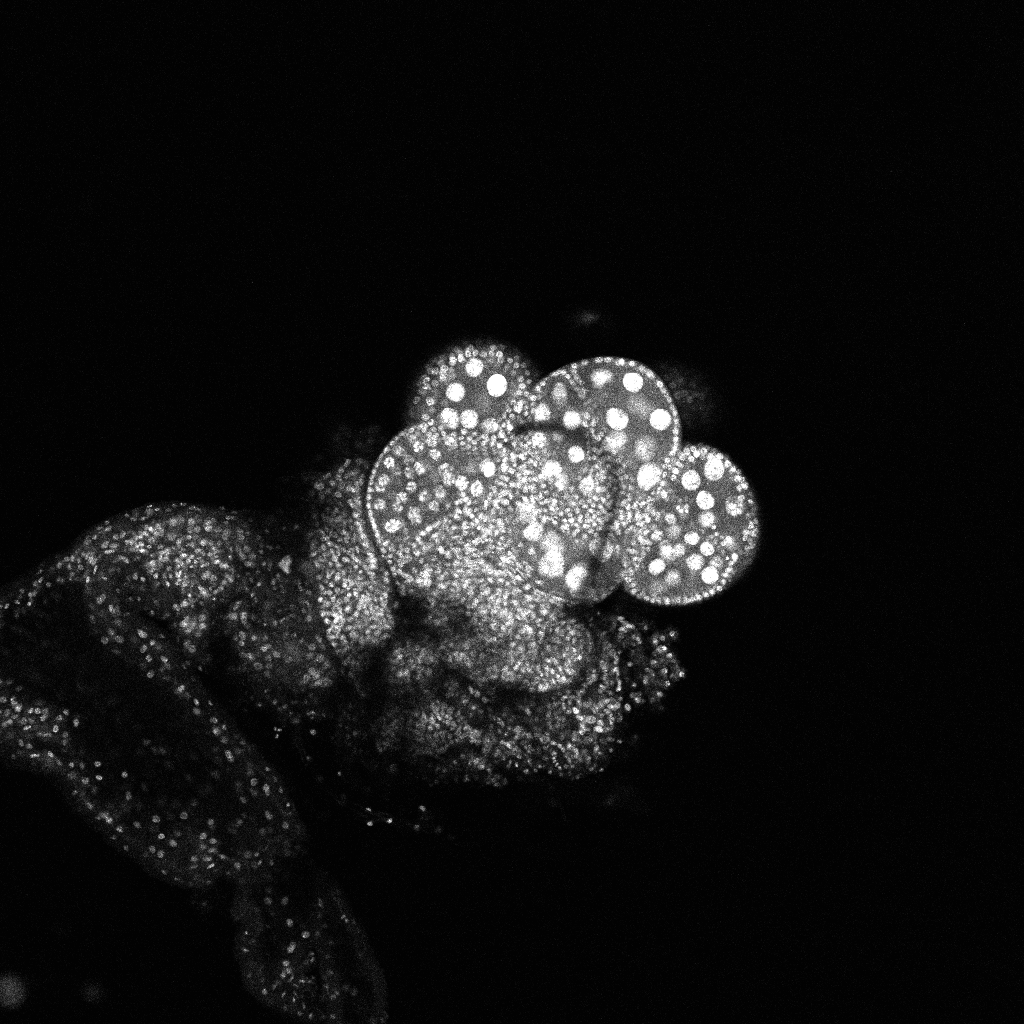

Supplement: Supplementary file 61 — Original Data [file 41419_2022_5195_MOESM61_ESM.tif]

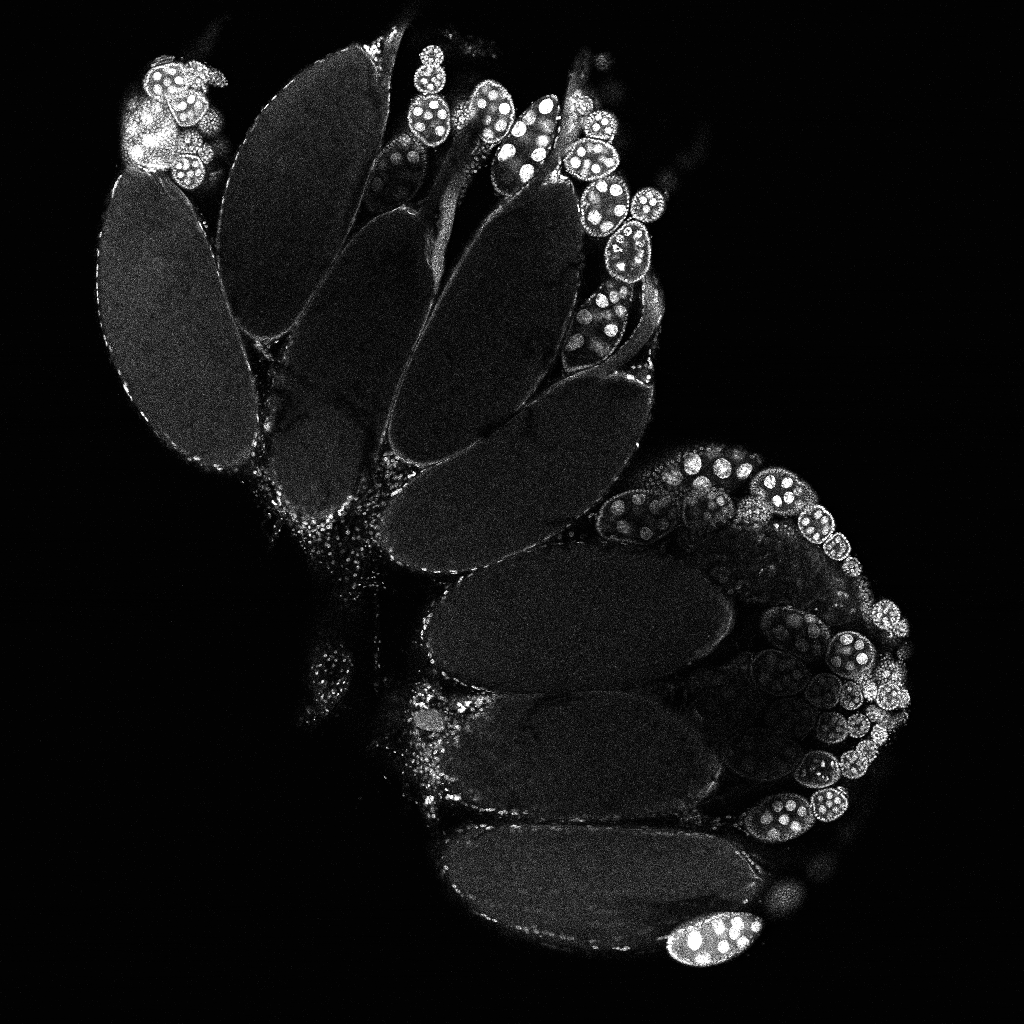

Supplement: Supplementary file 62 — Original Data [file 41419_2022_5195_MOESM62_ESM.tif]

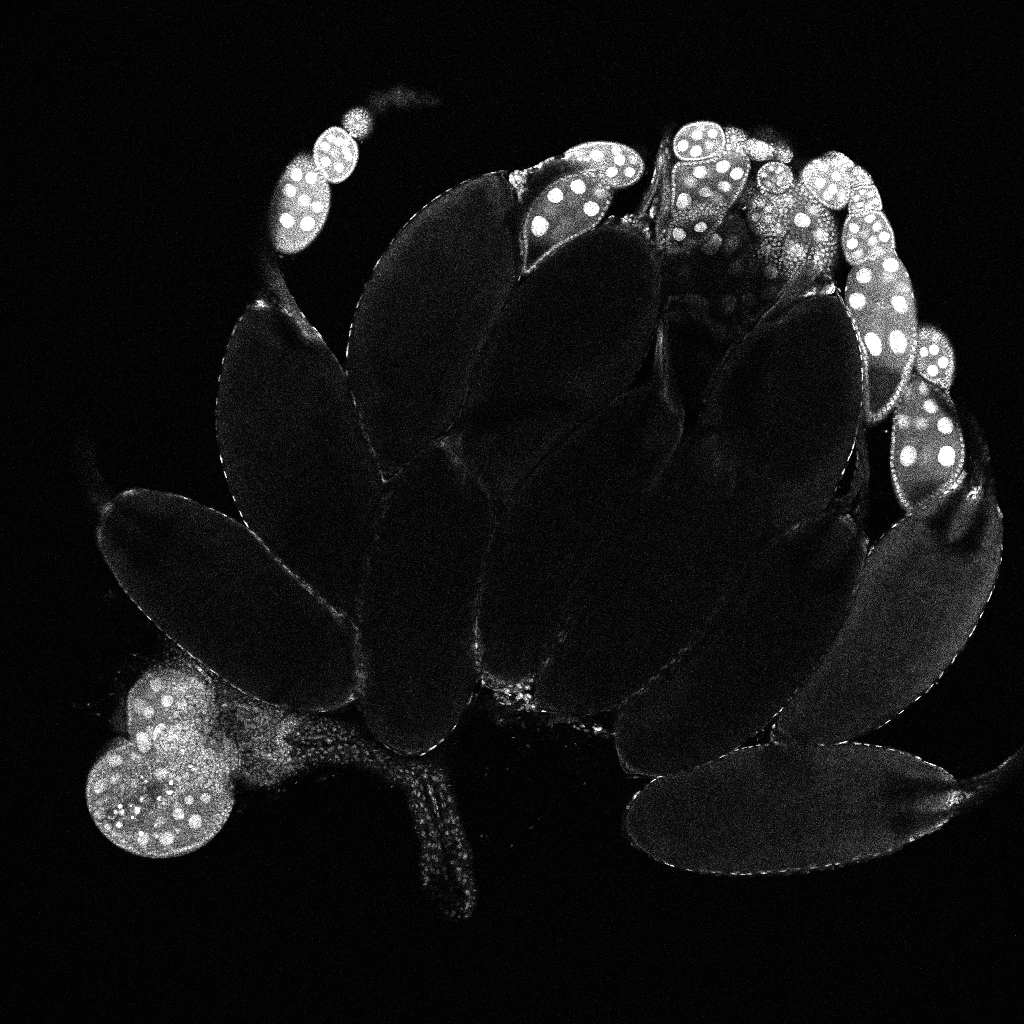

Supplement: Supplementary file 63 — Original Data [file 41419_2022_5195_MOESM63_ESM.tif]

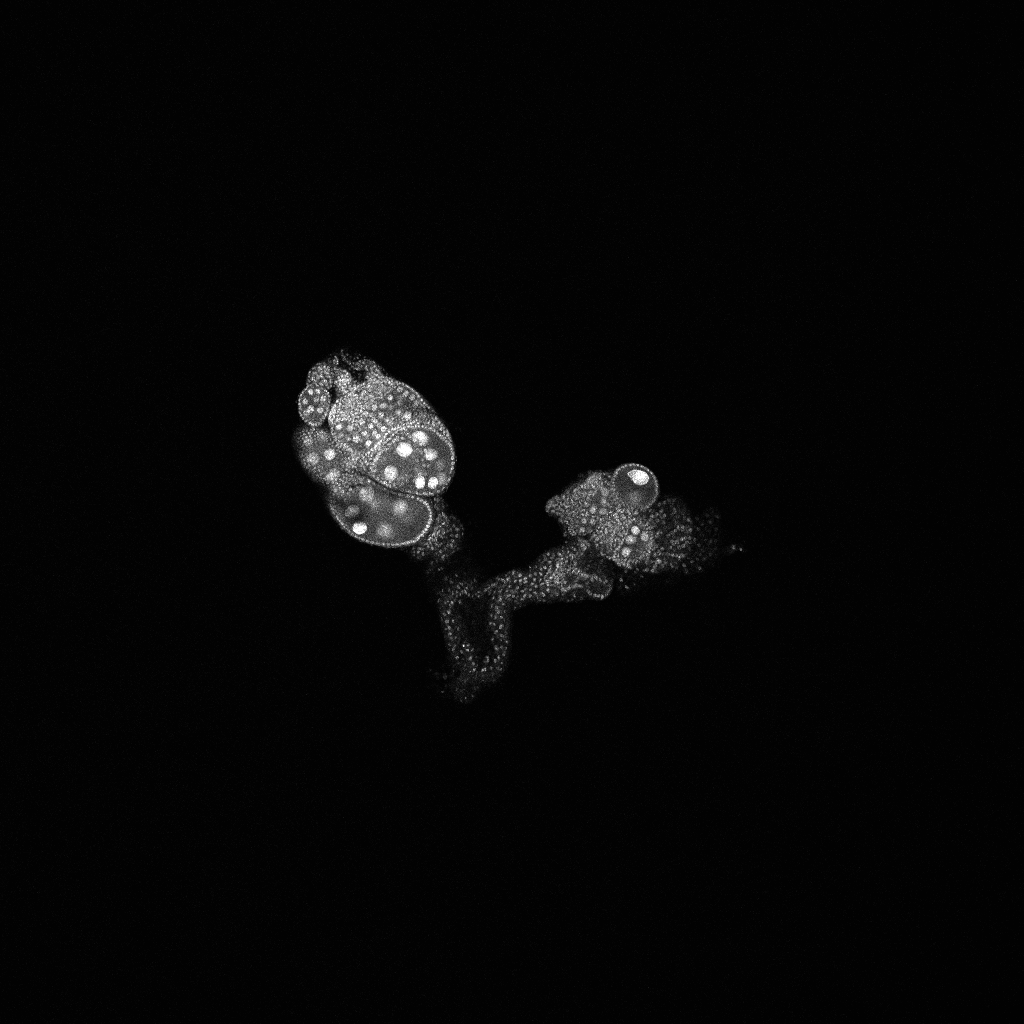

Supplement: Supplementary file 64 — Original Data [file 41419_2022_5195_MOESM64_ESM.tif]

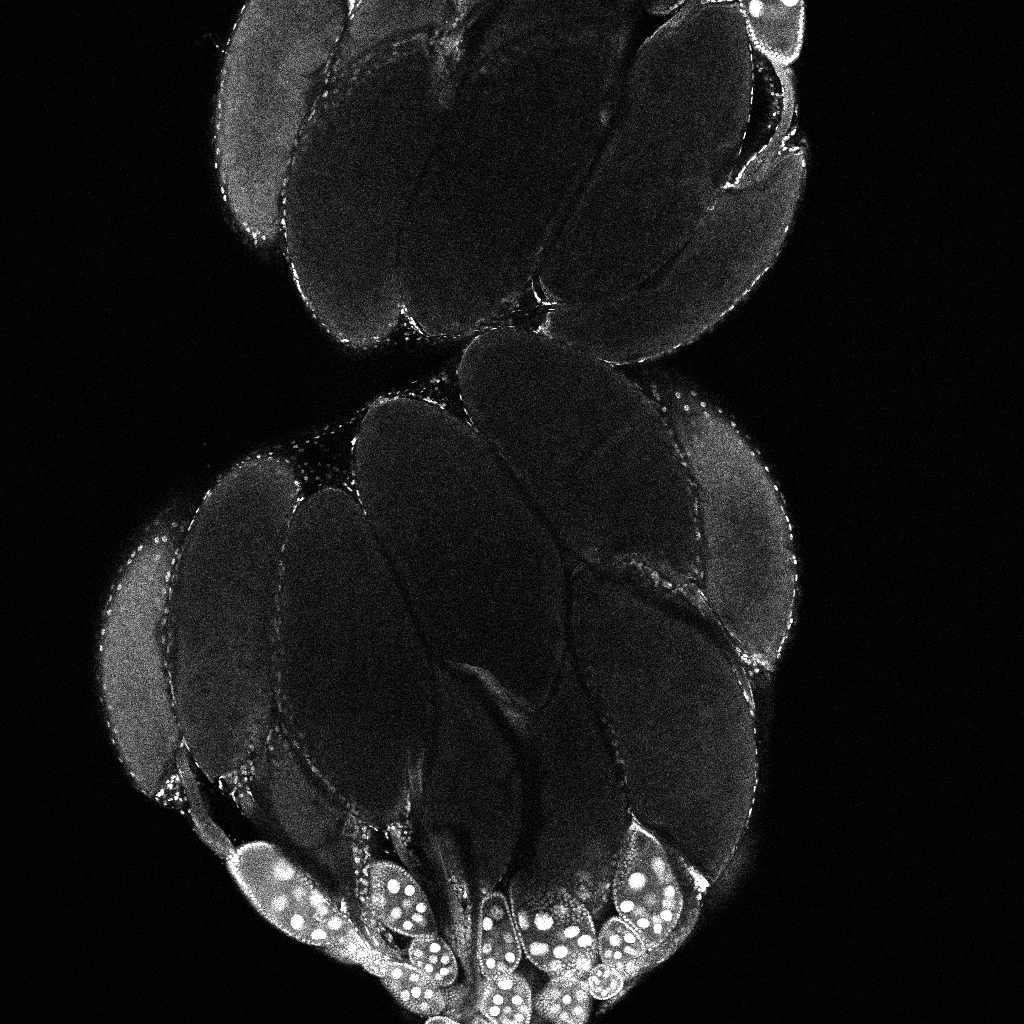

Supplement: Supplementary file 65 — Original Data [file 41419_2022_5195_MOESM65_ESM.tif]
